# In situ graphene-seq: spatial transcriptomics and chronic electrophysiological characterization of tissue microenvironments

**DOI:** 10.1038/s41467-026-73883-7

**Published:** 2026-06-19

**Authors:** Jaeyong Lee, Wenbo Wang, Qiang Li, Zuwan Lin, Ren Liu, Zefang Tang, Junya Aoyama, Richard T. Lee, Xiao Wang, Jia Liu

**Affiliations:** 1 John A. Paulson School of Engineering and Applied Sciences, Harvard University, Boston, MA USA; 2 Broad Institute of MIT and Harvard, Cambridge, MA USA; 3 Department of Stem Cell and Regenerative Biology, Harvard University, Cambridge, MA USA; 4 Division of Cardiovascular Medicine, Department of Medicine, Brigham and Women’s Hospital and Harvard Medical School, Boston, MA USA; 5 Department of Chemistry, Massachusetts Institute of Technology, Cambridge, MA USA

**Keywords:** Bionanoelectronics, Electronic properties and devices, Bionanoelectronics, Biomedical engineering

## Abstract

Biological systems comprise diverse, interconnected cell types whose functional dynamics and molecular identities are tightly coupled, yet difficult to capture simultaneously at high spatiotemporal resolution. Electrophysiology provides real-time measurements of cellular activity but with limited molecular context, whereas transcriptomics profiles gene expression without dynamic physiological readouts. Here, we introduce in situ graphene-sequencing, a platform that integrates chronic electrophysiology with imaging-based, spatially resolved transcriptomics (STARmap). The system combines stretchable mesh nanoelectronics for long-term, single-cell-level interfacing with transparent graphene/poly(3,4-ethylenedioxythiophene) polystyrene sulfonate electrodes, enabling seamless integration of electrical recording and optical imaging. By coupling electrophysiology with STARmap, the platform enables multimodal analysis of heterogeneous tissue microenvironments. We demonstrate in situ graphene-sequencing by charting multimodal profiles of human-induced pluripotent stem cell-derived cardiomyocyte and endothelial cell co-cultures, examining how spatial heterogeneity is associated with electrophysiological activity and gene expression. This approach provides an integrative framework for studying how tissue microenvironments shape cell behavior and molecular states.

## Abstract (web summary)

Biological tissues exhibit coupled electrical activity and gene expression that are difficult to measure simultaneously. Here, authors introduce in situ graphene sequencing, integrating electrophysiology with spatial transcriptomics to enable multimodal characterisation of tissue microenvironments.

## Introduction

Understanding the relationship between spatially resolved gene expression, cell types, and their microenvironmental niches is fundamental to unraveling the complexities of tissue function^1^. Biological tissues are composed of heterogeneous cell populations that interact dynamically, influencing one another and driving tissue-level activities^1–3^. The spatial organization of these cells, their gene expression profiles, and the microenvironmental niches they inhabit—collectively referred to here as the cell microenvironment—play critical roles in processes such as development, regeneration, and disease progression^4–8^. Mapping spatially resolved gene expression together with cell types and their niches allows the identification of how microenvironmental differences contribute to molecular and functional heterogeneity within tissues, with important implications for neuroscience, cancer biology, and regenerative medicine.

However, linking these spatially distinct molecular features to physiological outputs such as electrical activity remains technically challenging. Electrical recording methods have uncovered cellular responses and electrophysiological dynamics at the single-cell level^9,10^. For example, high-density electrode arrays with single-cell resolution have been instrumental in probing the coordinated cellular activities underlying sensory, motor, and cognitive functions^11–13^. Moreover, long-term single-cell-resolution monitoring has revealed how cellular activity and connectivity evolve in response to environmental stimuli, development, and aging^14–16^. Despite their utility, electrophysiological recordings alone lack the molecular context necessary to describe the specific types and states of cells underlying these changes. Spatial transcriptomics, on the other hand, has been widely employed to characterize cell types and states within their spatial context^17^. However, the molecular profiling of fixed samples is often insufficient to capture dynamic cellular behaviors. Synergistic approaches combining electrophysiology and transcriptomics have enabled precise cell-type classification and revealed correlations between gene expression and electrophysiological properties^18–23^. However, existing multimodal techniques remain limited in their spatial and temporal resolution.

To address these limitations, we previously introduced in situ electro-sequencing, which combines chronic electrophysiology with spatially resolved transcriptomics^24^. Using tissue-like mesh electronics, this platform enables long-term stable, single-cell-level electrical recordings within three-dimensional (3D) microtissues, while imaging-based in situ sequencing (STARmap) extracts gene expression profiles from electrically recorded cells^25^. This multimodal approach allows high-throughput mapping of both electrophysiological and transcriptomic states at the single-cell level. While this approach established the foundation for temporal multimodal analysis—linking long-term electrical dynamics with molecular characterization—the use of opaque metal electrodes in mesh electronics introduced significant challenges for maintaining spatial integrity throughout the workflow. Imaging recorded cells on non-transparent electrodes required additional experimental steps, including detaching and flipping tissues, which introduced handling and registration challenges and limited the application to ultra-thin tissue slides. Because mesh electronics accommodate the dynamic stretching and bending of cellular networks during development, the electrodes themselves reorient in 3D. Since optical access is restricted to electrode orientations that do not obstruct the imaging path, imaging outcomes become highly variable, particularly within complex 3D structures such as organoids and spheroids.

**Fig. 1:**
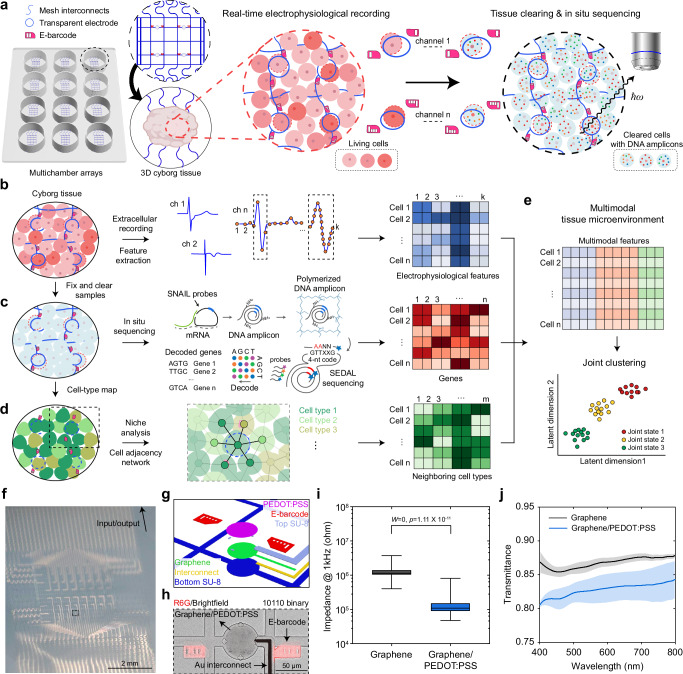
Overview of in situ graphene-seq for multimodal tissue analysis. **a**–**e** Schematics of the in situ graphene-seq workflow. **a** Stretchable mesh nanoelectronics with transparent graphene/PEDOT:PSS electrodes integrated with stem cell-derived microtissues enable chronic electrophysiological recordings, followed by tissue clearing and in situ sequencing. Fluorescent E-barcodes enable alignment of electrophysiological recordings with spatial gene expression mapping. Elements of this panel were created in BioRender. Lee, J. (2026) https://BioRender.com/vioyedg. **b** Multichannel extracellular recordings are processed to extract electrophysiological features. **c** In situ sequencing generates spatially resolved single-cell gene expression profiles. **d** Cell-type maps and niche analyses are constructed based on spatial proximity and cell-type composition. **e** Joint embedding and clustering of multimodal features integrates electrophysiological, molecular, and cell niche data, providing a comprehensive understanding of tissue microenvironments. **f** Photograph of a representative mesh nanoelectronics array with transparent electrodes. **g** Schematic of the electrode structure. **h** Bright-field image of a representative graphene/PEDOT:PSS electrode. **i** Box-and-whisker plot showing electrochemical impedance of electrodes at 1 kHz measured before and after PEDOT:PSS deposition. Center lines indicate the median, box bounds represent the 25th–75th percentiles, and whiskers denote the minima and maxima. Statistical significance was assessed using a two-tailed Wilcoxon matched-pairs signed-rank test (*n* = 61 electrodes; each electrode measured before and after PEDOT:PSS deposition). **j** Optical transmittance of electrodes before and after PEDOT:PSS deposition. Data are presented as mean ± S.D. (*n* = 5 electrodes per condition). Source data are provided as a Source Data file.

Here, we introduce in situ graphene-sequencing (graphene-seq), a multimodal in situ platform that seamlessly integrates chronic electrophysiology with high-throughput optical profiling technologies (Fig. 1a). The stretchable mesh structure enables long-term, single-cell-level interfacing across tissues, facilitating chronic electrophysiological analyses (Fig. 1b). By incorporating transparent graphene/PEDOT:PSS (poly(3,4-ethylenedioxythiophene) polystyrene sulfonate) electrodes into the mesh, the platform maintains optical clarity, enabling direct imaging of cells on top of the electrodes regardless of orientation.

After electrophysiological recordings, imaging-based in situ sequencing is performed to obtain gene expression profiles (Fig. 1c). Reconstructed cell-type maps provide molecularly defined cell types of electrically recorded cells, as well as the local cell compositions, allowing the study of electrophysiological and molecular phenotypes within specific microenvironmental niches (Fig. 1d). Fluorescent E-barcodes, fabricated adjacent to the electrodes, bridge electrophysiological recordings with imaging data, facilitating comprehensive multimodal profiling of tissue microenvironments (Fig. 1e). This integrated platform supports the entire workflow—from culturing tissues with devices and recording electrical activities to tissue clearing and high-resolution optical imaging—offering a scalable solution for high-throughput, multimodal profiling on a single platform.

## Results

### Design and fabrication of stretchable and transparent mesh nanoelectronics

The stretchable and transparent mesh nanoelectronics consist of a serpentine mesh–structured SU-8 passivation layer and gold interconnects, as described in previous reports^14,16^. (Fig. 1f, Supplementary Fig. 1a). To incorporate transparent electrodes into the mesh platform, we transferred and patterned 3–5 layers of graphene on top of the interconnects (“Methods”, Supplementary Fig. 1b, c). The top SU-8 layer passivates the interconnects, exposing only the graphene electrodes for electrophysiological recordings. We fabricated fluorescent E-barcodes next to each electrode for pairwise mapping of electrophysiology and imaging data (Supplementary Fig. 1d–f).

The electrochemical impedance of electrodes is crucial for the signal-to-noise ratio (SNR) in electrophysiological recordings^26^. While graphene microelectrodes offer transparency, they exhibit relatively high electrochemical impedance^27–29^. To reduce impedance while maintaining transparency, we electrochemically deposited PEDOT:PSS onto graphene electrodes^30^ (Fig. 1g, h). This process introduces an inherent trade-off between optical transparency and electrochemical performance: increasing PEDOT:PSS deposition lowers impedance but also decreases optical transmittance due to increased absorption associated with thicker PEDOT:PSS films^31,32^.

Deposition was precisely controlled by maintaining a constant current density and adjusting deposition time (“Methods”, Supplementary Fig. 2a, b). We optimized the deposition time to balance optical transmittance and electrochemical impedance (Supplementary Fig. 2c–f). This optimization reduced the electrochemical impedance at 1 kHz from 1319 ± 600 kΩ to 168.7 ± 156.1 kΩ after depositing PEDOT:PSS (mean ± S.D., Fig. 1i), while maintaining transmittance above 80% across the visual spectrum, making it compatible with most bioimaging techniques (Fig. 1j). Furthermore, the graphene/PEDOT:PSS electrodes demonstrated stable electrochemical performance across different samples (Supplementary Fig. 2g) and maintained electrochemical and structural integrity for over 60 days of incubation in 1X PBS at 37 °C (Supplementary Fig. 2h, i).

The fabrication process is readily scalable, as all steps are compatible with conventional 2D microfabrication techniques. Moreover, recent advances in graphene production and transfer techniques enable the reliable, large-scale integration of graphene electrodes into such devices^33–35^, supporting the potential of in situ graphene-seq for broad and customizable applications.

### Electrical and optical performance on cultured tissues

We integrated stretchable and transparent mesh electronics with 3D cardiac organoids using established methods (Supplementary Fig. 3a–c)^16^. Initially, the devices were integrated with 2D sheets of human-induced pluripotent stem cell (hiPSC)-derived cardiomyocytes (CMs) with a Matrigel layer positioned beneath the device. During differentiation and self-organization, the transition from 2D to 3D in the formation of cardiac organoids folded the devices into 3D structures, ultimately embedding them within the 3D organoids.

**Fig. 2:**
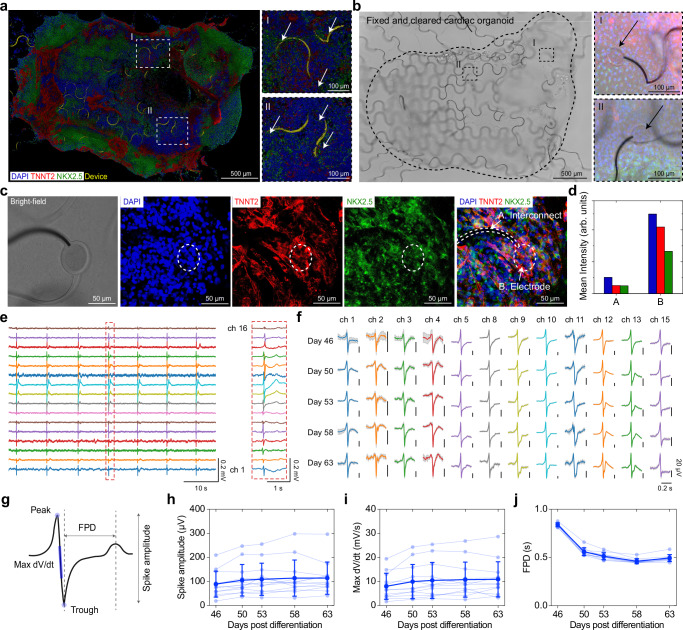
3D cardiac organoid integration and long-term electrophysiological recordings. **a**, **b** Cardiac organoids integrated with mesh nanoelectronics. **a** 3D confocal reconstruction. Right panels show zoom-in views of the dashed-box regions in the left panel. White arrows highlight meshes embedded within the organoid. **b** Bright-field (BF) images. Right panels show zoom-in views of the dashed-box regions in the left panel. Black arrows indicate transparent graphene/PEDOT:PSS electrodes. **c** BF and maximum-intensity projection confocal images of immunohistochemically stained cardiomyocytes surrounding a representative graphene/PEDOT:PSS electrode. Regions A and B indicate areas over metal interconnect and graphene/PEDOT:PSS electrode, respectively. **d** Quantification of mean pixel intensity for three fluorophores over the metal interconnect region (A) and the graphene/PEDOT:PSS electrode region (B) shown in (**c**). **e** Representative voltage traces recorded from a 16-electrode array. Right panel shows zoom-in views of spike waveforms. **f** Spike waveforms (mean ± S.D.) extracted from the same electrodes across five recording sessions. **g** Schematic of waveform features extracted for analysis: spike amplitude, maximum dV/dt, and field potential duration (FPD). **h**–**j** Temporal tracking of waveform features across five recording sessions: spike amplitude (**h**), maximum dV/dt (**i**), and FPD (**j**). Data are presented as mean ± S.D. Sample sizes: **h** *n* = 12; **i** *n* = 12; **j** *n* = 10 electrodes. Each dot represents a feature extracted from the mean waveform of an individual electrode. Source data are provided as a Source Data file.

To confirm the integration and transparency of graphene/PEDOT:PSS electrodes within organoids, we performed tissue fixation, clearing, and immunostaining, followed by confocal microscope imaging (“Methods”, Fig. 2a, b). The 3D reconstructed images show the mesh electronics forming interwoven structures within the cellular networks (Fig. 2a), while bright-field (BF) images show the spatial distribution of electrodes across the organoids (Fig. 2b). Unlike the metal interconnect layer, which blocks optical signals, the graphene/PEDOT:PSS electrodes allow imaging of fluorescence markers directly above the electrodes (Fig. 2c, d).

The previous approach, which relied on opaque metal electrodes, required additional procedures such as detaching the tissue/electronics hybrid, flipping the sample, and transferring it to another plate for imaging electrically recorded cells (Supplementary Fig. 3d–f). These steps introduced substantial handling variability and could disrupt the native cell microenvironment due to mechanical stress during detachment, as well as hydrodynamic forces during flipping and transfer. In addition, the sequential nature of this workflow created a linear bottleneck, where experimental time scaled directly with the number of samples. The orientation of electrodes further constrained image acquisition of recorded cells. By incorporating transparent graphene/PEDOT:PSS electrodes, we eliminated these additional steps and enabled both electrophysiological recordings and imaging on a single platform, while preserving the original spatial localization of the tissue (Supplementary Fig. 3g–i). The removal of per-sample handling, combined with unobstructed optical access to recorded cells regardless of electrode orientation, facilitates high-throughput multimodal analyses within 3D tissues.

Next, we evaluated the recording stability of the distributed graphene/PEDOT:PSS electrodes in hiPSC-derived cardiac organoids. Voltage traces from 16 representative channels, recorded 22 days after device integration, are shown in Fig. 2e. Recordings were performed across five sessions using the same organoid, during which spike waveforms from effective channels were extracted (Fig. 2f). The graphene/PEDOT:PSS electrodes demonstrated stable extracellular field potential recordings throughout the organoid over 2 weeks. Furthermore, the stretchable and transparent mesh electronics enabled longitudinal tracking of waveform features, including spike amplitude, maximum dV/dt, and field potential duration (FPD), across 3D organoids (Fig. 2g). No significant changes were observed in spike amplitudes or maximum dV/dt over time (Fig. 2h, i), while a reduction in FPD was noted between days 46 and 50 post-differentiation (Fig. 2j). These results highlight the long-term recording stability of the stretchable and transparent mesh electronics, enabling detailed monitoring of electrophysiological dynamics in 3D tissue models.

### Multimodal mapping of electrophysiology, transcriptomics, and cell niches

In situ graphene-seq integrates electrophysiological recordings with imaging-based in situ sequencing on a single platform, eliminating intermediate handling steps and preserving spatial fidelity throughout the processes. To establish a streamlined framework for multimodal data acquisition, we first optimized this workflow using homogeneous hiPSC-derived cardiac microtissues, allowing direct evaluation of multimodal data integration without confounding spatial variability.

We integrated cardiac microtissues with stretchable and transparent mesh electronics to obtain extracellular recordings, which were processed to extract electrophysiological features (Methods). Immediately after recording, the same tissue/electronics hybrid was fixed and processed for in situ complementary DNA (cDNA) library preparation and sequencing, enabling spatially resolved transcriptomic profiling in situ (Methods). Consistent with the optical transparency of the electrodes, imaging signals from cells above the electrodes were largely preserved, whereas metal electrodes exhibited noticeable signal attenuation (Supplementary Fig. 4). Fluorescent E-barcodes positioned adjacent to electrodes enabled pairwise mapping between sequencing reads and electrical recordings.

To link molecular and electrophysiological information, we optimized 3D cell segmentation and assigned amplicon reads to individual cells (“Methods”, Supplementary Fig. 5). Major cell types were identified by clustering transcriptomic profiles, and electrically recorded cells were localized by spatial alignment of segmented cells with electrode positions. This workflow established a direct mapping between molecular signatures, local cellular environments, and electrophysiological recordings across the integrated cardiac microtissue (Supplementary Fig. 6).

### Capturing electrophysiological and molecular heterogeneity within spatially heterogeneous tissue

The spatially resolved, multimodal integration workflow of in situ graphene-seq makes it uniquely suited for investigating spatially heterogeneous tissues, where molecular identity and electrophysiological phenotype may diverge across the tissue. To validate whether our platform can resolve such spatial heterogeneity, we designed a controlled and biologically interpretable heterogeneous tissue model.

**Fig. 3:**
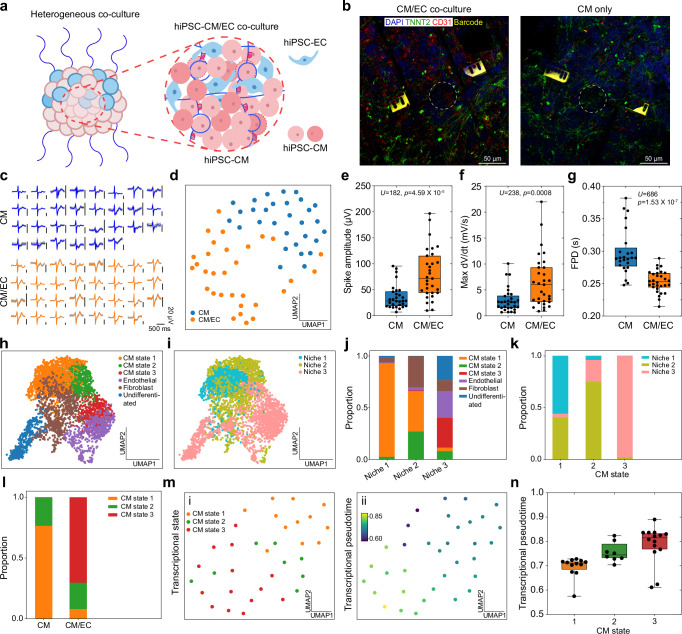
Spatial heterogeneity in hiPSC-CM/hiPSC-EC co-cultured microtissues. **a** Schematic illustration of spatially heterogeneous cell culture, with hiPSC-derived cardiomyocytes (hiPSC-CMs) co-cultured with hiPSC-derived endothelial cells (hiPSC-ECs) (CM/EC) on one side of the device and hiPSC-CMs cultured alone (CM) on the other side. Elements of this panel were created in BioRender. Lee, J. (2026) https://BioRender.com/9q873ty. **b** Maximum-intensity projection confocal images of immunohistochemically stained cells near electrodes in the CM/EC co-culture region and the CM only region. **c** Extracted spike waveforms (mean ± S.D.) from electrodes in the CM/EC and CM only regions. **d** UMAP embedding of mean waveforms from individual channels, color-coded by electrode location. **e**–**g** Box-and-whisker plots comparing waveform features between the CM/EC and CM only regions, including spike amplitude (**e**), maximum dV/dt (**f**), and field potential duration (FPD) (**g**). Center lines indicate the median, box bounds represent the 25th–75th percentiles, and whiskers denote the minima and maxima. Statistical significance was assessed using a two-tailed Mann-Whitney *U* test. Sample sizes: **e**, **f** CM (*n* = 30), CM/EC (*n* = 31) electrodes; **g** CM (*n* = 25), CM/EC (*n* = 30) electrodes. Each dot represents a feature extracted from the mean waveform of an individual electrode. **h**, **i** Classification of hiPSC-CM states and cell types (**h**) and molecular niches (**i**) based on transcriptional profiles. **j** Bar plots showing the proportions of cell types and states within each molecular niche. **k** Distribution of molecular niches associated with different hiPSC-CM states. **l** Distribution of hiPSC-CM states in the CM only and CM/EC regions. **m** UMAP embedding of gene expression profiles of electrically recorded hiPSC-CMs, color-coded by cell states (i) and transcriptional pseudotime (ii). **n** Box-and-whisker plot showing the distribution of transcriptional pseudotime across different hiPSC-CM states. Center lines indicate the median, box bounds represent the 25th–75th percentiles, and whiskers denote the minima and maxima. Sample sizes: CM_s1 (*n* = 12), CM_s2 (*n* = 8), CM_s3 (*n* = 14) cells. Source data are provided as a Source Data file.

We engineered spatial heterogeneity by co-culturing hiPSC-CMs and hiPSC-derived endothelial cells (hiPSC-ECs), building on prior studies showing that co-culture with hiPSC-ECs promotes the electrical maturation of hiPSC-CMs^23^. To create a defined spatial pattern, we seeded hiPSC-CMs and hiPSC-ECs at a 3:1 ratio on one side of the mesh electronics, while seeding only hiPSC-CMs on the opposite side (Methods, Fig. 3a). We expected this design to yield interpretable and spatially localized differences in the electrical and molecular signatures of hiPSC-CMs. To validate the differences in cell composition, we performed immunohistochemical analysis. Staining for cardiac troponin T (TNNT2) and cluster of differentiation 31 (CD31) confirmed the intended cellular distributions, with higher densities of CD31-positive hiPSC-ECs localized to the co-culture region (Fig. 3b).

To compare electrophysiological characteristics across regions, we recorded signals, extracted representative waveforms, and aligned them with electrode positions (Methods). Channels 1–32 recorded signals from the CM/EC co-culture region, while channels 33–64 captured data from the CM-only region (Fig. 3c). The two domains exhibited coordinated rhythmic activity with a consistent conduction delay across the interface (Supplementary Fig. 7), indicating partial electrical coupling between regions, which is consistent with the intended design of a spatially heterogeneous yet functionally integrated tissue model. Projecting the representative waveforms from each channel into a 2D Uniform Manifold Approximation and Projection (UMAP) embedding revealed distinct groupings corresponding to the CM-only and CM/EC co-culture regions (Fig. 3d). Waveform feature analysis indicated enhanced electrical maturation of hiPSC-CMs in the CM/EC co-culture region, with higher spike amplitudes, greater maximum dV/dt, and shorter FPD^23,36,37^, compared to the CM-only region (Fig. 3e–g).

In situ sequencing identified multiple CM states as well as ECs and fibroblasts, annotated using canonical lineage markers (e.g., *MYL7*, *MYH6*, *ACTN2*, *RYR2* for CMs; *PECAM1* (CD31) and *CDH5* for ECs; *COL1A1* and *COL3A1* for fibroblasts), along with a small population of undifferentiated or progenitor-like cells (Fig. 3h, Supplementary Fig. 8). Cell niches were characterized by analyzing the local cellular composition surrounding each cell and organizing these data into a cell-by-composition matrix for dimensionality reduction and clustering (Methods). This analysis identified three primary cell niches, each defined by distinct cell-type proportions (Fig. 3i, j). Notably, specific CM states were associated with different niches: CM state 3 (CM_s3) was predominantly enriched in Niche 3, while CM state 1 (CM_s1) localized mainly to Niche 1 (Fig. 3k). CM-only and CM/EC co-culture regions also displayed distinct proportions of CM states, with CM_s1 enriched in CM-only regions and CM_s3 in CM/EC co-culture regions (Fig. 3l). CM state 2 (CM_s2) was present in both conditions.

Gene expression analysis highlighted distinct molecular characteristics among CM states (Supplementary Fig. 9). CM_s1 exhibited higher expression of genes such as *PDK1* and *HAND2*, which are associated with early developmental stages^38,39^. In contrast, CM_s2 and CM_s3 showed upregulation of ion channel genes associated with excitation-contraction coupling and electrophysiological properties. For example, potassium channel genes, including *KCNE2*, *KCNK3*, and *KCNA4*, were highly expressed, along with *PIEZO1*, a mechanosensitive ion channel involved in responses to mechanical stress^40^. CM_s3 also exhibited increased expression of adhesion-related genes, such as *VCL*, *ITGB5*, and *CDH2*, as well as gap junction-related genes, including *GJA1* (Connexin 43) and *GJA5* (Connexin 40), suggesting enhanced electrical coupling.

To further characterize variation in molecular states, we performed pseudotime trajectory analysis, which estimates continuous cell states based on transcriptomic data (Methods, Fig. 3m)^41–43^. The results showed a clear ordering along the pseudotime trajectory, with molecular pseudotime increasing from CM_s1 to CM_s2 and then to CM_s3 (Fig. 3n). The correspondence between electrical and molecular heterogeneity in hiPSC-CMs across different tissue regions highlights the capability of in situ graphene-seq to capture both electrophysiological and molecular heterogeneity within tissues.

### Integrative analysis of electrophysiological and molecular characteristics in spatially resolved cell niches

**Fig. 4:**
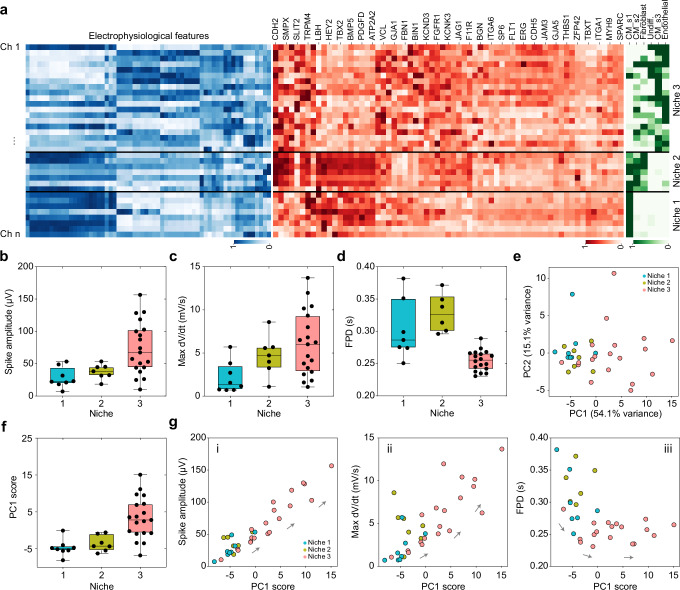
Electrophysiological characterization of molecularly defined cell niches. **a** Concatenated heatmaps displaying electrophysiological features, selected gene expressions, and local cell compositions of identified cells, grouped by molecularly defined niches. Colors represent z-scored values. **b**–**d** Comparative analysis of waveform features in cardiomyocytes (CMs) across the three cell niches: spike amplitude (**b**), maximum dV/dt (**c**), and field potential duration (FPD) (**d**). Center lines indicate the median, box bounds represent the 25th–75th percentiles, and whiskers denote the minima and maxima. Sample sizes: **b**, **c** Niche 1 (*n* = 8), Niche 2 (*n* = 7), Niche 3 (*n* = 19) electrodes; **d** Niche 1 (*n* = 7), Niche 2 (*n* = 6), Niche 3 (*n* = 18) electrodes. Each dot represents a feature extracted from the mean waveform of a single electrode. **e** PCA embedding of electrophysiological features from electrically recorded CMs, color-coded by cell niche. **f** Box-and-whisker plot showing the distribution of PC1 scores across the three niches. Center lines indicate the median, box bounds represent the 25th–75th percentiles, and whiskers denote the minima and maxima. Sample sizes: Niche 1 (*n* = 8), Niche 2 (*n* = 7), Niche 3 (*n* = 19) electrodes. **g** Scatter plots showing correlations between PC1 scores and waveform features: spike amplitude (i), maximum dV/dt (ii), and FPD (iii). Each dot represents features from a single electrode, color-coded by niche identity. Higher PC1 scores correspond to increased spike amplitude, greater maximum dV/dt, and shorter FPD, indicating progression toward more electrically matured states. Source data are provided as a Source Data file.

Spatially resolved in situ RNA sequencing enables precise cell-type classification and niche analysis, facilitating the identification of spatially organized tissue regions^8,44^. In situ graphene-seq extends this capability by providing electrophysiological characterization of cells within molecularly defined regions (Fig. 4a), allowing the study of electrophysiological distinctions associated with local microenvironments.

We first analyzed waveform features of electrically recorded cells across molecularly defined niches (Fig. 4b–d). Spike amplitude, maximum dV/dt, and FPD exhibited distinct properties among Niche 1, Niche 2, and Niche 3, reflecting region-specific electrophysiological states. Notably, spike amplitude and maximum dV/dt increased from Niche 1 to Niche 2 and Niche 3. To quantify this progression, we used the first principal component (PC1) derived from electrophysiological features as a linear progression score, which captured 54.1% of the total variance and represented the dominant electrophysiological gradient across niches (“Methods”, Fig. 4e). Cells in Niche 3 displayed higher PC1 scores, followed by those in Niche 2 and Niche 1 (Fig. 4f). Scatterplots correlating PC1 scores with waveform features demonstrated increasing spike amplitude, greater maximum dV/dt, and shorter FPD with increasing PC1 scores (Fig. 4g). Together, these results indicate that CMs in Niche 3 exhibit electrophysiological features associated with more mature electrophysiological states, followed by those in Niche 2 and Niche 1. This highlights the capability of in situ graphene-seq to capture electrophysiological distinctions aligned with molecularly defined niches.

**Fig. 5:**
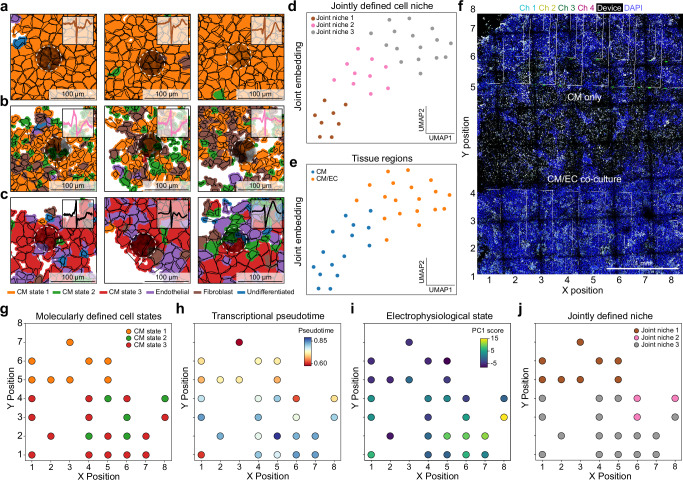
Multimodal cell niches and tissue annotation. **a**–**c** Representative cell-type maps of electrically recorded cardiomyocytes (CMs) in molecularly defined Niche 1 (**a**), Niche 2 (**b**), and Niche 3 (**c**), with electrodes and corresponding recorded waveforms overlaid. **d**, **e** Joint embedding of electrophysiological features, gene expression, and local cell composition into 2D UMAP space, color-coded by jointly defined cell niches (**d**) and tissue regions (CM only and CM/EC co-culture regions; ECs, endothelial cells) (**e**). **f** Representative in situ sequencing image of a heterogeneous microtissue containing CM only and CM/EC regions, with relative X and Y positions of electrodes indicated. **g**–**j** Spatial mapping of relative electrode positions across the tissue shown in (**f**). Each dot represents an electrode, color-coded by the molecularly defined states of electrically recorded hiPSC-CMs (**g**), transcriptional pseudotime (**h**), electrophysiological state (PC1 score) (**i**), and jointly defined cell niche (**j**).

In situ graphene-seq integrates spatial mapping of gene expression, cell-type distributions, and electrophysiological recordings, providing an integrated framework for understanding tissue microenvironments. Representative electrodes positioned on cell-type maps, along with the corresponding waveforms recorded from these electrodes, highlight molecular and electrophysiological heterogeneity across tissue regions (Fig. 5a–c). Leveraging these multimodal datasets enables a more comprehensive approach to studying tissue organization, extending molecular-based analyses into an integrative multimodal framework. As a demonstration, we jointly defined cell niches by combining electrophysiological features, gene expression profiles, and local cell composition data (“Methods”, Fig. 5d, e). Joint embedding of these features delineated distinct niches (Fig. 5d) and separated the two culture conditions: CM only and CM/EC co-culture (Fig. 5e).

Since our platform allows precise localization of electrodes within the tissue model (Fig. 5f), spatial information can be mapped back onto the original tissue architecture. This provides an integrated perspective on how spatial organization relates to local electrophysiological and molecular properties. By identifying the relative positions of electrodes within the sample, we defined regional profiles within heterogeneously cultured tissues, enabling investigation of molecularly defined cell states, transcriptional pseudotime, and electrophysiological traits, as well as a preliminary demonstration of multimodal niche classification (Fig. 5g–j).

Beyond descriptive analysis, this multimodal platform potentially enables cross-modal inference between molecular and electrophysiological domains. To explore this capability, we performed proof-of-concept prediction of spatial niche identity using either molecular or electrophysiological features (Methods). A random forest classifier trained on gene expression data achieved 98.24% accuracy on a held-out test set (Supplementary Fig. 10a, b). Using electrophysiological features alone, the classifier predicted niche identity with 82.35% accuracy (Supplementary Fig. 10c). Together, these results suggest that electrophysiological recordings contain information associated with molecularly defined niches, highlighting the potential for cross-modal inference enabled by this platform.

## Discussion

In this study, we present in situ graphene-seq, a multimodal platform that integrates transparent mesh nanoelectronics with imaging-based in situ sequencing to simultaneously capture electrical activity and spatial transcriptomics within intact 3D tissues. By enabling electrophysiological recordings and molecular profiling on the same sample, without disturbing tissue architecture, this approach streamlines multimodal data acquisition and greatly improves spatial fidelity.

By applying in situ graphene-seq to engineered heterogeneous microtissues with predictable spatial organization, we resolved spatially organized multimodal heterogeneity, including distinct electrophysiological characteristics and the distribution of CM states across the tissue. Leveraging this multimodal information, we performed niche-level analysis linking local electrophysiological properties to molecularly defined niches and demonstrated multimodal niche classification by integrating electrophysiological features, gene expression profiles, and local cell composition. Since these spatial patterns were anticipated based on the co-culture design, their recapitulation demonstrates that in situ graphene-seq can co-register functional and molecular information within intact tissues. The demonstrated stability of long-term recordings further positions the system for future longitudinal multimodal studies.

There are several promising directions to further expand the capabilities of in situ graphene-seq. Integration with real-time functional measurements, including mechanophysiological readouts from graphene-based biosensors^45^, could further expand its dimensionality. Advances in spatial omics, including spatial transcriptomics with protein imaging^46^, transcriptome-translatome co-profiling, and sequencing of intact organoids or tissue blocks^47^, are also well positioned to synergize with this platform. Although this study focused on validating the platform in a relatively simple and predictable heterogeneous tissue model, applying in situ graphene-seq in vivo may enable multimodal mapping of brain and cardiac systems at high spatiotemporal resolution.

Longitudinal multimodal datasets generated by in situ graphene-seq will also be well suited for artificial intelligence (AI)-driven analyses, which have already demonstrated strong capabilities in extracting biological and biomedical insights from high-dimensional data^48–50^. Beyond the proof-of-concept demonstration in this study, such approaches may enable prediction of molecular states from electrical dynamics, monitoring of niche-dependent maturation, and elucidation of how multicellular composition, including support cell densities, influences electrophysiological and molecular maturation, potentially providing quantitative guidance for microtissue design.

Together, these advances position in situ graphene-seq as a versatile platform for multimodal tissue analysis, with the potential to deliver temporally and spatially resolved insights into complex biological systems.

## Methods

### Device fabrication

Fabrication steps of stretchable and transparent mesh nanoelectronics are as follows: (1) A 3-inch glass wafer (D263 Glass wafer, UniversityWafer) was cleaned using a piranha solution (3:1 mixture of sulfuric acid and 30% hydrogen peroxide), followed by rinsing with acetone, isopropyl alcohol (IPA), and deionized (DI) water. (2) Nickel (Ni) sacrificial layer patterning: Hexamethyldisilazane (HMDS, MicroChem) and LOR 3 A (MicroChem) were spin-coated at 3000 rpm and baked at 180 °C for 5 min. Photoresist S1805 (MicroChem) was spin-coated at 3000 rpm, baked at 115 °C for 1 min, exposed to 405 nm ultraviolet (UV) light at 40 mJ/cm², and developed using CD-26 developer (Microposit). The sample was descummed using O_2_ plasma (12 sccm flow, 50 W for 1 min, Samco PC-300). (3) A 100 nm-thick Ni layer was deposited using a Sharon thermal evaporator, followed by a standard lift-off process in remover PG (MicroChem). (4) Bottom SU-8 patterning: SU-8 2002 (MicroChem) was spin-coated at 4000 rpm, pre-baked at 65 °C for 2 min and 95 °C for 4 min and exposed to 365 nm UV light at 200 mJ/cm². The sample was post-baked at 65 °C and 95 °C for 2 min each, developed using SU-8 developer (MicroChem), and then hard-baked at 180 °C for 1 h. (5) Metal interconnect patterning: A lift-off pattern was created as described in step (2). Gold (Au) with chromium (Cr) adhesive layers (Cr/Au/Cr, 5/70/5 nm-thick) was deposited using an e-beam evaporator (Denton) and lifted off in remover PG (MicroChem). (6) Graphene wet transfer: 950 PMMA A4 (Kayaku Advanced Materials) was spin-coated at 3000 rpm on graphene on copper foil (ACS Material) and baked at 150 °C for 3 min. The backside graphene was optionally etched using O_2_ plasma (12 sccm flow, 50 W for 1 min). Copper was etched using a copper etchant (APS-100, Transene) for 2 h, followed by rinsing with DI water for three times, 30 min each. The freestanding graphene/PMMA film was transferred to the wafer and baked at 80 °C overnight. The PMMA layer was then removed using acetone. (7) Graphene patterning: HMDS and Photoresist S1813 (MicroChem) were spin-coated at 3000 rpm, baked at 115 °C for 1.5 min, exposed to 405 nm UV light at 150 mJ/cm², and developed using CD-26 developer. Graphene was etched using O_2_ plasma (12 sccm flow, 150 W for 3 min). (8) Top SU-8 passivation: Conditions described in step (4) were used. (9) E-Barcode patterning: A 0.004 wt% mixture of Rhodamine 6 G (Sigma-Aldrich) and SU-8 2000.5 (MicroChem) was spin-coated at 2000 rpm. The sample was pre-baked at 65 °C for 1 min and 95 °C for 3 min, exposed to 365 nm UV light at 60 mJ/cm², post-baked at 65 °C for 1 min and 95 °C for 2 min, developed in SU-8 developer, and hard-baked at 180 °C for 1 h. (10) Flexible flat cables (Molex) were connected to the input/output pads using a flip-chip bonder (Finetech). (11) A customized chamber was glued onto the wafer, and electrical components outside the chamber were encapsulated with a low-toxicity silicone adhesive (Kwik-Sil, World Precision Instrument). (12) The Ni sacrificial layer was released by adding ferric chloride (MG Chemicals) into the chamber, followed by etching for 2–3 h at 40 °C and rinsing with DI water several times.

PEDOT:PSS deposition: PEDOT:PSS was electrochemically deposited onto graphene electrodes to reduce impedance while preserving optical transparency. The device chamber was filled with an aqueous solution containing 0.01 M EDOT (Sigma-Aldrich) and 0.1 M PSS (Sigma-Aldrich) in DI water. Electrochemical polymerization was performed with the graphene electrode serving as the working electrode and a platinum (Pt) wire used as both the reference and counter electrode. A DC current (0.4 mA/cm²) was applied using SP-150 potentiostat (Bio-Logic). Deposition duration was varied from 2 to 10 s to evaluate the trade-off between electrochemical impedance and optical transmittance. Based on these measurements, a deposition time of 4 s was selected as the optimal condition for subsequent experiments.

### Electrical and optical characterization

Electrochemical impedance spectra (EIS) of graphene and graphene/PEDOT:PSS electrodes were measured using SP-150 potentiostat. The devices were immersed in 1X PBS with a silver/silver chloride (Ag/AgCl) reference electrode and a Pt counter electrode. A sinusoidal voltage with a 100 mV peak-to-peak amplitude was applied over a frequency range from 1 MHz to 1 Hz. The impedance at 1 kHz of the electrodes was characterized using the integrated functions of the Blackrock CerePlex and Intan RHD recording systems. Optical transmittance of the graphene and graphene/PEDOT:PSS electrodes was measured using CytoViva hyperspectral microscopy and visualized using locally weighted regression (LOWESS) or Savitzky-Golay filtering^51^. Data and statistical analyses were performed using GraphPad Prism.

### Cell culture and differentiation

Stem cell culture and cardiomyocyte differentiation: hiPSCs (IMR90-1) were obtained from the WiCell Research Institute (Madison, WI, USA), with mycoplasma testing performed by the vendor. The hiPSCs were maintained and cultured on Matrigel-coated 6-well plates in Essential 8 medium (Gibco). Culture medium was replaced daily to maintain cell pluripotency. Cells were passaged every 4 days using 5 mM EDTA (Invitrogen) in 1X PBS. 10 µM ROCK inhibitor (Tocris Bioscience) was added to the cell culture medium for the first 24 h post-passaging. CM differentiation was performed as follows^16,24^. hiPSCs at approximately 80% confluency were treated with RPMI 1640 (Gibco) supplemented with 2% B27 minus insulin (Gibco) and 12 µM CHIR99021 (BioVision) for 24 h. Subsequently, cells were incubated in RPMI 1640 with 2% B27 minus insulin for 48 h, followed by 48 h of treatment with RPMI 1640 containing 2% B27 minus insulin and 5 µM IWR1 (Cayman Chemical). Cells were then cultured in RPMI 1640 with 2% B27 minus insulin for 48 h before transitioning to RPMI 1640 supplemented with 2% B27 for extended culture. Successful differentiation was confirmed by spontaneous beating of the tissue, typically evident 2–3 days after changing the medium to RPMI 1640 with 2% B27. Culture medium was replaced every 48 h. hiPSC-ECs were cultured in Endothelial Cell Growth Medium-2 (Lonza). The media was changed every 4 days.

Integration of hiPSC-derived cardiomyocytes and endothelial cells with devices: Prior to cell integration, 10 µM ROCK inhibitor was added to the cell culture medium for 2 h. Devices were washed twice with 1X PBS. The devices were then incubated in a bind-silane solution (10% v/v acetic acid, 1% v/v bind silane in ethanol) for 3 h, followed by three washes with 90% ethanol. Subsequently, devices were incubated overnight in 0.01% w/v Poly-D-lysine hydrobromide solution (Sigma-Aldrich), washed three times with DI water, and coated with Matrigel solution (100 µg/mL, Corning) at 37 °C for 1 h. The devices were then chilled, and 50 µL of 10 mg/mL Matrigel solution was added from the edge. The device/Matrigel hybrid was incubated at 37 °C for 30 min.

hiPSC-CMs were dissociated into single-cell suspensions by incubation in 0.05% Trypsin-EDTA solution (Gibco) at 37 °C for 5 min. Cell viability was assessed using trypan blue staining (Gibco). Approximately 3–4 million hiPSC-CMs were resuspended in RPMI 1640 medium supplemented with 2% B27 and seeded onto the device/Matrigel hybrid.

For CM/EC heterogeneous co-culture, hiPSC-ECs were dissociated into single-cell suspensions using TrypLE Express (Gibco) at 37 °C for 3 min. hiPSC-CMs and hiPSC-ECs were mixed in a 3:1 ratio and resuspended in StemPro-34 SFM (Gibco) supplemented with 1X StemPro-34 Nutrient Supplement (Gibco), 1X GlutaMAX-I (Gibco), and 50 ng/mL human VEGF165 (GenScript). Two million cells were seeded to cover half of the device chamber. The chambers were tilted to prevent cell diffusion. After 24 h post-seeding, the remaining half of the device was seeded with 2 million hiPSC-CMs. All heterogeneous cultures were maintained in StemPro-34 SFM supplemented with 1X StemPro-34 Nutrient Supplement, 1X GlutaMAX-I, and 50 ng/mL human VEGF165. Culture medium was changed every 48 h.

### Immunohistostaining

Immunohistostaining was performed as follows^16^. Tissues were fixed with 4% paraformaldehyde (PFA) solution (Electron Microscopy Sciences) at 4 °C overnight. The samples were then incubated in a hydrogel solution (0.1% VA-044 (Fujifilm Wako) and 4% acrylamide (Bio-Rad) in 1X PBS) at 4 °C for 24 h, followed by hydrogel polymerization at 37 °C for 3 h. The polymerized samples were incubated in Electrophoretic Tissue Clearing solution (Logos Biosystems) at 37 °C for 48 h and subsequently rinsed several times with PBST (1X PBS + 0.1% Tween-20 (Millipore)). The samples were then incubated with primary antibodies diluted 1:200 at 4 °C for 4 days: Mouse Cardiac Troponin T (MA5-12960, Invitrogen) in combination with either Rabbit Nkx2.5 (ab97355, Abcam) or Rabbit CD31/PECAM-1 (NBP1-71663, Novus Biologicals). After washing with PBST, the samples were incubated with secondary antibodies diluted 1:600 at 4 °C for 2 days: Goat anti-rabbit Alexa 647 (A-21245, Invitrogen), Goat anti-mouse Alexa 594 (A-11005, Invitrogen). DAPI (4’,6-diamidino-2-phenylindole, Sigma-Aldrich) was added for nuclear staining, and the samples were incubated at 4 °C for 24 h. Finally, the samples were treated with an optical clearing solution and incubated at room temperature for 3 h. The prepared samples were stored at 4 °C until imaging, which was performed using a Leica TCS SP8 confocal microscope. Images were analyzed using Fiji software. Brightness and contrast were adjusted uniformly across images intended for comparison.

### Electrophysiological recording

Electrophysiological signals were recorded using an Intan RHD recording system (Intan Technologies). Devices were connected to Intan RHD recording headstages, with a Pt electrode immersed in the culture medium serving as both the ground and reference. Signals were recorded for 2 to 5 min inside a customized Faraday cage at a sampling rate of 10 kHz.

Raw data files were loaded and converted using Python scripts provided by Intan Technologies. Noise reduction was performed using either a 60 Hz low-pass filter (Butterworth, 4th order) or a 0.3–150 Hz band-pass filter, followed by Savitzky-Golay filter (window length = 101, polynomial order = 3). Spikes from each electrode were identified using the scipy.signal.find_peaks function from the SciPy library^52^, with the detection threshold set to 3–5 times the standard deviation from the mean baseline. Waveforms were aligned to the time point of maximum dV/dt. Waveform durations of 1.6 s were used for homogeneous hiPSC-CM cultures, while 0.9-s durations were used for hiPSC-CM/hiPSC-EC heterogeneous co-cultures to accommodate their faster beating rates.

### Electrophysiological signal processing and feature extraction

Further waveform analysis was performed using the Python package Scanpy^53^. Aligned waveforms from each electrode were averaged to generate representative waveforms. For visualization of electrophysiological heterogeneity (Fig. 3d), we performed dimensionality reduction of the representative waveforms from each electrode. We first applied Principal Component Analysis (PCA, scanpy.tl.pca) to capture the major sources of variation across waveforms. We then constructed a k-nearest neighbor (kNN) graph (scanpy.pp.neighbors) based on the PCA space. Finally, we computed a UMAP embedding (scanpy.tl.umap) to generate a low-dimensional representation that preserves local and global waveform relationships^54^. This approach enabled visualization of waveform-level differences across electrodes, revealing electrophysiological diversity within the tissue.

Waveform features, including amplitude, maximum dV/dt, and FPD, of the representative waveforms were analyzed using GraphPad Prism. Waveforms that did not exhibit discernable FPD peaks were excluded from the FPD analysis.

Electrophysiological features were extracted using a hierarchical median-based downsampling algorithm^24^. Each representative waveform was denoised using PyWavelets (wavelet = ‘db6’, level=3, factor=5)^55^ and then downsampled at two temporal resolutions. First, the entire waveform was downsampled to generate 22 features capturing the global waveform shape. Next, a 0.1 s high-resolution segment centered on the maximum dV/dt was downsampled to generate 40 features preserving fine-scale depolarization and early repolarization dynamics. The global and local representations (22 + 40 features) were concatenated to form a 62-dimensional feature vector for each electrode.

### In situ sequencing (STARmap)

In situ sequencing of RNA in tissue/electronics hybrids was performed as follows^24,25^. Tissue/electronics hybrids were fixed with 4% PFA for 15 min and permeabilized with 2 mL of methanol pre-chilled to −20 °C (Sigma-Aldrich), followed by incubation at −80 °C overnight. Samples were then quenched in PBSTR (1X PBS with 0.1% Tween-20, 0.1 U/µL SUPERase inhibitor (Thermo Fisher)) with 1% yeast tRNA (Invitrogen) and 100 mM Glycine (Sigma-Aldrich) for 15 min and then washed with PBSTR three times, 10 min each. Samples were incubated in 1X hybridization buffer containing 2X SSC (Sigma-Aldrich), 10% formamide (Calbiochem), 1% Tween-20, 20 mM Ribonucleoside Vanadyl Complex (RVC, New England Biolabs), 0.1 mg/mL yeast tRNA, 0.2% SDS (Calbiochem), and the SNAIL oligo pool (Integrated DNA Technologies) with 10 nM per oligo at 40 °C for 24 h. Samples were washed twice in PBSTR for 20 min each at 37 °C, followed by a 20-min wash in PBSTR with 4X SSC at 37 °C. Samples were then washed with PBSTR three times, 15 min each. Next, samples were incubated in ligation mixture (0.1 Weiss U/µL T4 DNA ligase (Thermo Fisher), 0.2 U/µL SUPERase inhibitor, 0.5 mg/mL bovine serum albumin (BSA, Invitrogen)) at room temperature overnight, followed by three 15-min washes in PBSTR. Samples were then incubated in the RCA mixture (0.2 Weiss U/µL Phi29 DNA polymerase (Thermo Fisher), 0.2 U/µL SUPERase inhibitor, 0.5 mg/mL BSA, 20 µM 5-(3-aminoallyl)-dUTP (Invitrogen), 250 µM dNTP mixture (Invitrogen)) for 6 h, followed by three 15-min washes in PBST (1X PBS with 0.1% Tween-20). Subsequently, samples were modified with 25 mM MA-NHS (Methylacrylic acid NHS ester, Sigma-Aldrich) at room temperature for 1 h, washed with PBST three times, and incubated in monomer solution (4% Acrylamide (Bio-Rad), 0.2% Bis-acrylamide (Bio-Rad), 2X SSC, 0.2% Tetramethylethylenediamine (Sigma-Aldrich)) at room temperature for 1 h. The solution was replaced with 30 µL of fresh monomer solution with 0.2% ammonium persulfate, covered with a Gel Slick (Lonza)-coated coverslip, and polymerized under nitrogen for 1 h. Samples were digested with 0.2 mg/mL proteinase K (Invitrogen) at 37 °C overnight then washed in PBST three times, 10 min each. Samples were then treated with 250 U/mL Antarctic phosphatase (New England Biolabs) at 37 °C for 30 min before sequencing. For the sequencing reaction, samples were treated with stripping buffer (60% formamide with 0.1% Triton-X) five times  for 10 min each, followed by six 10-min washes in PBST. Samples were then incubated in the sequencing mixture of different rounds (1:30 T4 DNA ligase, 1:100 BSA, 10 µM reading probes (Integrated DNA Technologies) and 5 µM decoding probes (Integrated DNA Technologies)) overnight. DAPI (Sigma-Aldrich) staining was performed according to the manufacturer’s protocol. Imaging was performed using a Leica TCS SP8 confocal microscope with 25× water immersion objective with 0.95 N.A.

Oligonucleotides for in situ sequencing were custom-designed and synthesized using oPools™ oligo pools (Integrated DNA Technologies) with standard desalting purification. For the hiPSC-CM-only sample, we utilized a 201-gene panel identical to that employed in our previous study^24^. For the hiPSC-CM/hiPSC-EC co-culture experiments, we designed a targeted 254-gene panel (Supplementary Data 1), curated to include key CM maturation markers, ion channels, structural genes, and major endothelial markers to ensure sufficient resolution for niche characterization.

To empirically validate compatibility with extended chemical and optical workflows required for large gene panels (>1000 genes), we performed a validation experiment using a 4-gene STARmap panel encoded with 5-bit barcodes (Supplementary Fig. 11, Supplementary Data 2). This encoding scheme requires six sequential imaging rounds for decoding, consistent with the workflow required for >1000-gene libraries.

### Data analysis

Imaging data analysis: Image processing and analysis of in situ sequencing data were conducted as follows. Raw fluorescence images underwent deconvolution using Huygens Essential software. Subsequently, contrast matching was performed using MATLAB’s imhistmatchn function, followed by background noise suppression via top-hat filtering. Global registration of images from five rounds was achieved using 3D fast Fourier transformation to maximize correlation coefficients, compensating for three-dimensional offsets. Further alignment was accomplished through non-rigid registration using MATLAB’s imregdemons function.

Individual amplicons were identified from first-cycle images using MATLAB’s imregionalmax function. Gene identity was determined by analyzing the dominant color within a [1, 1, 1] voxel volume surrounding each amplicon’s centroid and referencing the gene codebook. Cell segmentation was performed on the resulting gene amplicon point clouds using ClusterMap^56^.

Single-cell expression analysis utilized the Python package Scanpy. Quality control measures included filtering out cells with fewer than 5 gene counts or 5 unique genes and subsequently integrating with single cell RNA sequencing (scRNA-seq) data we previously published^23,57–59^. Data normalization involved log-transformation (log(x + 1)) and scaling to unit variance with zero mean. Batch correction across samples was implemented using the scanpy.pp.combat function prior to downstream analyses^60^.

Cell typing was performed as follows^23,24^. After segmentation and data preprocessing, we computed PCA (scanpy.tl.pca) and performed Leiden clustering (scanpy.tl.leiden)^61^ on the kNN graph constructed in PCA space (scanpy.pp.neighbors). Leiden clusters were annotated based on differential expression of genes associated with CMs, ECs, fibroblasts, or pluripotent identities. CM clusters were marked by *MYL7*, *MYH6*, *ACTN2*, and *RYR2*, and were further resolved into three subpopulations. ECs were identified by *PECAM1* (CD31) and *CDH5*, while fibroblasts were defined by *COL1A1* and *COL3A1*. An additional unidentified cluster was annotated as residual undifferentiated iPSCs or early primitive progenitors based on high expression of *SOX2*, *ZFP42* (REX1), and *EOMES*, together with minimal expression of other lineage-specific markers.

Electrode segmentation and recorded cell identification: Device components, including the graphene/PEDOT:PSS electrode, SU-8 passivation layers, and interconnect cable, were visualized using reflection mode imaging on a Leica TCS SP8 microscope. The reflection channel data underwent maximum intensity projection onto the x-y plane. Electrode segmentation was performed using the Segment Anything Model (SAM, version sam_vit_h_4b8939) by Meta^62^, with manual annotation of foreground and background regions to generate an electrode mask. This mask was then applied to the 3D reflection images to isolate the electrodes, which were subsequently converted to 8-bit (0–255) grayscale images and reconstructed as point clouds. A 3D convex hull was generated from the reconstructed electrode point clouds to define the electrode recording volume. To identify recorded cells, the distances between the centroid of each segmented cell and the electrode volume were calculated. Cells located within the convex hull and exhibiting the shortest distances to the electrode centroid were designated as the recorded cells, representing the most likely electrophysiological sources based on spatial proximity and expected biophysical coupling.

Niche analysis: For niche analysis, the neighborhood composition for each segmented cell derived from the in situ sequencing dataset was computed after cell typing. Based on previously published methods, a radius of 45 microns from the centroid of each cell was used to define the local microenvironment to include ~2 layers of cells from the center^56^. Within this radius, the composition of neighboring cell types was quantified, resulting in a cell-by-cell-type composition matrix. Dimensionality reduction and clustering were performed using the Scanpy package. PCA was applied to the composition matrix, and a kNN graph was computed in PCA space. Leiden clustering was then performed on the resulting kNN graph to identify groups of cells with similar neighborhood compositions. UMAP was used for visualization of the low-dimensional embeddings.

Pseudotime and trajectory analysis: Pseudotime analysis was conducted using the Slingshot package^43^ to infer the temporal progression of transcriptional states. Gene expression profiles of all sequenced CMs were embedded in a low-dimensional space using PCA. The resulting PCA embeddings, together with annotated cell type labels, were exported to R and used as input for Slingshot. The resulting pseudotime values were normalized to a [0, 1] range for comparability. Electrically recorded cells were selected after pseudotime calculation and visualized using UMAP. For the electrophysiological dataset, given the limited sample size, PC1, which accounted for 54.1% of total variance, was utilized as a linear progression score to represent electrophysiological state trajectories.

Joint multimodal analysis framework: To integrate electrophysiology, gene expression, and local cell composition into a unified analytical framework, features were pre-processed using z-score standardization or normalization as appropriate. The resulting feature matrices were concatenated column-wise to generate a unified feature matrix comprising 322 features (62 electrophysiological features, 254 genes, and 6 compositional features). PCA was performed on the combined matrix, followed by Leiden clustering and UMAP for visualization.

Prediction of spatial niche from molecular and electrophysiological features: We trained random forest classifiers to predict niche labels derived from spatial transcriptomic analysis using either gene expression profiles or electrophysiological features. For the molecular model, the input feature matrix consisted of gene expression values from CMs. Spatial niche identity was used as the classification target. Data were split into training and test sets using an 80/20 stratified split to preserve niche proportions across partitions. A random forest classifier was trained on the training set and evaluated on the held-out test set. Predictive performance was quantified by accuracy on the test set. Gene importance scores were obtained from the trained random forest model using Gini-based feature importance. For the electrophysiology model, the input feature matrix consisted of electrophysiological features from recorded CMs. Spatial niche identity was used as the target label. Due to the limited sample size, performance was evaluated using leave-one-out cross-validation (LOOCV), in which each sample was iteratively held out for testing while the model was trained on the remaining samples. A random forest classifier was trained within each fold, and overall accuracy was computed as the fraction of correctly classified samples across all LOOCV folds.

### Statistics and reproducibility

Study design and sample size: No statistical method was used to predetermine sample size. Sample sizes were determined based on the capacity of the electronic platforms and the yield of viable tissue/electronics interfaces. The images shown in Fig. 2a, b represent a single representative large-scale visualization of organoid/electronics hybrid. High-resolution micrographs in Fig. 2c are representative of *n* = 3 electrodes. Micrographs in Fig. 3b are representative of *n* = 3 electrodes per condition. Micrographs in Supplementary Fig. 4 are representative of *n* = 5 electrodes for both graphene/PEDOT:PSS and Pt electrodes. For heterogeneous co-culture experiment, two biological replicates were analyzed, both exhibiting a similar distribution of CM states and surrounding cell types (Supplementary Fig. 12). The primary cell niches identified near electrodes differed between samples, likely due to variation in electrode positioning within the heterogeneous co-culture and the extent of hiPSC-EC diffusion into the hiPSC-CM-only region. The samples were therefore considered comparable for subsequent analyses. For cardiac organoid recordings, a single sample was recorded five times at different time points. For the 5-bit barcode in situ sequencing demonstration, one sample was processed.

Data exclusion, randomization, and blinding: Waveforms lacking discernible FPD peaks were excluded from FPD analyses. During spatial transcriptomic data processing, cells with fewer than five total reads and genes expressed in fewer than five cells were excluded to remove potential cell debris, under-segmented cells, and genes with low mapping quality. The experiments were not randomized, as experimental conditions were predefined by the engineered platform. Investigators were not blinded to allocation during experiments and outcome assessment.

Statistical analysis: Statistical analyses were performed using GraphPad Prism. Data normality was assessed with the Shapiro-Wilk test, and appropriate nonparametric or parametric tests were applied accordingly. Details of the specific statistical tests are provided in the figure legends.

### Reporting summary

Further information on research design is available in the Nature Portfolio Reporting Summary linked to this article.

## Supplementary information

Supplementary Information

Description of Additional Supplementary Files

Supplementary Data 1

Supplementary Data 2

Reporting Summary

Transparent Peer Review file

## Source data

Source Data

## Data Availability

Imaging data underlying the in situ sequencing experiments have been deposited in the BioImage Archive under accession number S-BIAD3370. Single-cell sequencing data used for data integration are available from the NCBI Gene Expression Omnibus (GEO) under accession code GSE210513. Processed datasets used for analysis and figure generation are available on GitHub (http://github.com/LiuLab-Bioelectronics-Harvard/Graphene_seq) and archived on Zenodo (10.5281/zenodo.19520229). Source data are provided with this paper.

## Code availability

The code used to perform the analyses and generate results in this study is publicly available and has been deposited in a GitHub repository at (http://github.com/LiuLab-Bioelectronics-Harvard/Graphene_seq), under the Apache-2.0 license. The specific version of the code associated with this publication is archived in Zenodo and is accessible via 10.5281/zenodo.19520229^63^.
