# Supplementary material for: In situ graphene-seq: spatial transcriptomics and chronic electrophysiological characterization of tissue microenvironments
Source: Nat Commun. 2026 Jun 19;17:8382. doi: 10.1038/s41467-026-73883-7 (PMC13473619; doi:10.1038/s41467-026-73883-7)
Supplement: Supplementary file 1 [file 41467_2026_73883_MOESM1_ESM.pdf]

Supplementary Information

In situ graphene-seq: spatial transcriptomics and chronic electrophysiological characterization of tissue microenvironments

Jaeyong Lee[1†], Wenbo Wang[1,2†], Qiang Li[1], Zuwan Lin[1,2], Ren Liu[1], Zefang Tang[2], Junya Aoyama[3], Richard T. Lee[3,4], Xiao Wang[2,5], Jia Liu[1*]

[1] John A. Paulson School of Engineering and Applied Sciences, Harvard University, Boston, MA, USA

[2] Broad Institute of MIT and Harvard, Cambridge, MA, USA

[3] Department of Stem Cell and Regenerative Biology, Harvard University, Cambridge, MA, USA

[4] Division of Cardiovascular Medicine, Department of Medicine, Brigham and Women's Hospital and Harvard Medical School, Boston, MA, USA

[5] Department of Chemistry, Massachusetts Institute of Technology, Cambridge, MA, USA

[†]These authors contributed equally: Jaeyong Lee, Wenbo Wang

[*]Email: jia_liu@seas.harvard.edu

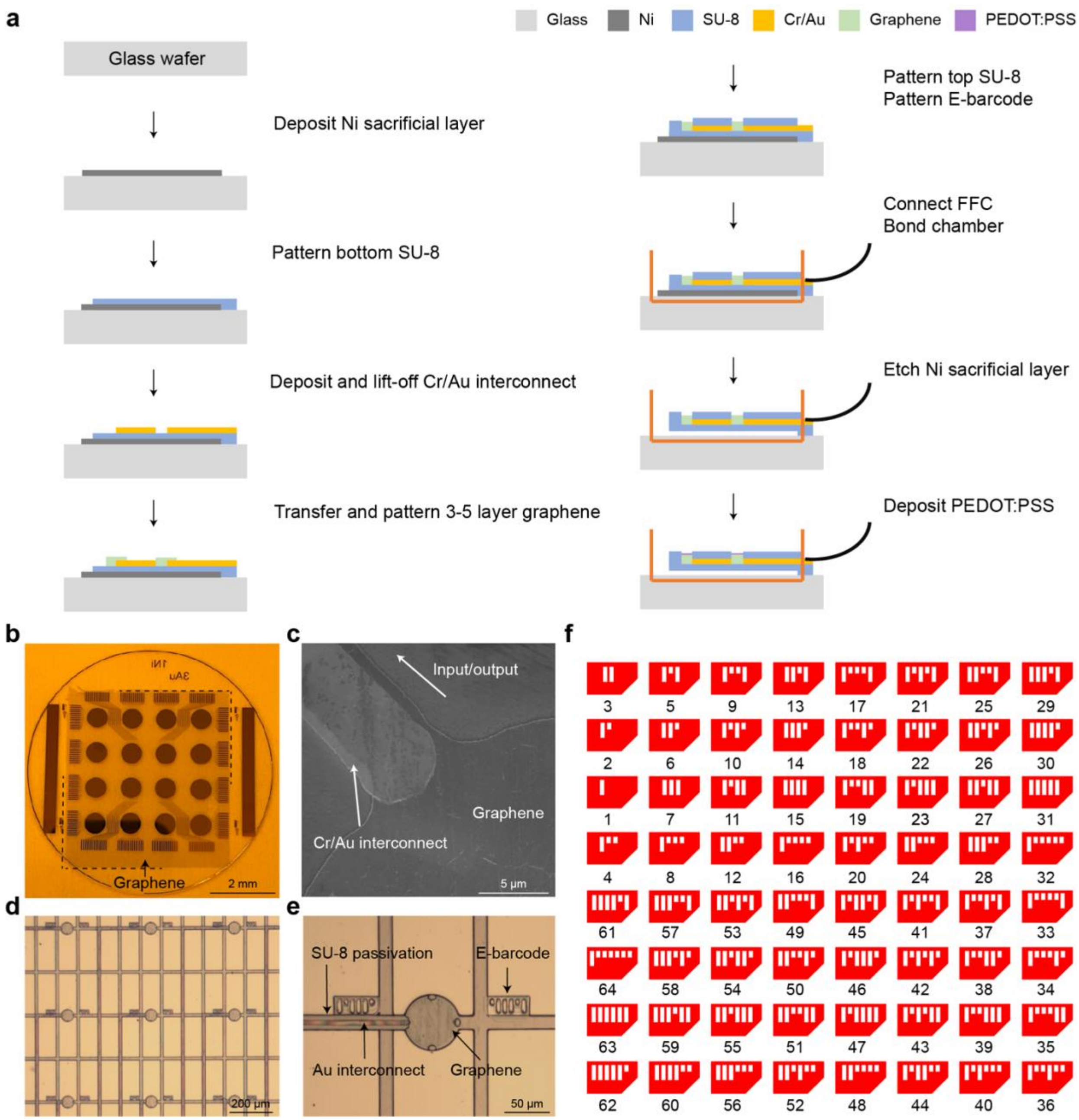

Supplementary Fig. 1: Fabrication of stretchable mesh nanoelectronics with transparent electrodes. **a**, Schematic overview of the fabrication process (Ni, nickel; Cr, chromium; Au, gold; FFC, flexible flat cable; PEDOT:PSS, poly(3,4-ethylenedioxythiophene) polystyrene sulfonate). **b**, Photograph of graphene transferred onto devices fabricated on a 3-inch glass wafer. **c**, Scanning electron microscopy (SEM) image of graphene on metal interconnects before top SU-8 passivation. Graphene covers part of interconnects, forming an electrically conductive path to input/output pads. **d**, Optical image of a representative mesh electronics array. **e**, Optical image of a representative graphene electrode with lithographically patterned fluorescent E-barcodes. **f**, Design layout of E-barcodes positioned laterally adjacent to electrodes. The barcode geometry and footprint can be adapted through alternative encoding strategies and lithographic parameters.

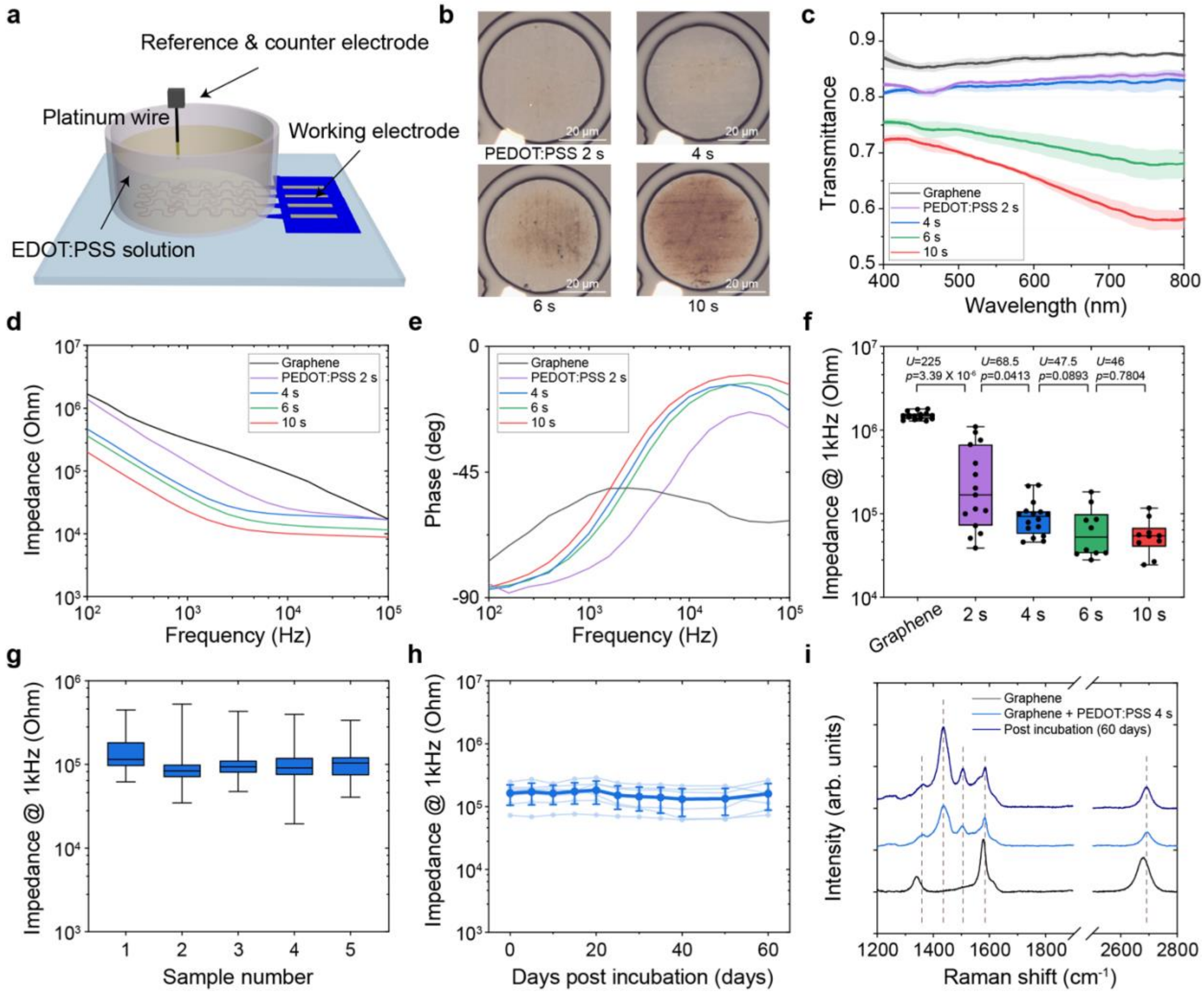

Supplementary Fig. 2: Electrochemical deposition of PEDOT:PSS on graphene electrodes. **a**, Schematic diagram of the setup used for PEDOT:PSS electrochemical deposition. **b**, Optical images of representative graphene electrodes deposited with PEDOT:PSS at varying deposition times. **c**, Transmittance spectra of graphene/PEDOT:PSS electrodes from 400 to 800 nm. Data are presented as mean ± S.D. (n=5 electrodes per condition). **d–e**, Amplitude (**d**) and phase angle (**e**) of electrochemical impedance spectra measured from representative graphene/PEDOT:PSS electrodes across frequencies ranging from 0.1 to 100 kHz. **f**, Electrochemical impedance at 1 kHz of graphene/PEDOT:PSS electrodes with varying deposition time (n=15, 15, 16, 10, and 10 electrodes per condition). Each dot represents an individual electrode. Statistical significance was assessed using a two-tailed Mann-Whitney U test. **g**, Box-and-whisker plot showing electrochemical impedance at 1 kHz across electrodes from five devices (n=60, 62, 63, 63, and 56 electrodes per device). **h**, Impedance stability at 1 kHz over time for seven representative electrodes incubated in 1X PBS at 37 °C. Data are presented as mean ± S.D. **i**, Raman spectra of representative graphene and graphene/PEDOT:PSS electrodes before and after 60 days of incubation in 1X PBS at 37 °C. For all box-and-whisker plots, center lines indicate the median, box bounds represent the 25th–75th percentiles, and whiskers denote the minima and maxima. Source data are provided as a Source Data file.

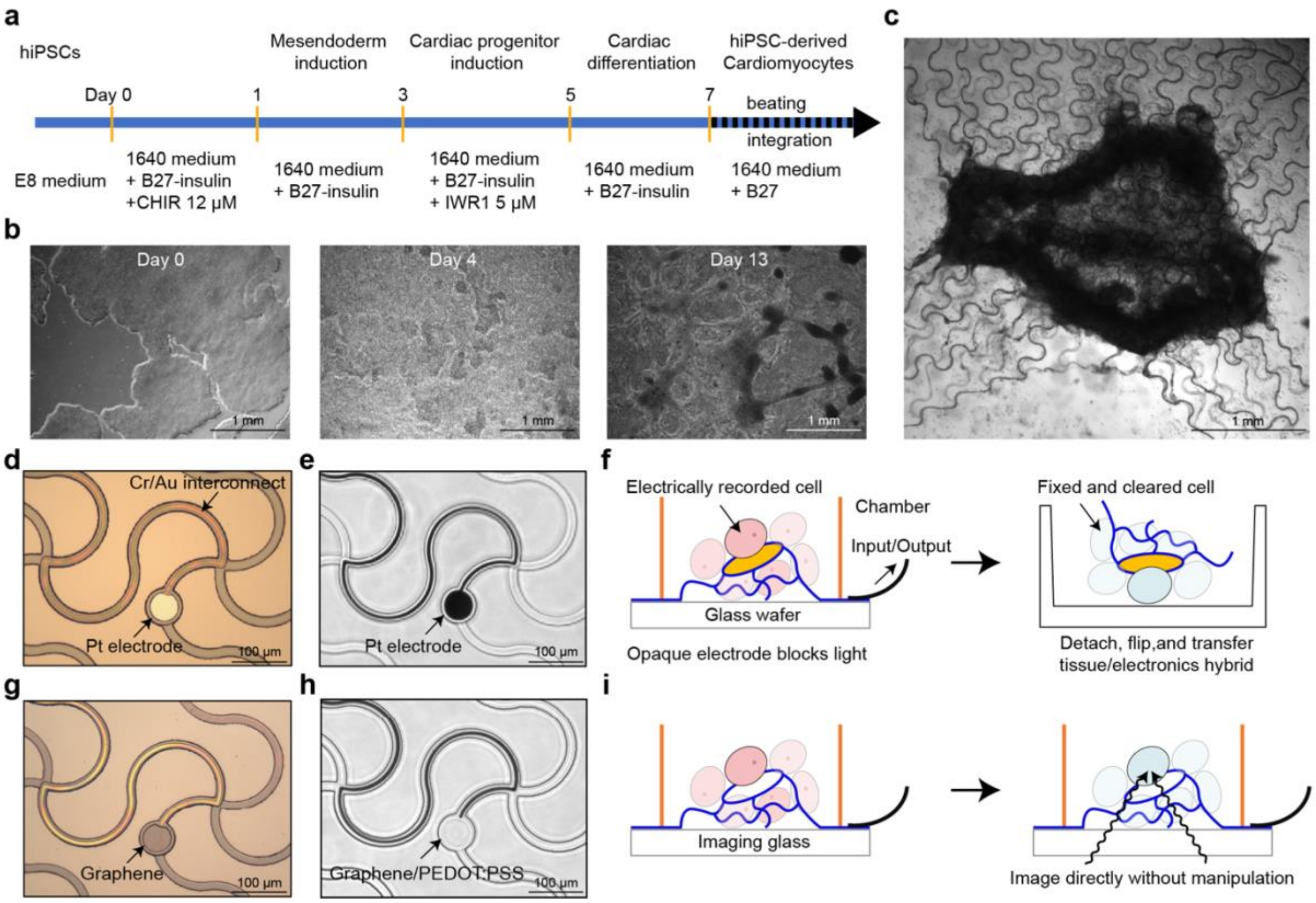

Supplementary Fig. 3: Differentiation and integration of human induced pluripotent stem cell-derived cardiomyocytes (hiPSC-CMs) with stretchable and transparent mesh nanoelectronics. a, Schematic illustrating the protocol for differentiating cardiomyocytes from hiPSCs. **b**, Bright-field (BF) images showing cells at different stages of the differentiation process. **c**, BF image of a 3D cardiac organoid integrated with mesh nanoelectronics. **d–e**, Optical images of conventional mesh electronics with metal electrodes before (**d**) and after (**e**) release from the substrate. **f**, Schematic illustrating the additional experimental steps required to image cells on top of opaque metal electrodes. **g–h**, Optical images of graphene electrodes before (**g**) and after (**h**) release from the substrate and PEDOT:PSS deposition. **i**, Schematic illustrating how transparent electrodes enable direct imaging of electrically recorded cells.

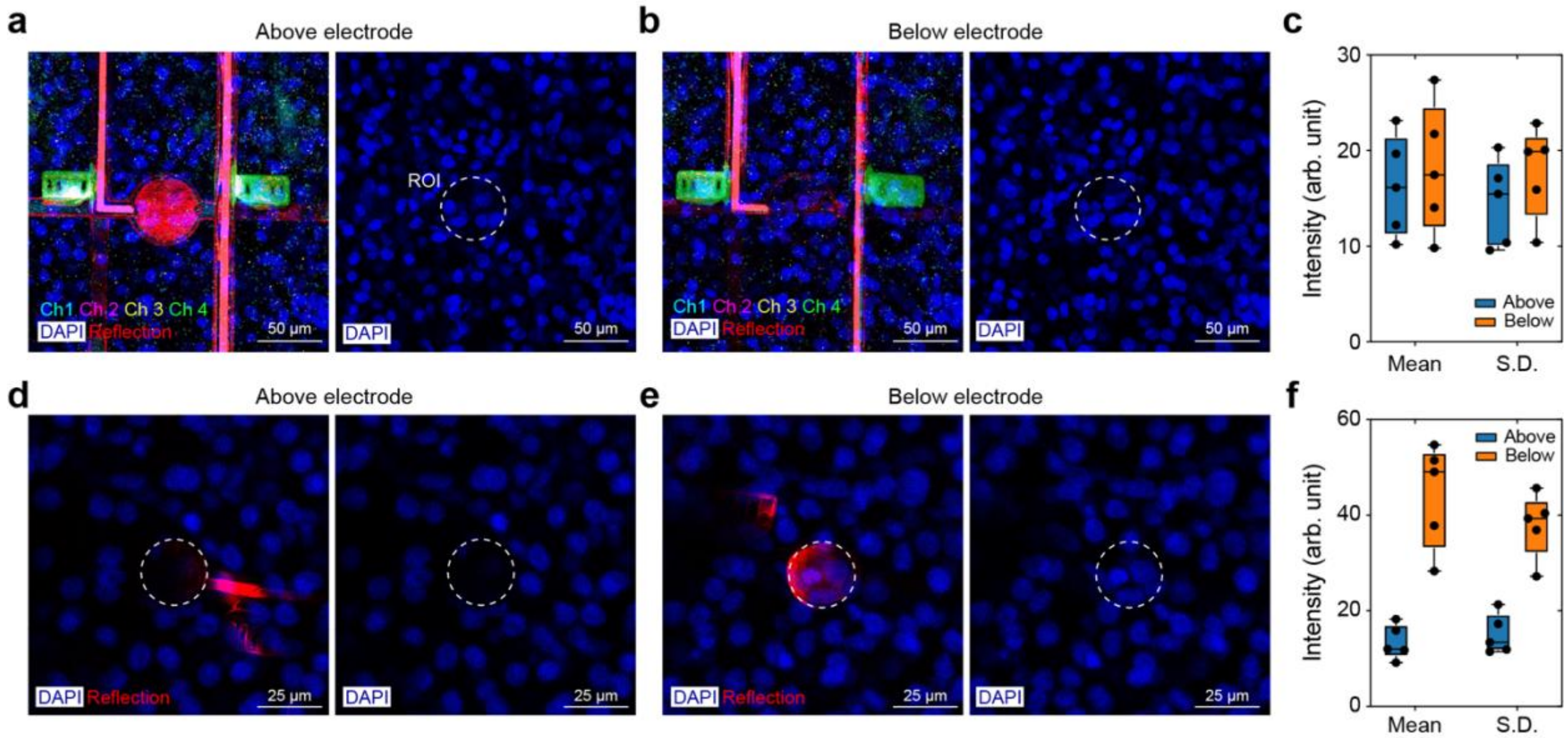

Supplementary Fig. 4: Optical accessibility enabled by transparent electrodes. **a–b**, Representative confocal images of cells integrated with transparent electrode arrays. Left panels show composite views, including reflection mode (red) to verify electrode location relative to the region of interest (ROI). Right panels show the corresponding DAPI channel used for analysis. **a**, Cells above the electrode. **b**, Cells below the electrode. **c**, Quantification of DAPI pixel intensity within the same ROI for regions above and below the electrode (*n*=5 electrodes). **d–e**, Representative confocal images of cells integrated with metal electrode arrays, displayed as in (a–b). **f**, Quantification of DAPI pixel intensity within the same ROI for regions above and below the electrode (*n*=5 electrodes). Source data are provided as a Source Data file.

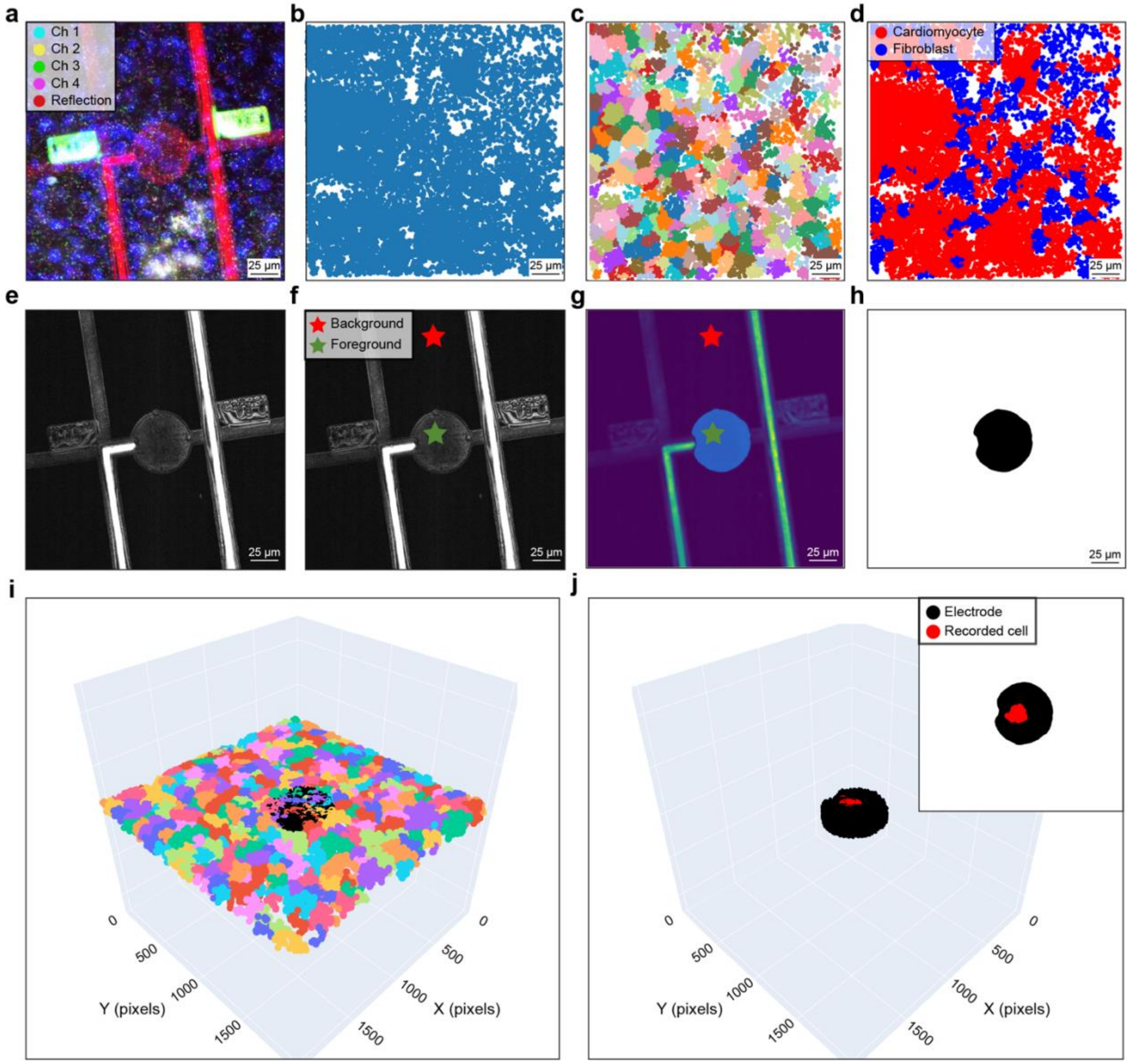

Supplementary Fig. 5: Image processing pipeline for in situ sequencing data. a–d, In situ sequencing data processing workflow. **a**, Representative raw complementary DNA (cDNA) amplicons (channels 1–4) showing fluorescence barcodes (green) and the device in reflection mode (red). **b**, Registered cDNA amplicons. **c**, Segmented cells. **d**, Assigned cell types mapped to segmented cells. **e–h**, Graphene/PEDOT:PSS electrode segmentation pipeline. **e**, Maximum-intensity projection of the device in reflection mode. **f**, Segmentation of background and foreground regions to identify electrodes. **g**, Generated electrode mask. **h**, Binary mask of segmented electrodes for downstream analysis. **i–j**, Identification of electrically recorded cells. **i**, 3D reconstruction of electrodes with cells color-coded by segmentation. **j**, Identification of electrically recorded cells positioned over the electrode.

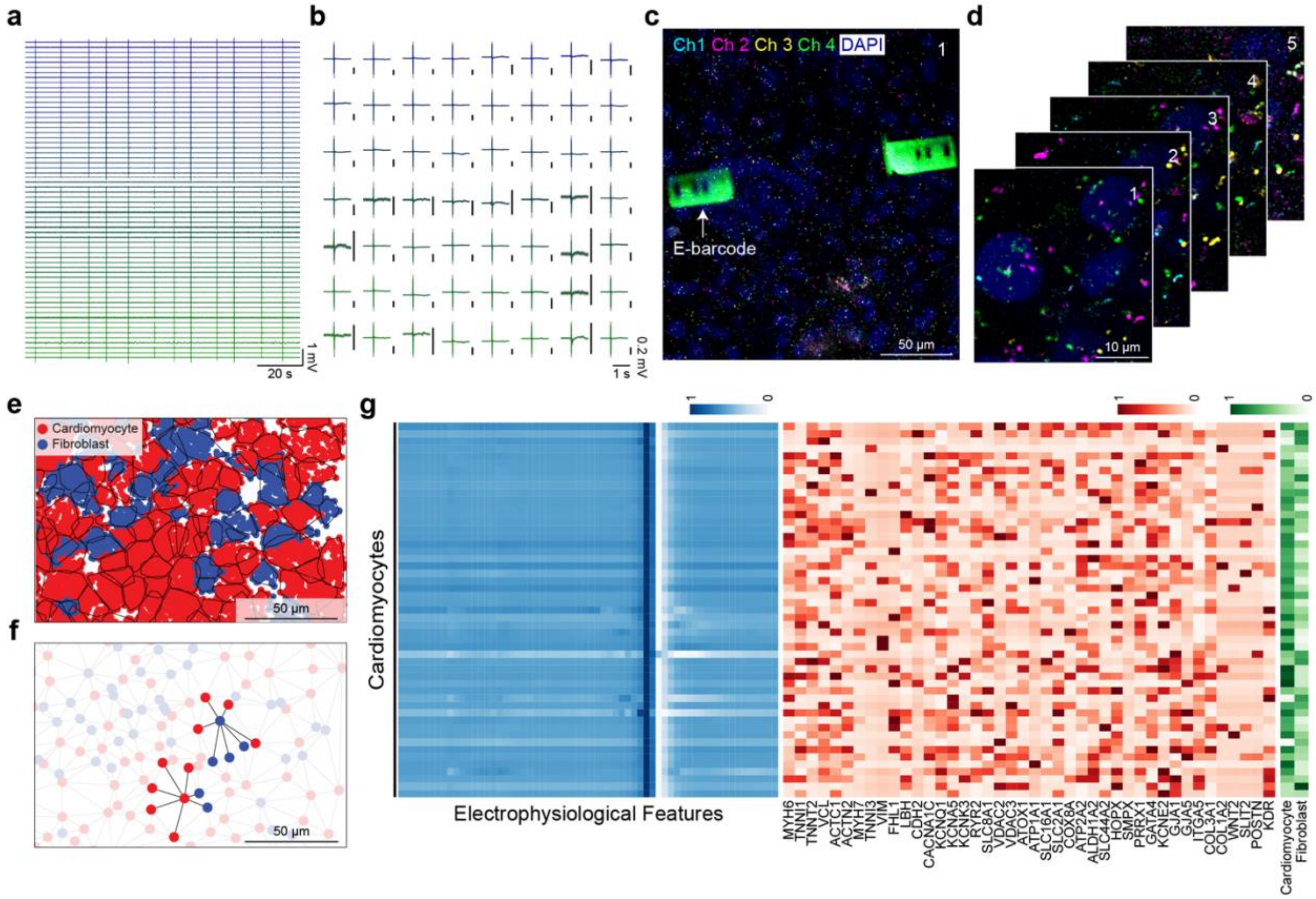

Supplementary Fig. 6: In situ graphene-seq of homogeneous hiPSC-derived cardiac microtissue. a, Representative voltage traces recorded from 64 electrode arrays. **b**, Representative spike waveforms (mean ± S.D.) corresponding to the recordings in (a). **c**, Representative in situ sequencing image showing fluorescence channels and E-barcodes. **d**, Zoom-in view of five sequencing cycles. **e**, Cell-type map showing the spatial distribution of different types of cells within the microtissue. **f**, Cell adjacency networks of representative cardiomyocyte and fibroblast, illustrating spatial proximity and cellular interactions. **g**, Concatenated multimodal heatmaps of electrophysiological features, gene expression profiles, and local cell density of electrically recorded cardiomyocytes across the microtissue.

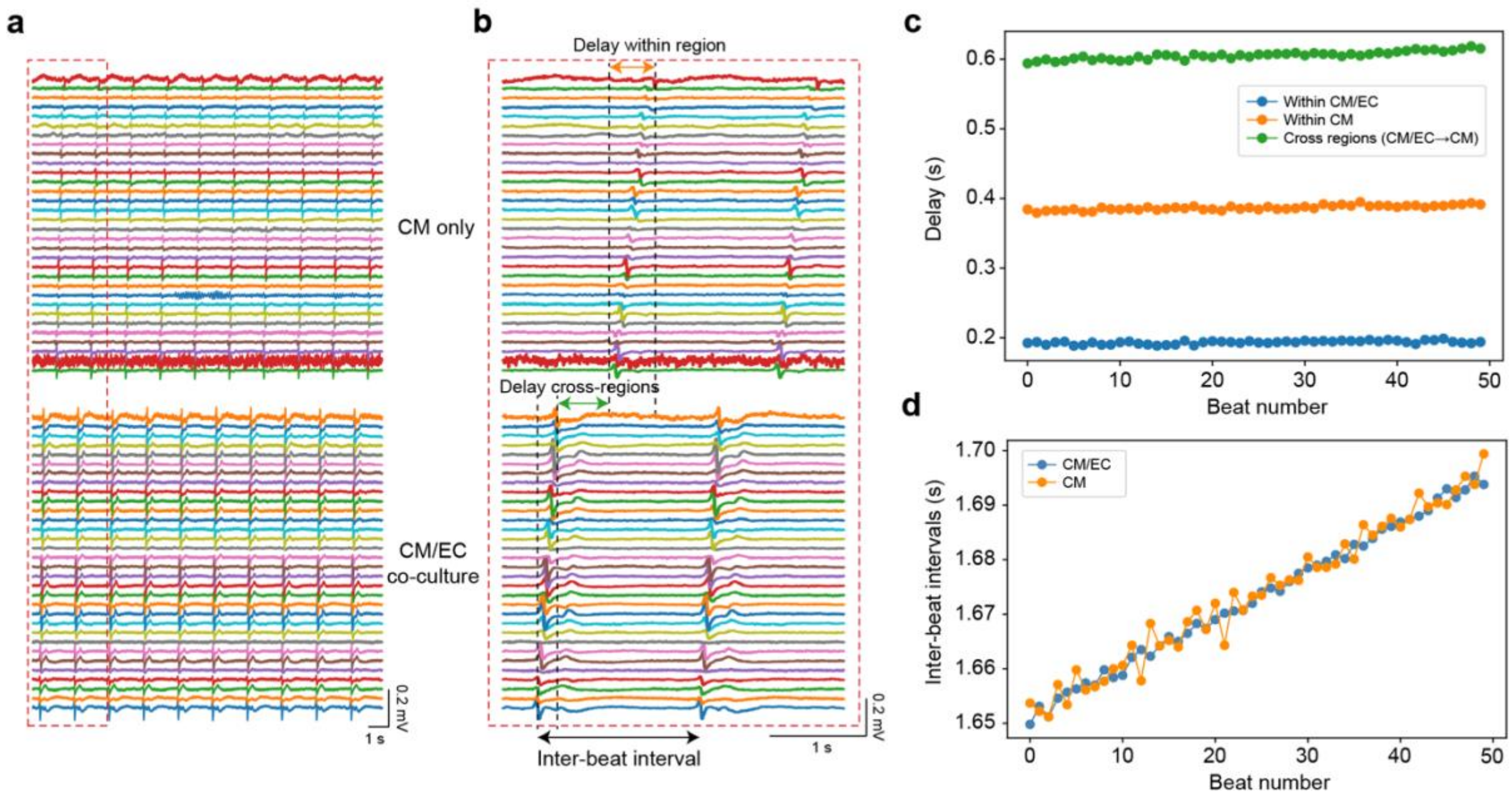

Supplementary Fig. 7: Beating synchrony and conduction delay between CM/EC and CM regions. a, Representative voltage traces showing rhythmic depolarizations recorded from CM/EC (hiPSC-derived cardiomyocyte/endothelial cell co-culture) and CM (hiPSC-derived cardiomyocyte-only) regions. **b**, Zoom-in view of two consecutive beats with annotated measurement criteria. **c**, Beat-by-beat conduction delays within the CM/EC region (blue), within the CM region (orange), and across the two regions (green). The cross-region delay (CM/EC → CM) remained stable at ~0.6 s, while within-region delays were shorter (0.19 s and 0.39 s, respectively). **d**, Beat-to-beat inter-beat intervals of CM/EC and CM regions showing identical temporal trends with a gradual, parallel increase over time, indicating a shared rhythm and electrical coupling between the regions.

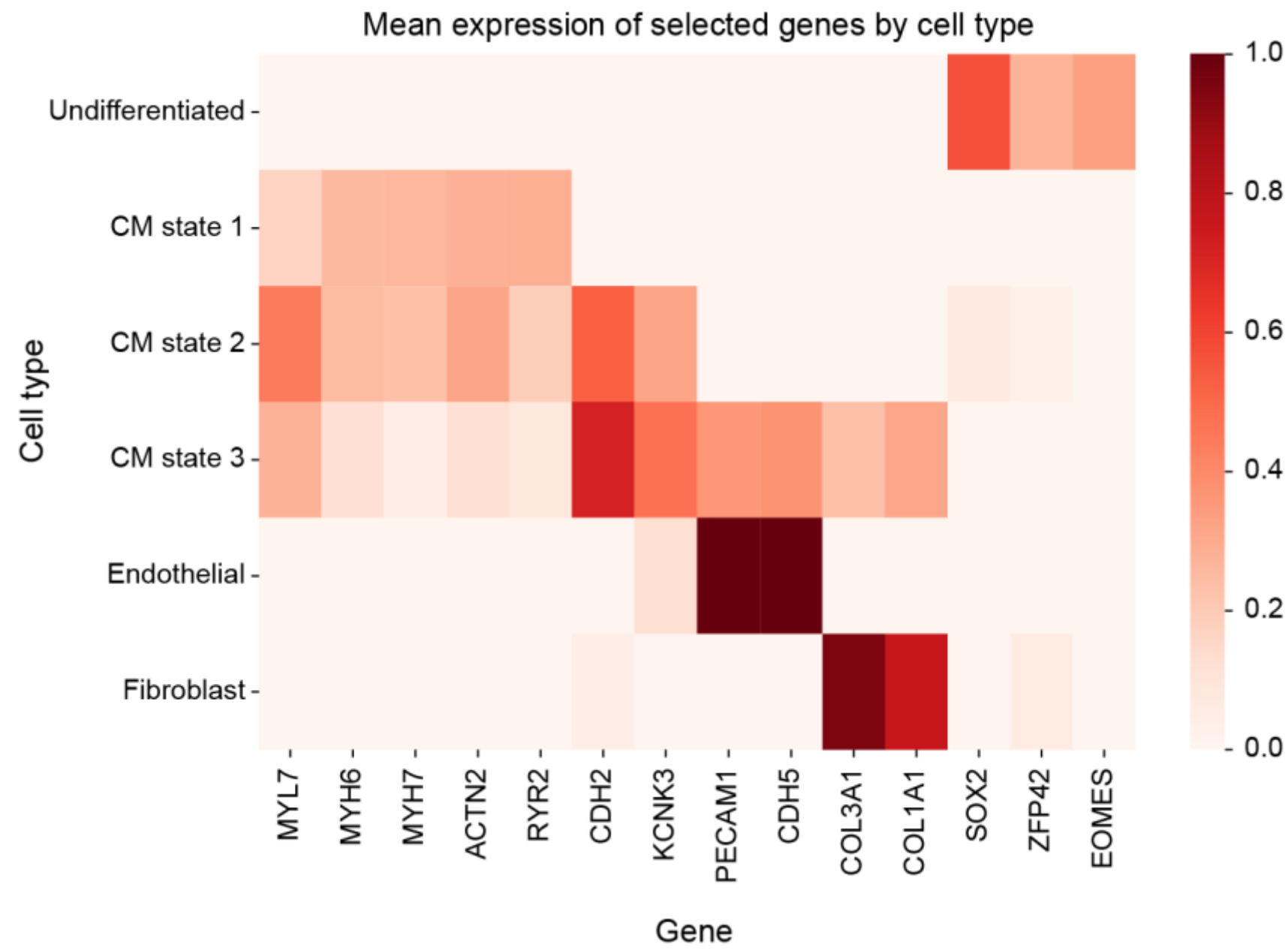

Supplementary Fig. 8: Marker gene expression. Mean expression profiles of key marker genes across cell types in the hiPSC-derived cardiomyocyte/endothelial cell (CM/EC) heterogeneous co-culture system.

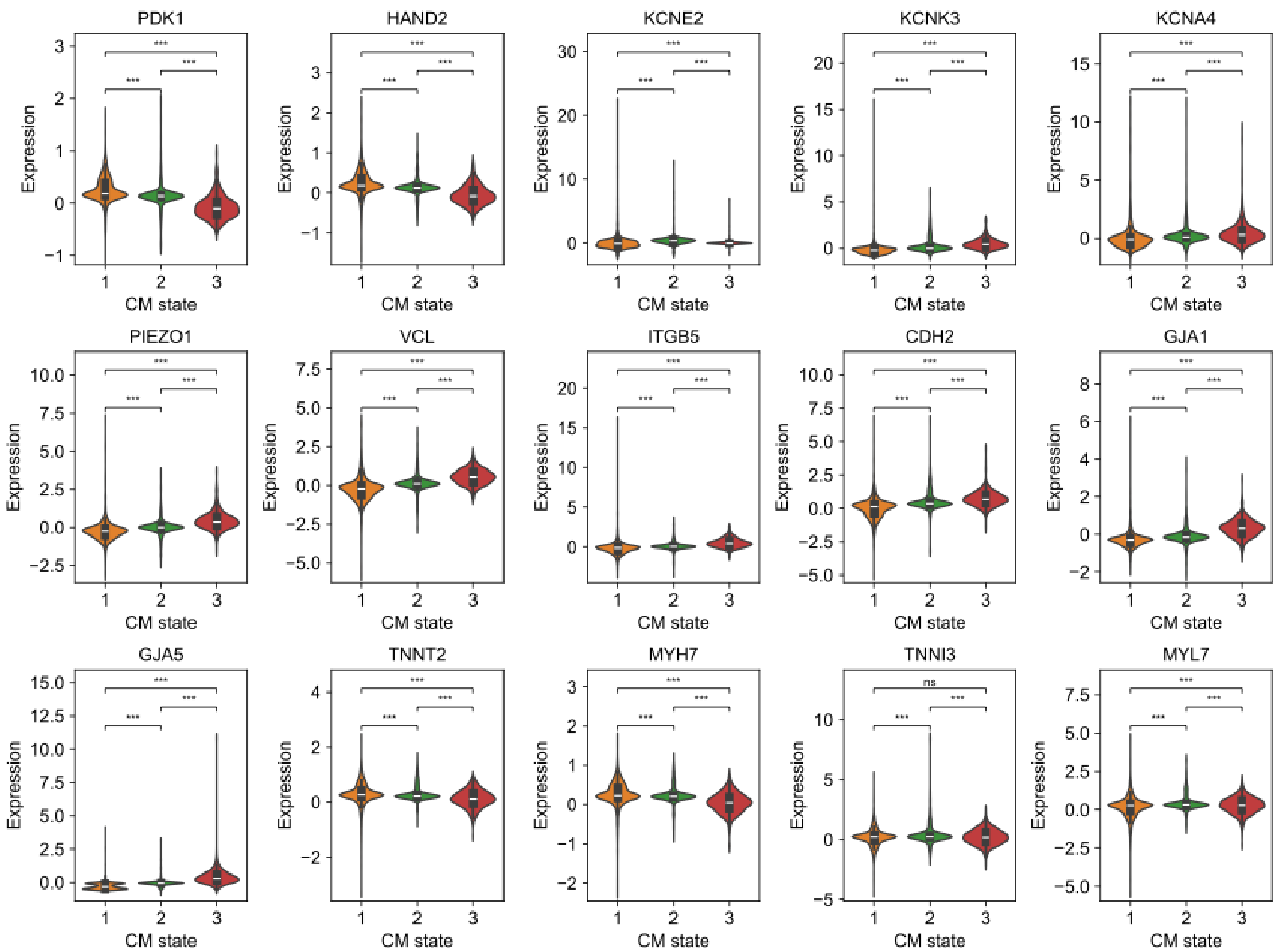

Supplementary Fig. 9: Gene expression profiles of different hiPSC-derived cardiomyocyte states. Violin plots illustrating the expression levels of selected genes across cardiomyocyte (CM) states. Statistical significance was assessed using two-tailed Mann-Whitney *U* tests for all pairwise comparisons between CM states (* $p<0.05$, ** $p<0.01$, *** $p<0.001$, ns: not significant). Sample sizes: CM state 1 (n=1,700), CM state 2 (n=643), and CM state 3 (n=493) cells. Exact *p*-values are provided in the Source Data file.

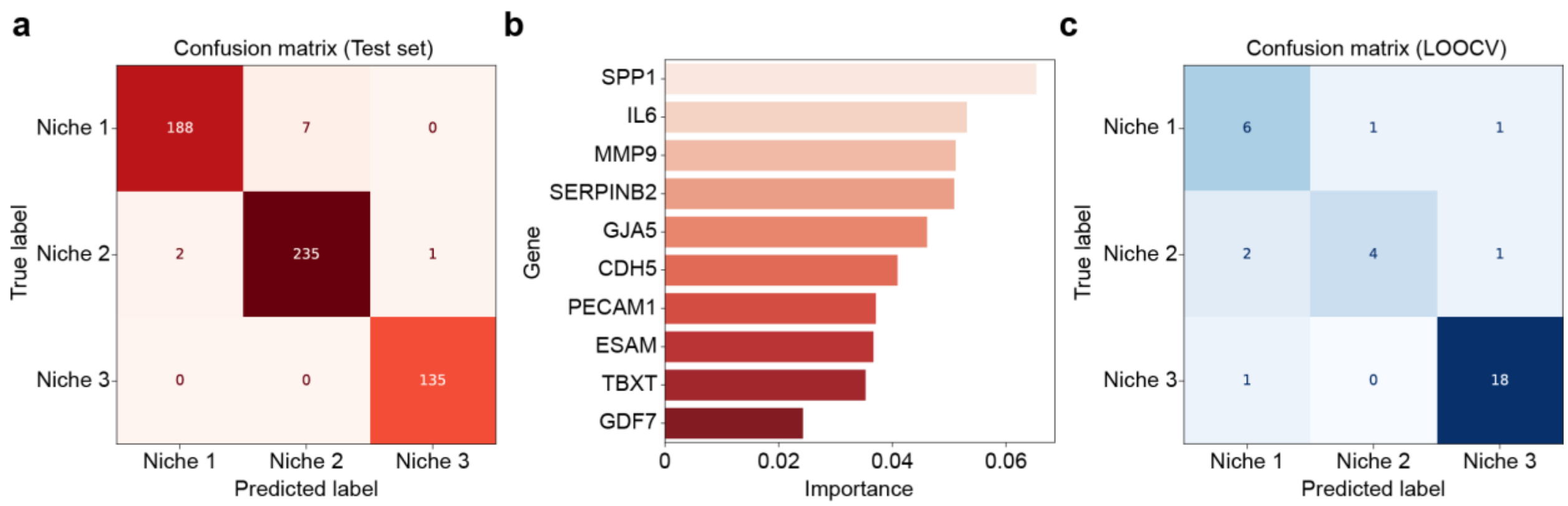

Supplementary Fig. 10: Predictive inference of spatial niches from molecular and electrophysiological features. a, Confusion matrix of a random forest classifier trained on gene expression data to predict spatial niche identity of cardiomyocytes, evaluated on a held-out test set (80/20 split). **b**, Ranked feature importance of the top genes contributing to niche prediction. **c**, Confusion matrix of a random forest classifier trained on electrophysiological features, evaluated using leave-one-out cross-validation (LOOCV).

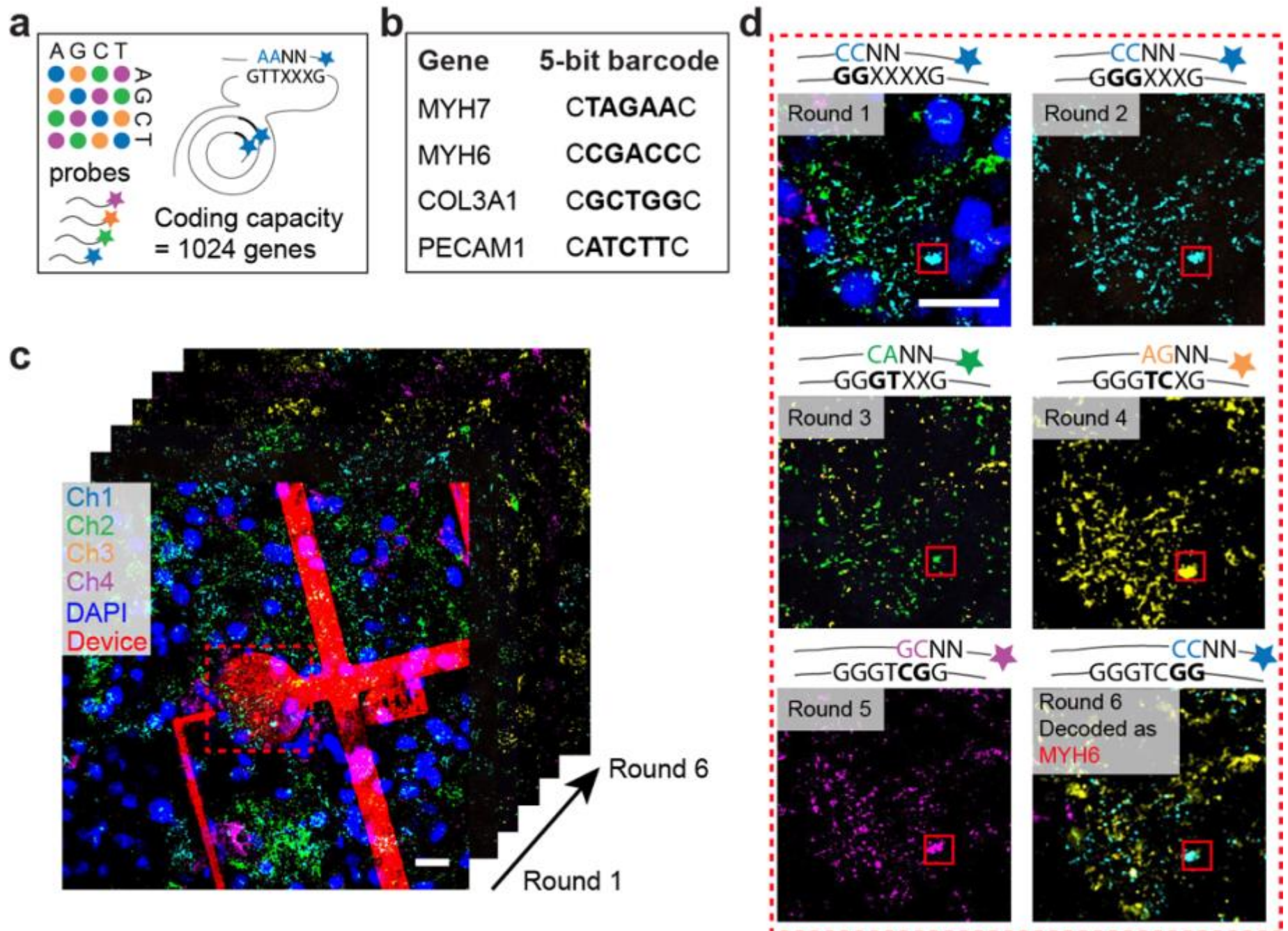

Supplementary Fig. 11: In situ graphene-seq with 5-bit gene barcodes. **a**, Schematic of the decoding process using 5-bit barcodes, including degenerate bases at both the 5' and 3' ends. **b**, Gene panel and barcode design for 5-bit in situ graphene-seq. **c**, Representative images from six sequencing cycles. The red dashed box indicates the graphene/PEDOT:PSS electrode region. **d**, Zoom-in view of the boxed region in (c), with the device channel omitted to highlight amplicons. Scale bar: 20 µm for panels (c, d). Amplicons were clearly resolved directly over electrodes across all six cycles, with high signal-to-noise ratio and no detectable interference with fluorescence imaging or sequential ligation chemistry. These results demonstrate compatibility of in situ graphene-seq with multi-round imaging workflows required for large-scale spatial transcriptomic panels.

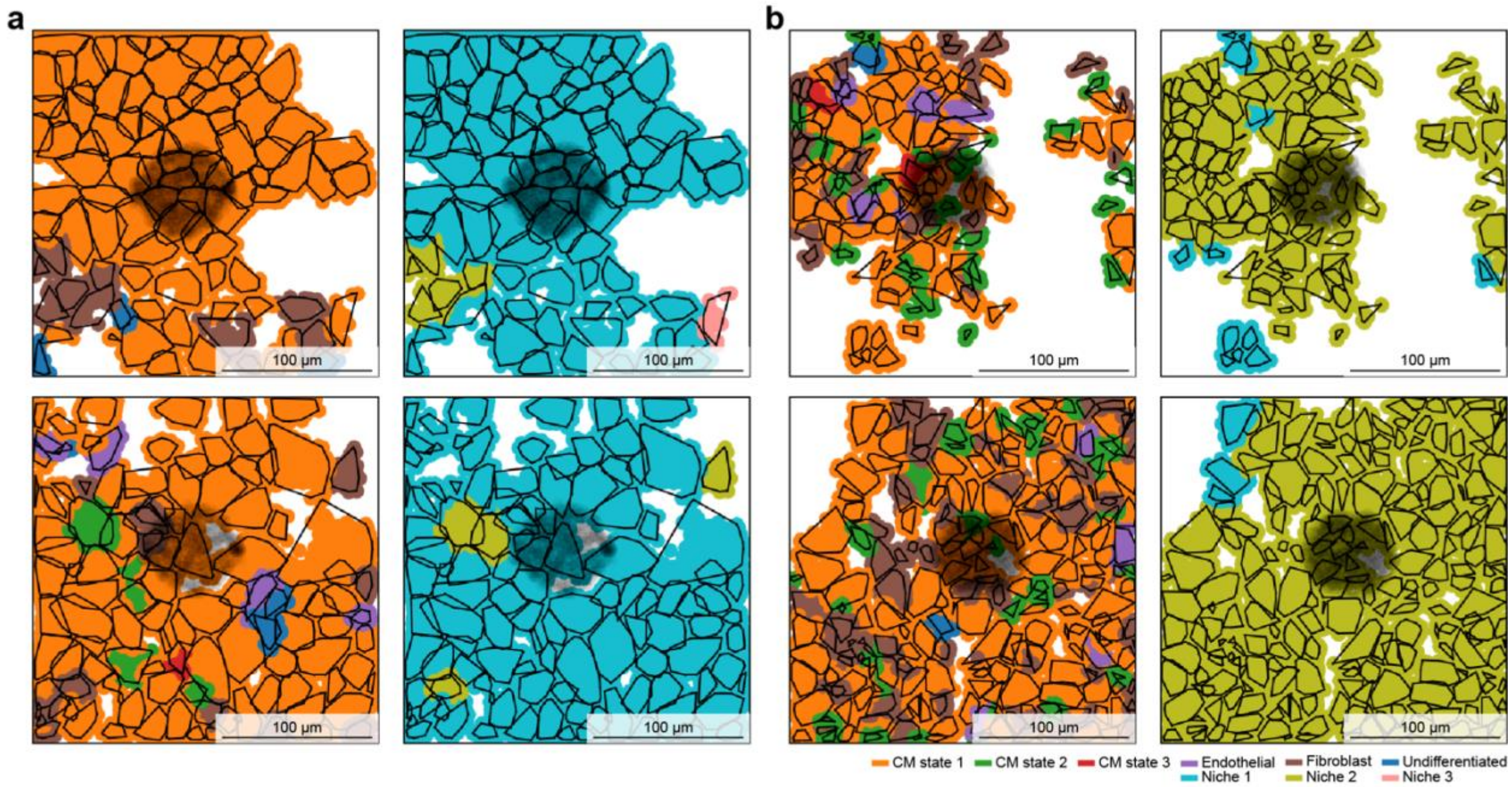

Supplementary Fig. 12: Sample information. a–b, Representative cell-type maps (left) and niche-type maps (right) of cardiomyocyte-only regions in hiPSC-derived cardiomyocyte/endothelial cell (CM/EC) heterogeneous co-culture samples, with electrode locations overlaid. **a**, Sample 1. **b**, Sample 2.
