# Supplementary material for: In situ graphene-seq: spatial transcriptomics and chronic electrophysiological characterization of tissue microenvironments
Source: Nat Commun. 2026 Jun 19;17:8382. doi: 10.1038/s41467-026-73883-7 (PMC13473619; doi:10.1038/s41467-026-73883-7)
Supplement: Supplementary file 2 [file 41467_2026_73883_MOESM2_ESM.pdf]

Description of Additional Supplementary Files:

Supplementary Data 1: In situ sequencing probe sequences for the 254-gene panel.

Supplementary Data 2: In situ sequencing probe sequences for the 5-bit barcode demonstration.
