# Supplementary material for: In situ graphene-seq: spatial transcriptomics and chronic electrophysiological characterization of tissue microenvironments
Source: Nat Commun. 2026 Jun 19;17:8382. doi: 10.1038/s41467-026-73883-7 (PMC13473619; doi:10.1038/s41467-026-73883-7)
Supplement: Supplementary file 3 [file 41467_2026_73883_MOESM5_ESM.pdf]

nature portfolio

Corresponding author(s): Jia Liu

Last updated by author(s): May 6, 2026

Reporting Summary

Nature Portfolio wishes to improve the reproducibility of the work that we publish. This form provides structure for consistency and transparency in reporting. For further information on Nature Portfolio policies, see our Editorial Policies and the Editorial Policy Checklist.

Statistics

For all statistical analyses, confirm that the following items are present in the figure legend, table legend, main text, or Methods section.

n/a	Confirmed	
☐	☒	The exact sample size (*n*) for each experimental group/condition, given as a discrete number and unit of measurement
☐	☒	A statement on whether measurements were taken from distinct samples or whether the same sample was measured repeatedly
☐	☒	The statistical test(s) used AND whether they are one- or two-sided<br>*Only common tests should be described solely by name; describe more complex techniques in the Methods section.*
☒	☐	A description of all covariates tested
☐	☒	A description of any assumptions or corrections, such as tests of normality and adjustment for multiple comparisons
☐	☒	A full description of the statistical parameters including central tendency (e.g. means) or other basic estimates (e.g. regression coefficient) AND variation (e.g. standard deviation) or associated estimates of uncertainty (e.g. confidence intervals)
☐	☒	For null hypothesis testing, the test statistic (e.g. *F*, *t*, *r*) with confidence intervals, effect sizes, degrees of freedom and *P* value noted<br>*Give P values as exact values whenever suitable.*
☒	☐	For Bayesian analysis, information on the choice of priors and Markov chain Monte Carlo settings
☒	☐	For hierarchical and complex designs, identification of the appropriate level for tests and full reporting of outcomes
☒	☐	Estimates of effect sizes (e.g. Cohen's *d*, Pearson's *r*), indicating how they were calculated

Our web collection on statistics for biologists contains articles on many of the points above.

Software and code

Policy information about availability of computer code

Data collection	No software was used for data collection.
Data analysis	Data analysis and visualization were performed using MATLAB R2022b and Python 3.8, with the following packages: Numpy 1.24.3, pandas 2.0.3, matplotlib 3.7.5, seaborn 0.13.2, SciPy 1.10.1, Scanpy 1.9.8, anndata 0.9.2, Tangram 0.4.0, SAM (Segment Anything Model; https://github.com/facebookresearch/segment-anything), Leiden clustering(via leidenalg 0.10.2), UMAP 0.5.5, scikit-learn 1.3.2, SpikeInterface 0.98.2, and PyWavelets 1.4.1. For pseudotime analysis, rpy2 3.5.16 was used to interface with the R package slingshot (https://github.com/kstreet13/slingshot). Statistical analyses were conducted using Prism 10.4.1. Custom code used in this study is available on Github (https://github.com/LiuLab-Bioelectronics-Harvard/Graphene_seq) under the Apache-2.0 license. The specific version of the code associated with this publication is archived on Zenodo and is accessible via https://doi.org/10.5281/zenodo.19520229

For manuscripts utilizing custom algorithms or software that are central to the research but not yet described in published literature, software must be made available to editors and reviewers. We strongly encourage code deposition in a community repository (e.g. GitHub). See the Nature Portfolio guidelines for submitting code & software for further information.

Data

Policy information about availability of data

All manuscripts must include a data availability statement. This statement should provide the following information, where applicable:

- Accession codes, unique identifiers, or web links for publicly available datasets
- A description of any restrictions on data availability
- For clinical datasets or third party data, please ensure that the statement adheres to our policy

Imaging data underlying the in situ sequencing experiments have been deposited in the BioImage Archive under accession number S-BIAD3370. Single-cell sequencing data used for data integration are available from the NCBI Gene Expression Omnibus (GEO) under accession code GSE210513. Processed datasets used for analysis and figure generation are available on GitHub (http://github.com/LiuLab-Bioelectronics-Harvard/Graphene_seq) and archived on Zenodo (https://doi.org/10.5281/zenodo.19520229). Source data are provided with this paper.

Research involving human participants, their data, or biological material

Policy information about studies with human participants or human data. See also policy information about sex, gender (identity/presentation), and sexual orientation and race, ethnicity and racism.

Reporting on sex and gender	N/A
Reporting on race, ethnicity, or other socially relevant groupings	N/A
Population characteristics	N/A
Recruitment	N/A
Ethics oversight	N/A

Note that full information on the approval of the study protocol must also be provided in the manuscript.

Field-specific reporting

Please select the one below that is the best fit for your research. If you are not sure, read the appropriate sections before making your selection.

☒ Life sciences ☐ Behavioural & social sciences ☐ Ecological, evolutionary & environmental sciences

For a reference copy of the document with all sections, see nature.com/documents/nr-reporting-summary-flat.pdf

Life sciences study design

All studies must disclose on these points even when the disclosure is negative.

Sample size	No statistical method was used to predetermine sample size. Sample sizes were determined based on experimental feasibility, including the capacity of the electronic platforms and the yield of viable tissue/electronics interfaces, and were sufficient to support the conclusions drawn in this study. For in situ sequencing of CM-only and CM/EC co-culture experiments, two devices were processed. For cardiac organoid recordings, a single sample was recorded five times at different time points. For 5-bit barcode in situ sequencing experiments, one sample was processed.
Data exclusions	Waveforms lacking discernable field potential duration (FPD) peaks were excluded from FPD analyses. During spatial transcriptomic data processing, cells with fewer than five total reads and genes expressed in fewer than five cells were excluded to remove potential cell debris, under-segmented cells, and genes with low mapping quality.
Replication	All major findings were validated in the second biological replicate.
Randomization	Within each experimental batch, CM samples were randomly selected based on differentiation time, and ECs (maintained as a stable cell line) were selected based on confluency. To ensure consistency, CM-only and CM/EC co-culture Graphene-seq experiments were performed using CMs from the same differentiation batch. Experiments were not randomized, as experimental conditions were predefined by the engineered platform.
Blinding	Investigators were not blinded to allocation during experiments and outcome assessment.

Reporting for specific materials, systems and methods

We require information from authors about some types of materials, experimental systems and methods used in many studies. Here, indicate whether each material, system or method listed is relevant to your study. If you are not sure if a list item applies to your research, read the appropriate section before selecting a response.

Materials & experimental systems

n/a	Involved in the study
☐	☒ Antibodies
☐	☒ Eukaryotic cell lines
☒	☐ Palaeontology and archaeology
☒	☐ Animals and other organisms
☒	☐ Clinical data
☒	☐ Dual use research of concern
☒	☐ Plants

Methods

n/a	Involved in the study
☒	☐ ChIP-seq
☒	☐ Flow cytometry
☒	☐ MRI-based neuroimaging

Antibodies

Antibodies used: The following antibodies were used for immunohistochemistry. Primary antibodies were used at a dilution of 1:200: Mouse Cardiac Troponin T Monoclonal Antibody (13-11) (MA5-12960, Invitrogen), Rabbit Anti-Nkx2.5 antibody (ab97355, abcam), Rabbit CD31/ PECAM-1 Antibody - BSA Free (NBP1-71663, Novus Biologicals). Secondary antibodies were used at a dilution of 1:600: Goat anti-Rabbit IgG (H+L) Highly Cross-Adsorbed Secondary Antibody, Alexa Fluor™ 647 (A-21245, Invitrogen), and Goat anti-Mouse IgG (H+L) Cross-Adsorbed Secondary Antibody, Alexa Fluor™ 594 (A-11005, Invitrogen).

Validation: Antibody validation was based on manufacturer-provided datasheets and prior literature where applicable. All antibodies were used according to the manufacturers' recommended applications.

Eukaryotic cell lines

Policy information about cell lines and Sex and Gender in Research

Cell line source(s): Human induced pluripotent stem cells (hiPSCs; IMR-90-1, female donor) were obtained from the WiCell Research Institute (Madison, WI, USA).

Authentication: Authentication was performed by the vendor through Short Tandem Repeat (STR).

Mycoplasma contamination: Mycoplasma testing were performed by the vendor.

Commonly misidentified lines (See ICLAC register): None

Plants

Seed stocks: N/A

Novel plant genotypes: N/A

Authentication: N/A
