# Supplementary material for: In situ graphene-seq: spatial transcriptomics and chronic electrophysiological characterization of tissue microenvironments
Source: Nat Commun. 2026 Jun 19;17:8382. doi: 10.1038/s41467-026-73883-7 (PMC13473619; doi:10.1038/s41467-026-73883-7)
Supplement: Supplementary file 4 [file 41467_2026_73883_MOESM6_ESM.pdf]

nature portfolio

Peer Review File

In Situ Graphene-Seq: Spatial Transcriptomics and Chronic Electrophysiological Characterization of Tissue Microenvironments

Corresponding Author: Professor Jia Liu

Parts of this Peer Review File have been redacted as indicated to remove third-party material.

This file contains all reviewer reports in order by version, followed by all author rebuttals in order by version.

Version 0:

Reviewer comments:

Reviewer #1

(Remarks to the Author)
Lee and Wang et al. present in situ graphene-seq, a technology that integrates electrophysiology with imaging-based spatial transcriptomics. Compared to their previous work (https://doi.org/10.1016/j.cell.2023.03.023), the main advancement here is the replacement of opaque electrodes with transparent graphene/PEDOT:PSS electrodes, facilitating optical imaging and making the integration of imaging and electrophysiology more straightforward. The platform was applied to a cardiomyocyte and endothelial cell co-culture model, revealing several cellular niches defined by electrophysiological activity and gene expression profiles. While this technology offers an interesting contribution to the spatial omics field, the current manuscript has significant limitations that, in my opinion, compromise its overall impact and clarity for the readership of Nature Communications.

Major Comments:

1. The central claim of this technology is its ability to integrate electrophysiology and transcriptomics at high spatiotemporal resolution. However, the manuscript does not clearly demonstrate how this integration leads to novel molecular insights or biological discoveries. The platform is applied to a relatively simple co-culture model, identifying three CM states and three niches, which largely mirror the same CM classification. This limited dataset does not substantiate the claim that the platform "captures functional and molecular heterogeneity within spatially heterogeneous tissue." It is essential for the authors to demonstrate that the inclusion of electrophysiological data provides insights that cannot be uncovered by spatial transcriptomics alone.

2. Although the technology enables temporal recording via electrophysiology followed by endpoint spatial transcriptomics, the manuscript does not illustrate how these multi-modal data layers contribute to new discoveries. Instead, the authors primarily rely on pseudotime analysis to infer cell states, bypassing the unique potential of the temporal electrophysiological recordings. Demonstrating how the temporal data layer enriches biological interpretation would highly strengthen the manuscript.

3. As a technological innovation in spatial profiling, most of the spatially resolved results rely on traditional IF staining rather than data directly obtained from in situ graphene-seq. This raises concerns about the robustness and reliability of the platform. While transparent electrodes facilitate optical imaging, the authors themselves acknowledge the increased electrochemical impedance, which compromises SNR. Although optimizations to reduce impedance were performed, the manuscript lacks sufficient data demonstrating the platform's reliability in resolving spatial patterns in complex biological systems.

4. The manuscript does not specify the size of the gene panel used (please correct me if I missed this). In the authors' previous work, >1,000 genes were included for the neural patch sequencing. It is unclear whether the integration of transparent electrodes affects the platform's capacity to handle similarly large gene panels for in situ sequencing. Clarification on this point is necessary to assess the platform's scalability and utility.

5. While the authors demonstrate scalable fabrication of the mesh nanoelectronics, they do not present data on how these larger devices integrate with imaging-based transcriptomics. The authors should demonstrate the feasibility of large-area recording and analysis across the entire workflow. Otherwise, I recommend removing the fabrication scalability data if it is not connected to transcriptomic integration.

Minor comments:

1. The manuscript frequently uses terms like “functional dynamics,” “functional outputs,” “functional changes,” and “cellular function” to describe the platform’s ability to capture both gene expression and electrophysiological states. However, electrophysiological recordings reflect only one aspect of cellular function. I suggest the authors adjust this wording to more accurately reflect the scope of functional readouts provided.

2. The section “Multimodal charting of electrophysiology, transcriptomics, and cell niches” reads more like a methodological description without presenting meaningful findings.

Reviewer #2

(Remarks to the Author)
1. The topic looks good, but not sure why 'chronic' should be used before 'electrophysiological characterization of tissue microenvironment.' Could the authors clarify the purpose or motivation of using the word chronic here?
2. Grammar and punctuation should be checked thoroughly. For example line 87 isn't complete.
3. Figure numbers should be continuous. It is rather confusing not to number the figures sequentially. For example, Lines #98 to 100 should be revised: 'Since there is a trade-off between transparency and impedance—both transmittance and electrochemical impedance decrease with increasing deposition time—the deposition time was optimized to balance these parameters (Methods, Extended Data Fig. 2c–f).' Rather than numbering as extended data, the manuscript should have unique figure numbers.
4. 'graphene/PEDOT electrodes': Authors should briefly explain what PEDOT and PSS mean initially in the intro section.
5. Statistical analysis in several plots shows significant overlap. However the authors mention a very high p value. The statistical analysis should be rechecked. The methodolgy should be discussed briefly in methods.

Reviewer #3

(Remarks to the Author)
In this manuscript, the authors developed a method for simultaneous capture of electrical potential and spatial transcriptome in tissue microenvironment using graphene electrodes that are flexible and transparent. This advancement facilitates seamless integration of electrophysiological recording and imaging. To validate the efficacy of the device, spatial sequencing was performed using 3D organoids, enabling a comprehensive analysis of the relationship between the cellular microenvironment, gene expression, and electrical activity. The methodology presented in the manuscript is potentially useful for discovery of novel cell-cell interaction in electrically active tissues. Overall, the study presents reliable data and well-documented findings, effectively demonstrating the device's functionality and potential applications. This reviewer would recommend additional analysis and information to further enhance the manuscript.

Major Comments

1. The manuscript indicates that clustering (cell type) (line 166) and pseudo-time analysis (line 498) were performed based on UMAP coordinates. However, this approach raises methodological concerns. While UMAP effectively visualizes high-dimensional data like scRNA-seq in lower dimensions, it is sensitive to local density distributions, potentially introducing artificial complexity to the data structure. Standard practice involves performing clustering and pseudo-time analyses in more faithful spaces (like PCA) before visualizing results with UMAP. Could the authors clarify if UMAP was performed before or after these analyses? If before, please verify that conclusions remain consistent when analyses are performed on pre-UMAP data.

2. The manuscript lacks sufficient detail regarding the methodology used to create UMAP from electrode waveform data (Fig.3d). While Fig.1b appears to show waveform feature points, no textual explanation accompanies this figure. Additionally, Fig.4a is described as clustering by molecularly defined niche, but the authors should explain what each column in the electrophysiology data represents. This information is crucial for understanding the analysis and interpreting the results.

3. The joint analysis of electrophysiology, gene expression, and local cell composition (line 247-250) represents the most significant analytical component of this study. However, the manuscript does not adequately describe how these qualitatively different data types were integrated into a unified analysis framework. Please provide detailed methodology on this critical aspect of the work.

4. While the comparison of gene expression across cardiomyocyte states includes early marker genes, ion channels, structure genes, and gap junction genes, several key structural genes that best reflect maturation status are missing. Please include data for TNNT2, MYH7 (beta-MHC), MYL2, and TNNI3 in Extended Fig.7, as these are essential for a comprehensive assessment of cardiomyocyte maturation.

Minor Comments
5. Typos in the text:
"silane" should be "saline" (line 360, 361)
"previous published methods" should be "previously published methods" (line 485)

6. Issues with figures:
Symbol mismatch in Fig.1c: Gene "n" versus Gene "l"
Compressed/illegible text in legends for Fig.2a, Fig.2c, and Fig.3b, specifically for “TNNT2", “NKX2.5" and “CD31”
Typographical error in Fig.4a legend: "Electrophysiolgical" should be "Electrophysiological" (missing "o")

Reviewer #4

(Remarks to the Author)
This study addresses an important challenge: how can tissue architecture be productively linked to functional outputs? One instance is how spatially distinct transcriptional states give rise to the emergent behavior of electrogenic tissue. In previous work, the authors addressed this by combining tissue-like mesh arrays of barcoded electrodes with spatial transcriptomics to bridge cell electrical and gene expression states. The incompatibility of opaque electrodes with imaging-based transcriptomics, however, precluded analysis of higher-order features, such as cell niches or microenvironments. Here, they use transparent electrodes, made of graphene, that overcome transmittance and impedance issues. This technical innovation now permits studying how cell niches within spatially heterogeneous tissue impact both electrical activity and gene regulation. Application to iPSC-derived cardiac microtissue reveals the composition and arrangement of cell microenvironments that foster functionally mature cardiomyocyte behavior, and impressive feat. Overall, this is an impactful novel technology, with high potential for applications in the study of spatial tissue biology, pathology, and regenerative medicine. The authors nonetheless need to clarify important technical issues, and address biological insights, before it can be considered suitable for publication.

Major comments:

The authors allude to improved spatial resolution over their previous platform “in situ electro-sequencing”, yet this is not quantified. How much better is it? What is the spatial precision metric that substantiates their assessment? Same with the efficiency of imaging data acquisition. How much more efficient is it?

The robustness of the technical innovation isn’t properly quantified either. Is it possible to quantify what is possible now, that wasn’t possible before, in terms of robustness of outcomes?

That transparent electrodes lower the barrier to optical imaging applications is self-evident. Yet the pair of E-barcodes positioned near each electrode occupy a similar total area than the electrode itself. Don’t they obstruct the optics needed to image the neighborhood of the recorded cell, akin to opaque electrodes?

How are single cell signals registered? It appears that many cells can interface with the same electrode, including of the same cell type.

Given that “unidentified” cells are a major determinant of what makes niche 3 different, they must be characterized. What are their markers? The markers that distinguish each cell type should be presented, and best-guess predictions of cell identity should be provided.

The ratio of endothelial to fibroblast cells seems another distinctive niche 3 feature. Is it possible to regress this ratio against transcriptional /electrophysiological /joint pseudotime across all niches? What should be the most desirable ratio for future microtissue design?
Is predictive inference possible – i.e. predicting molecular / functional state from cell niche?

Minor comments:

Light transmittance is measured to be less than 85% in the optimized graphene + PEDOT:PSS electrodes. Is this due to decreased light absorption, scattering, or diffraction, and if so can this improved by improving material purity or patterning?

What is the difference in imaging resolution for cells behind vs. in front of the transparent electrodes? How do both compare with cells outside of the electrode perimeter?

The CM-only and CM/EC co-culture within the same device is creative – do CMs across the two different regions beat synchronously? This is not quantified either within or across regions.

The authors highlight interesting genes upregulated across different niches, such as ion channels. What about their proteins,

can those be validated? Seems compatible with their technology.

Fig.1a - instead of using the word ‘ch’ use ‘Channel’

Fig.2j – is the decrease in FPD indicate maturation, or is there a decrease in viability of the cells in the mesh

Fig.3b - Antibody labels are blurry, and CD31 expression in the co-culture sample seems skewed on one side – is this at the co-culture to CM-only interface? If so this should be indicated.

Fig.5f – Need better contrast

In line 195, the authors relate higher spike amplitudes, greater maximum dV/dt, and shorter FPD observed in CM/EC culture to greater functional maturation. What is the evidence that these parameters are associated with maturation in vivo?

Reviewer #5

(Remarks to the Author)
I co-reviewed this manuscript with one of the reviewers who provided the listed reports. This is part of the Nature Communications initiative to facilitate training in peer review and to provide appropriate recognition for Early Career Researchers who co-review manuscripts.

Version 1:

Reviewer comments:

Reviewer #1

(Remarks to the Author)
The authors have properly responded to my concerns and I have no further questions.

(Remarks on code availability)

Reviewer #3

(Remarks to the Author)
The manuscript addresses my concerns. I have no further comments.

(Remarks on code availability)

Reviewer #4

(Remarks to the Author)
All my comments have been addressed, except side-by-side comparisons with the author’s previous technology iteration. The revised manuscript is therefore acceptable for publication, but the authors are still suggested to make minor revisions as follows:

1. The authors argue that in their previous approach, which relied on opaque metal electrodes, the additional handling steps required “often disrupted the native cell microenvironment” such that “successful imaging becomes highly variable“ – can examples be provided? i.e. of native cell microenvironment disruptions and unsuccessful imaging due to the extra steps, or quantification of imaging success variability.

2. Is it possible to apply the new Supplementary Fig. 4C quantification to the previous (opaque electrodes) approach, to compare their imaging resolution side-by-side?

(Remarks on code availability)

Reviewer #5

(Remarks to the Author)
I co-reviewed this manuscript with one of the reviewers who provided the listed reports. This is part of the Nature Communications initiative to facilitate training in peer review and to provide appropriate recognition for Early Career Researchers who co-review manuscripts.

(Remarks on code availability)

Reviewer #6

(Remarks to the Author)
I co-reviewed this manuscript with one of the reviewers who provided the listed reports. This is part of the Nature Communications initiative to facilitate training in peer review and to provide appropriate recognition for Early Career Researchers who co-review manuscripts.

(Remarks on code availability)
The code link provided a README file with instructions. I would encourage the authors to include more notes to improve readability. The code can be reproduced based on the code posted on GitHub.

Open Access This Peer Review File is licensed under a Creative Commons Attribution 4.0 International License, which permits use, sharing, adaptation, distribution and reproduction in any medium or format, as long as you give appropriate credit to the original author(s) and the source, provide a link to the Creative Commons license, and indicate if changes were made.
In cases where reviewers are anonymous, credit should be given to 'Anonymous Referee' and the source.
The images or other third party material in this Peer Review File are included in the article's Creative Commons license, unless indicated otherwise in a credit line to the material. If material is not included in the article's Creative Commons license and your intended use is not permitted by statutory regulation or exceeds the permitted use, you will need to obtain permission directly from the copyright holder.
To view a copy of this license, visit https://creativecommons.org/licenses/by/4.0/

REVIEWER COMMENTS

We sincerely thank the Editor and all reviewers for their careful evaluation of our manuscript and for the many constructive comments that have helped strengthen this work.

We were delighted to receive many positive comments from the reviewers, for example, *"this technology offers an interesting contribution to the spatial omics field, …"*; *"the methodology presented in the manuscript is potentially useful for discovery of novel cell-cell interaction in electrically active tissues."*; "*overall, the study presents reliable data and well-documented findings, effectively demonstrating the device's functionality and potential applications"*; and *"overall, this is an impactful novel technology, with high potential for applications in the study of spatial tissue biology, pathology, and regenerative medicine."*

We have carefully addressed each comment in full, as detailed below. We thank the Editor and reviewers again for their time and thoughtful consideration.

Response to Reviewer #1 Comments:

General Comment: Lee and Wang et al. present in situ graphene-seq, a technology that integrates electrophysiology with imaging-based spatial transcriptomics. Compared to their previous work (https://doi.org/10.1016/j.cell.2023.03.023), the main advancement here is the replacement of opaque electrodes with transparent graphene/PEDOT:PSS electrodes, facilitating optical imaging and making the integration of imaging and electrophysiology more straightforward. The platform was applied to a cardiomyocyte and endothelial cell co-culture model, revealing several cellular niches defined by electrophysiological activity and gene expression profiles. While this technology offers an interesting contribution to the spatial omics field, the current manuscript has significant limitations that, in my opinion, compromise its overall impact and clarity for the readership of Nature Communications.

Response: We appreciate the reviewer's thoughtful and constructive feedback. In response, we have clarified the scope of this work, strengthened the rationale for the engineered hiPSC-CM/hiPSC-EC co-culture model as a controlled benchmarking system. We also revised figures and terminology to enhance clarity of the manuscript. We have carefully addressed all the reviewer's comments, as detailed in our point-by-point response below.

Comment-1: The central claim of this technology is its ability to integrate electrophysiology and transcriptomics at high spatiotemporal resolution. However, the manuscript does not clearly demonstrate how this integration leads to novel molecular insights or biological discoveries. The platform is applied to a relatively simple co-culture model, identifying three CM states and three niches, which largely mirror the same CM classification. This limited dataset does not substantiate the claim that the platform "captures functional and molecular heterogeneity within spatially heterogeneous tissue." It is essential for the authors to demonstrate that the inclusion of electrophysiological data provides insights that cannot be uncovered by spatial transcriptomics alone.

Response: We thank the reviewer for this critical perspective regarding the biological novelty and the added value of integrating electrophysiology with spatial transcriptomics. We would like to clarify that the primary goal of this manuscript is to introduce and validate *in situ* graphene-seq. Accordingly, we designed a controlled co-culture system to establish the fidelity of co-registration between electrophysiological signals and molecular readouts.

A key motivation for our approach is well-established in prior multimodal single-cell work. Previous Patch-seq studies[1,2] has shown that transcriptomic identity alone cannot fully predict electrophysiological phenotype. Likewise, while spatial transcriptomics can infer molecular state and neighborhood relationships at the moment of fixation, it cannot directly measure electrical behavior or dynamics. By recording and correlating electrophysiological signals with spatial molecular information *in situ*, our platform adds a functional electrophysiological layer that enables evaluation of whether molecularly defined niches exhibit distinct electrical behaviors (**Fig. 4**).

To further assess whether electrophysiological signals encode biologically meaningful information, we performed predictive inference of spatial niche identity using either molecular or electrophysiological features. While gene expression achieved 98.24% prediction accuracy, electrophysiological features alone achieved 82.35% accuracy (**Response Fig. 1-1**).

Together, this co-culture model serves as a proofs-of-principle demonstrating that electrophysiological features can be linked to molecularly defined spatial states. These results highlight the potential to (i) directly study electrophysiological heterogeneity at the niche level and (ii) develop cross-modal inference models linking electrophysiological features with molecular and spatial information.

We agree that extending this approach to larger datasets and more complex tissues is an important next step and have revised the Discussion accordingly.

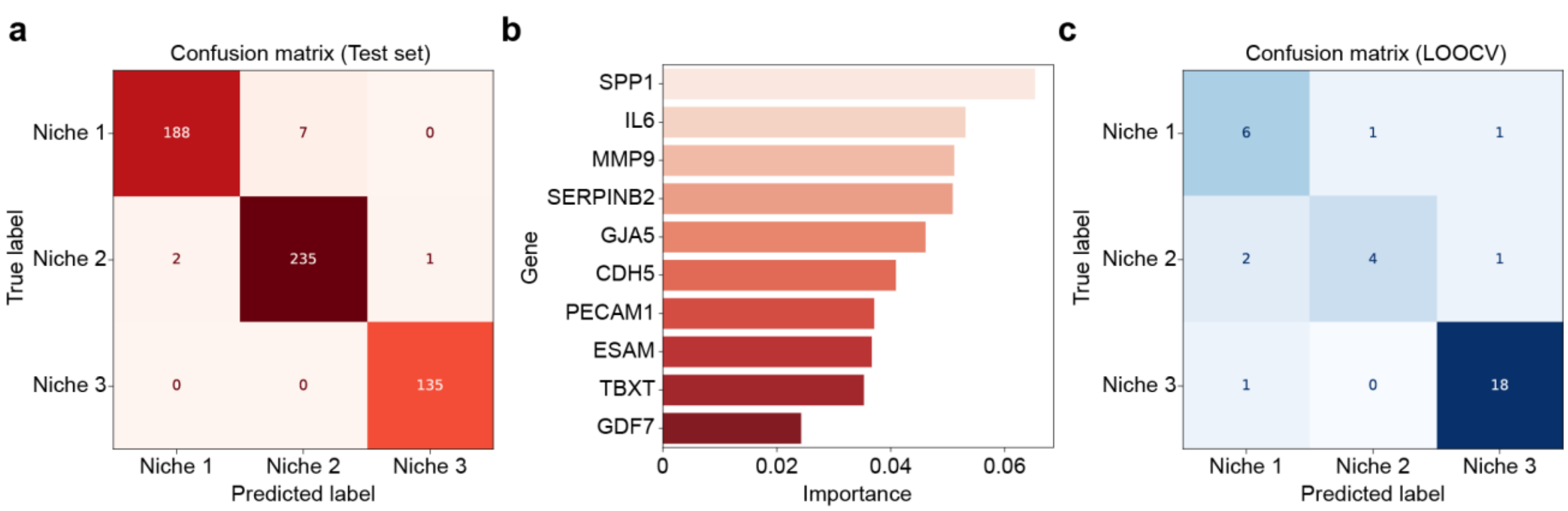

Response Fig. 1-1: Predictive inference of spatial niche from molecular and electrophysiological features. a, Confusion matrix of the random forest classifier trained on gene expression data to predict spatial niche identity of cardiomyocytes, evaluated on a held-out test set (80/20 split). **b**, Ranked feature importance of the top genes contributing to niche prediction. **c**, Confusion matrix of the random forest classifier trained on electrophysiological features, evaluated using leave-one-out cross-validation (LOOCV).

Revision to the manuscript:

We have revised the manuscript to clarify the scope of this study and rationale for engineered heterogeneous co-culture model.

"The spatially resolved, multimodal integration workflow of in situ graphene-seq makes it uniquely suited for investigating spatially heterogeneous tissues, where molecular identity and electrophysiological phenotype may diverge across the tissue. To validate whether our platform could resolve such spatial heterogeneity, we designed a controlled and biologically interpretable heterogenous tissue model.

We engineered spatial heterogeneity by co-culturing hiPSC-CMs and hiPSC-derived endothelial cells (hiPSC-ECs), building on prior studies showing that co-culture with hiPSC-ECs promotes the electrical

maturation of hiPSC-CMs[23]. To create a defined spatial pattern, we seeded hiPSC-CMs and hiPSC-ECs at a 3:1 ratio on one side of the mesh electronics, while seeding only hiPSC-CMs on the opposite side (Methods, Fig. 3a). We expected this design to yield interpretable and spatially localized differences in the electrical and molecular signatures of hiPSC-CMs. To validate the differences in cell composition, we performed immunohistochemical analysis. Staining for cardiac troponin T (TNNT2) and cluster of differentiation 31 (CD31) confirmed the intended cellular distributions, with higher densities of CD31-positive hiPSC-ECs localized to the co-culture region (Fig. 3b).''

We also incorporated **Response Fig. 1-1** into the Supplementary Information as **Supplementary Fig. 10** and expanded the discussion to highlight the potential of integrating electrophysiology with spatial transcriptomic analysis.

''Beyond descriptive analysis, this multimodal platform potentially enables cross-modal inference between molecular and electrophysiological domains. To explore this capability, we performed proof-of-concept predictive inference of spatial niche identity using either molecular or electrophysiological features (Methods). A random forest classifier trained on gene expression data achieved 98.24% accuracy on a held-out test set (Supplementary Fig. 10a,b). Using electrophysiological features alone, the classifier predicted niche identity with 82.35% accuracy (Supplementary Fig. 10c). Together, these results suggest that electrophysiological recordings encode information associated with molecularly defined spatial niches, highlighting the potential for cross-modal inference enabled by this platform.''

''Beyond the proof-of-concept demonstration in this manuscript, such approaches may enable prediction of molecular states from electrical dynamics, early detection of niche-dependent maturation, or elucidation of how multicellular composition, including support cell densities, influences electrophysiological and molecular maturation, potentially providing quantitative guidance for microtissue design.''

Comment-2: Although the technology enables temporal recording via electrophysiology followed by endpoint spatial transcriptomics, the manuscript does not illustrate how these multi-modal data layers contribute to new discoveries. Instead, the authors primarily rely on pseudotime analysis to infer cell states, bypassing the unique potential of the temporal electrophysiological recordings. Demonstrating how the temporal data layer enriches biological interpretation would highly strengthen the manuscript.

Response: We thank the reviewer for this thoughtful comment. We would like to clarify that in this study, temporal electrical recording served as an enabling capability that defined the experimental timeline and made multimodal integration possible.

In the CM/EC co-culture experiments (**Fig. 3–5**), hiPSC-CMs were first seeded on one half of the device, followed by seeding of hiPSC-CM/EC co-cultures on the opposite half on the following day. The tissue was then cultured until both regions grew together and began to exhibit synchronized beating activity. Electrophysiological activity was periodically monitored to determine when coordinated electrical behavior emerged. This functional convergence defined the time point for electrophysiological data acquisition and subsequent spatial transcriptomic analysis. A platform limited to one-time or short-term recordings would be unable to track this progression and therefore could not establish the appropriate conditions for multimodal spatial profiling.

In addition, electrophysiological recordings acquired immediately prior to fixation provide the functional context for interpreting spatial transcriptomic states, validating that molecularly defined niches correspond to distinct electrical phenotypes, consistent with the co-culture design (**Fig. 4**). As noted in our response to Comment-1, electrophysiological features may encode biologically meaningful spatial information, supporting proof-of-concept cross-modal inference.

Therefore, while the present study does not integrate longitudinal electrophysiology with multiple end point transcriptomic measurements, it establishes and validates both temporal monitoring capability and spatial multimodal profiling within a single platform. This provides a necessary foundation for future discovery-driven studies. We agree with the reviewer that this integration represents a key opportunity and have added statements to the Discussion accordingly.

Revision to the manuscript:

"Although this study focused on validating our platform in a relatively simple and predictable heterogeneous tissue model, applying in situ graphene-seq in vivo may further enable multimodal mapping of brain and cardiac systems at high spatiotemporal resolution.

Longitudinal multimodal datasets generated by in situ graphene-seq will also be well suited for artificial intelligence (AI)-driven analyses—which have already demonstrated strong capabilities in extracting biological and biomedical insights from high-dimensional data[48,49,50]. Beyond the proof-of-concept demonstration in this manuscript, such approaches may enable prediction of molecular states from electrical dynamics, early detection of niche-dependent maturation, or elucidation of how multicellular composition, including support cell densities, influences electrophysiological and molecular maturation, potentially providing quantitative guidance for microtissue design."

Comment-3: As a technological innovation in spatial profiling, most of the spatially resolved results rely on traditional IF staining rather than data directly obtained from in situ graphene-seq. This raises concerns about the robustness and reliability of the platform. While transparent electrodes facilitate optical imaging, the authors themselves acknowledge the increased electrochemical impedance, which compromises SNR. Although optimizations to reduce impedance were performed, the manuscript lacks sufficient data demonstrating the platform's reliability in resolving spatial patterns in complex biological systems.

Response: We believe here is a misunderstanding. We would like to clarify that immunofluorescence (IF) staining was not used as the primary source of spatial information in this study. IF was performed *only* to validate the heterogeneity of the co-culture model (hiPSC-CM and hiPSC-EC distribution) and to confirm that the expected spatial organization of cell types was present within the model.

All spatially resolved results presented in the manuscript, including molecular and multimodal niche identification, cell-state mapping, and the alignment of electrophysiological activity with molecular signatures, were obtained directly from *in situ* graphene-seq data. The IF images were used only as a validation tool to confirm that the spatial patterns detected by our platform reflected genuine biological heterogeneity rather than technical artifacts.

Regarding electrode impedance, we acknowledge that transparent graphene/PEDOT:PSS electrodes exhibit higher baseline impedance than opaque metal electrodes. However, by optimizing PEDOT:PSS functionalization, we achieved stable, high signal-to-noise recordings over extended time periods (**Fig. 2e–j**), reliably capturing extracellular field potentials and waveform features across 3D tissues. These results confirm that maintaining optical transparency does not compromise electrical signal quality.

Comment-4: The manuscript does not specify the size of the gene panel used (please correct me if I missed this). In the authors' previous work, >1,000 genes were included for the neural patch sequencing. It is unclear whether the integration of transparent electrodes affects the platform's capacity to handle similarly large gene panels for in situ sequencing. Clarification on this point is necessary to assess the platform's scalability and utility.

Response: We thank the reviewer for this important question regarding the scalability of the gene panel and the potential impact of transparent electrodes on *in situ* sequencing capacity.

First, regarding the specific gene panels used in this study: For the initial validation data presented in **Supplementary Fig. 4-6**, we utilized a 201-gene panel, consistent with the panel design from our previous work[3]. For the CM/EC co-culture experiments (shown in **Figs. 3–5**), we designed a targeted 254-gene panel. This panel size was selected solely to facilitate the most differentially expressed genes in the hiPSC-CMs and ECs. The targeted panel was curated to provide comprehensive coverage of key cardiomyocyte maturation markers, ion channels, structural genes, and major endothelial markers necessary to characterize the cellular niches in this study.

Second, regarding the scalability and compatibility with > 1,000 genes: We confirmed that the integration of graphene/PEDOT:PSS electrodes does not introduce optical hindrance or chemical incompatibility that would limit the multiplexing capability of STARmap to > 1,000 genes. The gene throughput of the STARmap platform is determined by the encoding capability of the SEDAL (Sequencing with Error-Reduction by Dynamic Annealing and Ligation) scheme[4]. Scaling from ~200 genes to > 1,000 genes primarily require increasing the barcode length from 4 bits to 5 bits, which necessitates one additional round of sequencing chemistry and imaging (e.g., from 5 rounds to 6 rounds), rather than changing the fundamental chemistry or imaging conditions.

To empirically demonstrate that our platform can withstand the extended chemical and optical workflow required for > 1,000 genes, we performed a validation experiment using a 4-gene STARmap panel encoded with 5-bit barcodes. This experimental setup requires 6 rounds of imaging to decode, which is identical to the workflow required for a > 1,000-gene library. As shown in **Response Fig. 1-2**, we successfully resolved clear amplicons directly on top of the graphene/PEDOT:PSS electrodes across all 6 rounds. The SNR remained high, and the electrodes did not interfere with the fluorescence signal or the sequential ligation chemistry.

These results confirm that *in situ* graphene-seq is fully compatible with the multi-round imaging requirements for large-scale gene panels (> 1,000 genes).

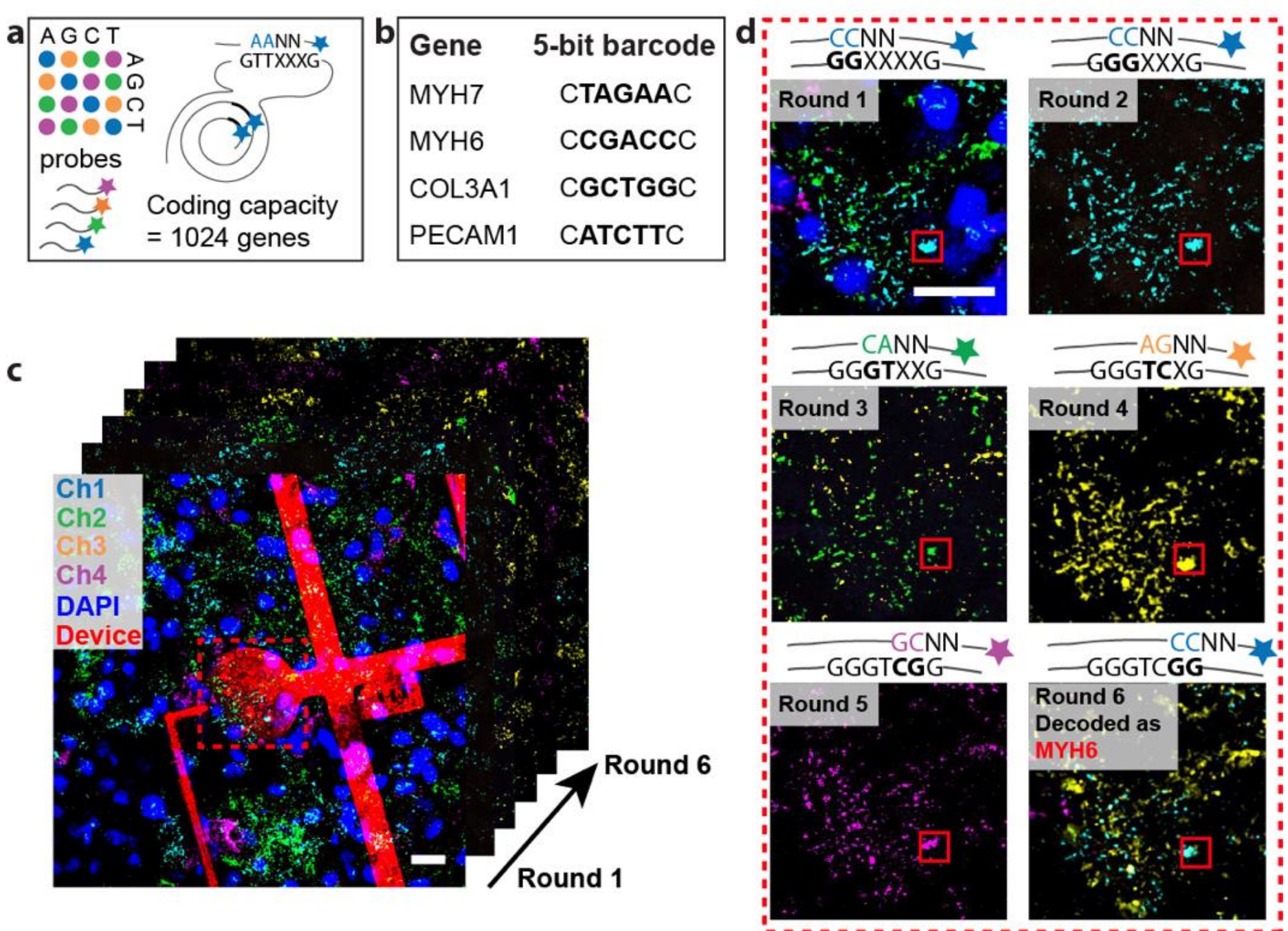

Response Fig. 1-2: *In situ* graphene-seq with 5-bit gene barcodes. a, Schematic of the decoding process using 5-base barcodes, including degenerate bases at both the 5' and 3' ends. **b**, Gene panel and barcode design for 5-bit *in situ* graphene-seq. **c**, Representative images from the six sequencing cycles. The red dashed box indicates the graphene electrode region. **d**, Zoom-in view of the boxed region in panel **c**, with

the device channel omitted to highlight the amplicons. Scale bar, 20 µm for panels **c-d.** Clear amplicons were resolved directly on top of electrodes across all six rounds, with high SNR and no detectable interference with fluorescence imaging or sequential ligation chemistry. These results demonstrate compatibility of *in situ* graphene-seq with multi-round imaging workflows required for large-scale spatial transcriptomic panels.

Revision to the manuscript:

We have incorporated **Response Fig. 1-2** into Supplementary Information as **Supplementary Fig. 11** and discussed gene set information and multi-round imaging compatibility in the Method section.

"For the hiPSC-CM-only sample, we utilized a 201-gene panel identical to that employed in our previous study[24]. For the hiPSC-CM/hiPSC-EC co-culture experiments, we designed a targeted 254-gene panel (Supplementary Data 1), curated to include key CM maturation markers, ion channels, structural genes, and major endothelial markers to ensure sufficient resolution for niche characterization.

To empirically validate compatibility with extended chemical and optical workflows required for large gene panels (> 1,000 genes), we performed a validation experiment using a 4-gene STARmap panel encoded with 5-bit barcodes (Supplementary Fig. 11). This encoding scheme requires six sequential imaging rounds for decoding, matching the workflow required for > 1,000-gene libraries. Clear amplicons were resolved directly on top of graphene/PEDOT:PSS electrodes across all six rounds, with high SNR and no detectable interference with fluorescence imaging or sequential ligation chemistry."

Comment-5: While the authors demonstrate scalable fabrication of the mesh nanoelectronics, they do not present data on how these larger devices integrate with imaging-based transcriptomics. The authors should demonstrate the feasibility of large area recording and analysis across the entire workflow. Otherwise, I recommend removing the fabrication scalability data if it is not connected to transcriptomic integration.

Response: We thank the reviewer for this constructive comment. We agree that, since the scalable mesh devices were not used for transcriptomic integration in this study, including the corresponding figure and paragraph could be misleading. We have therefore removed **formerly Extended Data Fig. 3** and the associated text from the manuscript.

Revision to the manuscript:

In the revised manuscript, we discuss scalability only as fabrication context and future potential, rather than as a demonstrated capability.

"The fabrication process is readily scalable, as all steps are compatible with conventional 2D microfabrication techniques. Moreover, recent advances in graphene production and transfer techniques enable the reliable, large-scale integration of graphene electrodes into such devices[33,34,35], supporting the potential of in situ graphene-seq for broad and customizable applications."

Minor comments:

Comment-6: The manuscript frequently uses terms like "functional dynamics," "functional outputs," "functional changes," and "cellular function" to describe the platform's ability to capture both gene expression and electrophysiological states. However, electrophysiological recordings reflect only one aspect of cellular function. I suggest the authors adjust this wording to more accurately reflect the scope of functional readouts provided.

Response: We thank the reviewer for this insightful comment. We agree that electrophysiological recordings represent one aspect of cellular function and that our terminology should more precisely reflect the scope of our platform. In the revised manuscript, we have replaced broad terms such as “functional dynamics”, “functional outputs”, “functional changes”, and “cellular function” with more accurate phrasing, including “electrophysiological activity”, “electrophysiological dynamics”, “cellular activity”, or “electrophysiological and molecular characteristics”, depending on the context.

Comment-7: The section “Multimodal charting of electrophysiology, transcriptomics, and cell niches” reads more like a methodological description without presenting meaningful findings.

Response: We thank the reviewer for this helpful comment. We have revised the manuscript to streamline this section, emphasizing the outcomes of multimodal integration while moving technical descriptions (e.g., tissue processing steps, segmentation methods, and electrode reconstruction details) to the Methods.

Revision to the manuscript:

*“**Multimodal mapping of electrophysiology, transcriptomics, and cell niches***

In situ graphene-seq integrates electrophysiological recordings with imaging-based in situ sequencing on a single platform, eliminating intermediate handling steps and preserving spatial fidelity throughout the processes. To establish a streamlined framework for multimodal data acquisition, we first optimized this workflow using homogeneous hiPSC-derived cardiac microtissues, allowing direct evaluation of multimodal data integration without confounding spatial variability.

We integrated cardiac microtissues with stretchable and transparent mesh electronics to obtain extracellular recordings, which were processed to extract electrophysiological features (Methods). Immediately after recordings, the same tissue/electronics hybrid was fixed and processed for in situ complementary DNA (cDNA) library preparation and sequencing, enabling spatially resolved transcriptomic profiling in situ (Methods). Consistent with the optical transparency of the electrodes, no detectable loss of imaging resolution was observed for cells cultured above or below the electrodes compared with regions outside the electrode perimeter (Supplementary Fig. 4). Fluorescent E-barcodes positioned adjacent to electrodes enabled precise co-registration of sequencing reads with electrical recordings.

To link molecular and electrophysiological information, we optimized 3D cell segmentation and assigned amplicon reads to individual cells (Methods, Supplementary Fig. 5). Major cell types were identified by clustering of transcriptomic profiles, and electrically recorded cells were localized by spatial alignment of segmented cells with electrode positions. This workflow established a direct mapping between molecular signatures, local cellular environments, and electrophysiological recordings (Supplementary Fig. 6).”

Response to Reviewer #2 Comments:

We thank the reviewer for their careful review of our manuscript. In response, we corrected typographical errors, improved grammatical clarity and figure consistency with *Nature Communications* style guidelines, and revisited statistical analyses to enhance transparency of the presented data. Below are our point-by-point responses to individual comments.

Comment-1: The topic looks good, but not sure why 'chronic' should be used before 'electrophysiological characterization of tissue microenvironment.' Could the authors clarify the purpose or motivation of using the word chronic here?

Response: We thank the reviewer for raising this point. We agree that "chronic" can be ambiguous if not explicitly defined. Here, we use "chronic" in its standard electrophysiology/implant literature sense to indicate stable, repeated recordings from the same tissue-electrode interface over extended time periods, rather than a one-time or acute measurement.

Our motivation for using the term is to highlight a key technical capability enabled by the flexible, transparent graphene/PEDOT:PSS electrodes: the device maintains a stable interface with the tissue without compromising viability or spatial integrity, allowing long-term electrophysiological monitoring from the same region for more than 2 weeks (**Fig. 2e–j**). This is an important differentiator of the platform and is a prerequisite for longitudinal multimodal experiments.

In the present study, electrophysiological activity was periodically assessed over time to determine when heterogeneously integrated tissue regions began to exhibit coordinated beating, which then defined the time point for data acquisition and subsequent spatial transcriptomic analysis. A platform limited to short-term or acute recordings would be unable to establish this temporal context and therefore could not support the multimodal spatial profiling demonstrated here.

To avoid confusion, we are happy to revise the phrasing to make the intended meaning explicit. For example, replacing "chronic electrophysiological characterization" with "long-term, stable electrophysiological characterization" (or "longitudinal electrophysiological characterization") and adding a brief definition at first use.

Revision to the manuscript:

We have discussed future direction of the chronic electrophysiological recording capabilities in the Discussion.

"The demonstrated stability of long-term recordings further positions the system for longitudinal multimodal experiments."

"Longitudinal multimodal datasets generated by in situ graphene-seq will also be well suited for artificial intelligence (AI)-driven analyses—which have already demonstrated strong capabilities in extracting biological and biomedical insights from high-dimensional data[48,49,50]. Beyond the proof-of-concept demonstration in this manuscript, such approaches may enable prediction of molecular states from electrical dynamics, early detection of niche-dependent maturation, or elucidation of how multicellular composition, including support cell densities, influences electrophysiological and molecular maturation, potentially providing quantitative guidance for microtissue design."

Comment-2: Grammar and punctuation should be checked thoroughly. For example, line 87 isn't complete.

Response: We appreciate the reviewer's careful reading of the manuscript. We have thoroughly re-checked the grammar and punctuation throughout the manuscript and corrected inconsistencies where necessary. The sentence at line 87 has also been revised to ensure it is complete and clear in the revised manuscript.

Comment-3: Figure numbers should be continuous. It is rather confusing not to number the figures sequentially. For example, Lines #98 to 100 should be revised: 'Since there is a trade-off between transparency and impedance—both transmittance and electrochemical impedance decrease with increasing deposition time—the deposition time was optimized to balance these parameters (Methods, Extended Data Fig. 2c–f).' Rather than numbering as extended data, the manuscript should have unique figure numbers.

Response: We thank the reviewer for this helpful suggestion. We carefully revisited the formatting guidelines of *Nature Communications*. In the revised manuscript, all former Extended Data figures have been moved to the Supplementary Information and are now cited as Supplementary Fig. 1, Supplementary Fig. 2, etc., in the main text. The main figures remain sequentially numbered within the manuscript, while Supplementary figures are numbered separately in accordance with the journal's guidelines.

Comment-4: 'graphene/PEDOT electrodes': Authors should briefly explain what PEDOT and PSS mean initially in the intro section.

Response: We thank the reviewer for this helpful suggestion. PEDOT:PSS is now introduced as *poly(3,4-ethylenedioxythiophene) polystyrene sulfonate)* when first mentioned in the manuscript.

Revision to the manuscript:

We have revised the Abstract and Introduction to include the full names of the materials when first used.

"The system combines stretchable mesh nanoelectronics for long-term, single-cell-level interfacing with transparent graphene/poly(3,4-ethylenedioxythiophene) polystyrene sulfonate electrodes, enabling seamless integration of electrical recordings and optical imaging."

"By incorporating transparent graphene/PEDOT:PSS (poly(3,4-ethylenedioxythiophene) polystyrene sulfonate) electrodes into the mesh, the platform maintains optical clarity, enabling direct imaging of cells on top of the electrodes regardless of orientation."

Comment-5: Statistical analysis in several plots shows significant overlap. However, the authors mention a very high p value. The statistical analysis should be rechecked. The methodolgy should be discussed briefly in methods.

Response: We thank the reviewer for this insightful comment. We have carefully re-evaluated all statistical analyses to ensure the appropriate tests were applied based on data structure and distribution. In **Fig. 1e**, measurements represent paired samples before and after treatment and did not follow a normal distribution. Therefore, we used a two-tailed Wilcoxon matched-pairs signed-rank test. For **Supplementary Fig. 2f**, impedance values were obtained from independent samples and were not normally distributed. Accordingly, we used an unpaired two-tailed Mann-Whitney test and updated the corresponding p-values. For **Fig. 3e-g**, which compare two regions within samples and exhibited non-normal distributions, we again applied two-tailed Mann-Whitney tests. For **Fig. 4b-d**, although the data were normally distributed, they violated the equal-variance assumption and involved modest sample sizes. Therefore, we used Kruskal-Wallis tests to assess differences across niches. All revised p-values have been verified and are reported in the updated figures and captions.

Revision to the manuscript:

We have added a brief description of the statistical methodology in the Methods section and clarified the specific analyses in the figure captions.

"Statistical analysis. All statistical analyses were performed using GraphPad Prism. Data normality was assessed with the Shapiro-Wilk test, and appropriate nonparametric or parametric tests were applied accordingly. For comparisons between two independent groups, the two-tailed Mann-Whitney U test was used, and for paired comparisons, the Wilcoxon signed-rank test was applied. For comparisons among three niches, data were analyzed using the Kruskal-Wallis test, which is robust to small sample sizes and unequal variances. Details of the specific statistical tests and corresponding p-values are provided in the figure captions."

Response to Reviewer #3 Comments:

General Comment: In this manuscript, the authors developed a method for simultaneous capture of electrical potential and spatial transcriptome in tissue microenvironment using graphene electrodes that are flexible and transparent. This advancement facilitates seamless integration of electrophysiological recording and imaging. To validate the efficacy of the device, spatial sequencing was performed using 3D organoids, enabling a comprehensive analysis of the relationship between the cellular microenvironment, gene expression, and electrical activity. The methodology presented in the manuscript is potentially useful for discovery of novel cell-cell interaction in electrically active tissues. Overall, the study presents reliable data and well-documented findings, effectively demonstrating the device's functionality and potential applications. This reviewer would recommend additional analysis and information to further enhance the manuscript.

Response: We thank the reviewer for their insightful comments. In response, we revisited and refined our analysis framework to ensure that clustering, trajectory, and pseudotime calculations were performed in appropriate and scientifically valid feature spaces. We also clarified data processing methodologies, improved the transparency of the analyses, and corrected typographical and grammatical errors throughout the manuscript. Below, we provide point-by-point responses to the reviewer's comments.

Major Comments

Comment-1: The manuscript indicates that clustering (cell type) (line 166) and pseudo-time analysis (line 498) were performed based on UMAP coordinates. However, this approach raises methodological concerns. While UMAP effectively visualizes high-dimensional data like scRNA-seq in lower dimensions, it is sensitive to local density distributions, potentially introducing artificial complexity to the data structure. Standard practice involves performing clustering and pseudo-time analyses in more faithful spaces (like PCA) before visualizing results with UMAP. Could the authors clarify if UMAP was performed before or after these analyses? If before, please verify that conclusions remain consistent when analyses are performed on pre-UMAP data.

Response: We appreciate the reviewer's insightful comment. We carefully revisited our analysis pipeline to ensure that clustering and trajectory analyses were performed in the appropriate feature spaces.

We first confirmed that both cell-type clustering and niche clustering (Leiden) were conducted on the k-nearest neighbor (kNN) graph computed in PCA space, not on UMAP embeddings. UMAP was generated using the same neighbor graph but was applied exclusively for visualization.

For transcriptional pseudotime analysis, we note that our initial implementation used UMAP embeddings. To address the reviewer's concern, we reanalyzed pseudotime using PCA space. Specifically, gene expression profiles of all sequenced cardiomyocytes were embedded in PCA space, followed by kNN graph construction. Leiden cluster assignments from this graph were used as input labels to guide lineage inference. PCA embeddings and cluster labels were exported to R and processed through Slingshot, which fitted a principal curve to estimate pseudotime trajectories. The resulting pseudotime values were normalized to a [0, 1] range for comparability. Electrically recorded cells were selected after pseudotime calculation and visualized using UMAP (**Response Fig. 3-1**). The overall trajectories and relative trend of cardiomyocytes remained highly consistent with the results from the original analysis.

For the electrophysiological pseudotime, we similarly recalculated pseudotime in PCA space using the same approach, which yielded the same overall trend as the original analysis (**Response Fig. 3-2**). However, given the limited sample size (n=34), we recognize that pseudotime inference may be less robust in this dataset. Therefore, in the revised manuscript, we used the first principal component (PC1, explaining 54.1% of total variance) as a linear progression score across niches (**Response Fig. 3-3**).

For the multimodal clustering (**Fig. 5**), we confirmed that clustering was consistently performed using Leiden clustering on PCA embeddings, not on UMAP embeddings.

Importantly, these methodological clarifications and reanalysis did not alter the results or conclusions of the study. All inferred trajectories, trends across niches, and multimodal relationships remained consistent with the original findings.

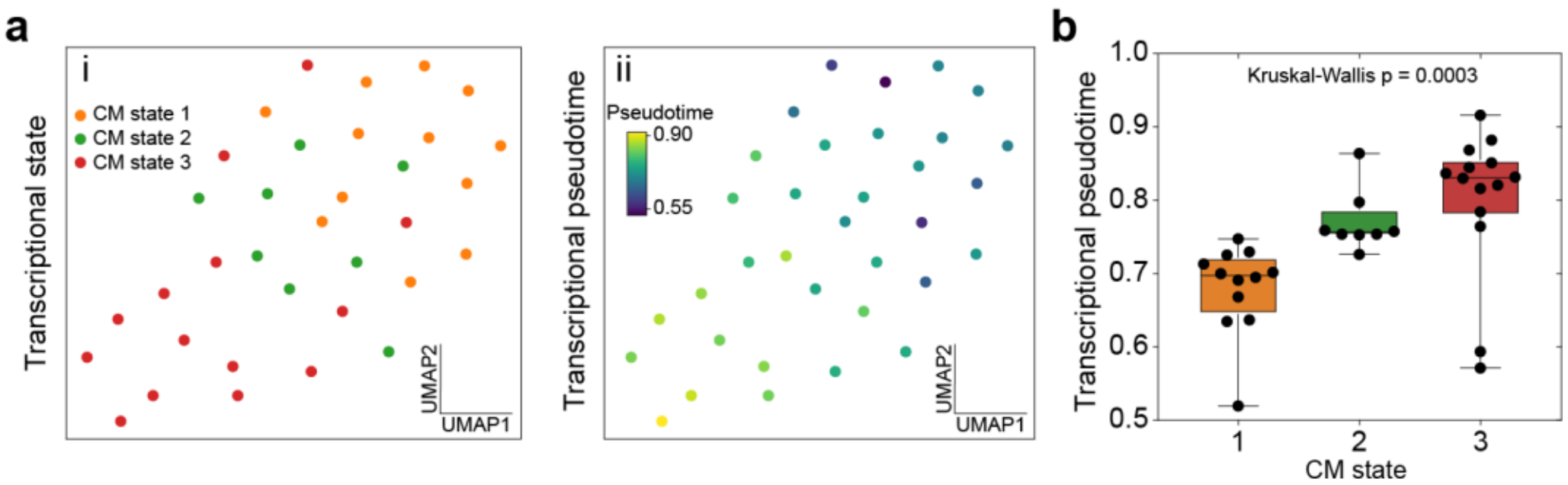

Response Fig. 3-1: Transcriptional pseudotime computed in PCA space. a, UMAP embedding of gene expression profiles of electrically recorded hiPSC-CMs, color-coded by cell states (i) and transcriptional pseudotime (ii). **b**, Box-and-whisker plot showing the distribution of transcriptional pseudotime values across different hiPSC-CM states. P-values were calculated from Kruskal-Wallis test. Sample sizes: CM_s1 (n=12), CM_s2 (n=8), CM_s3 (n=14).

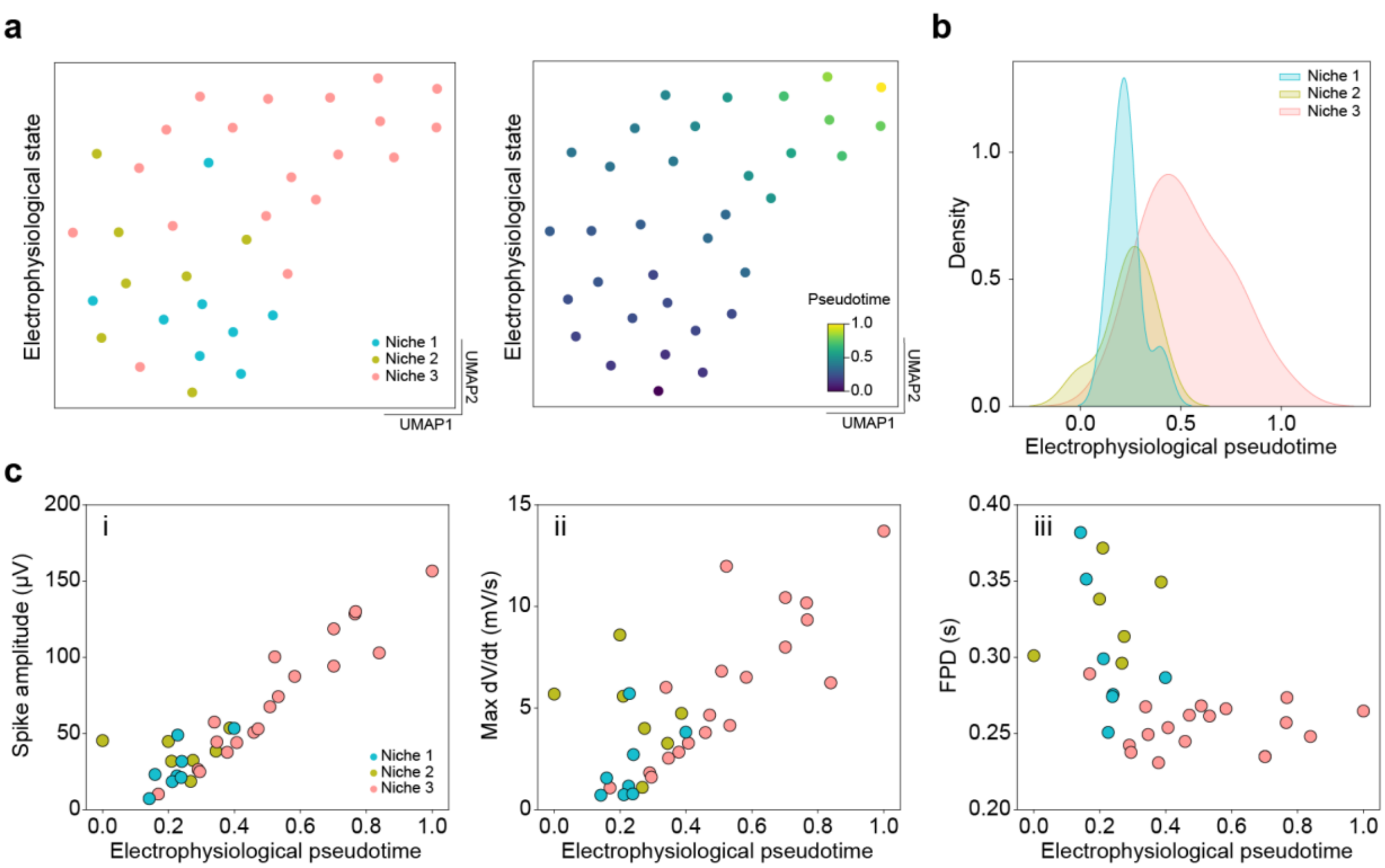

Response Fig. 3-2: Electrophysiological pseudotime computed in PCA space. **a**, UMAP embedding of electrophysiological features from electrically recorded CMs, color-coded by cell niche (i) and electrophysiological pseudotime (ii). **b**, Density plot showing electrophysiological pseudotime distributions

across the three niches. **c**, Scatter plots showing correlations between electrophysiological pseudotime and waveform features: spike amplitude (i), maximum dV/dt (ii), and FPD (iii).

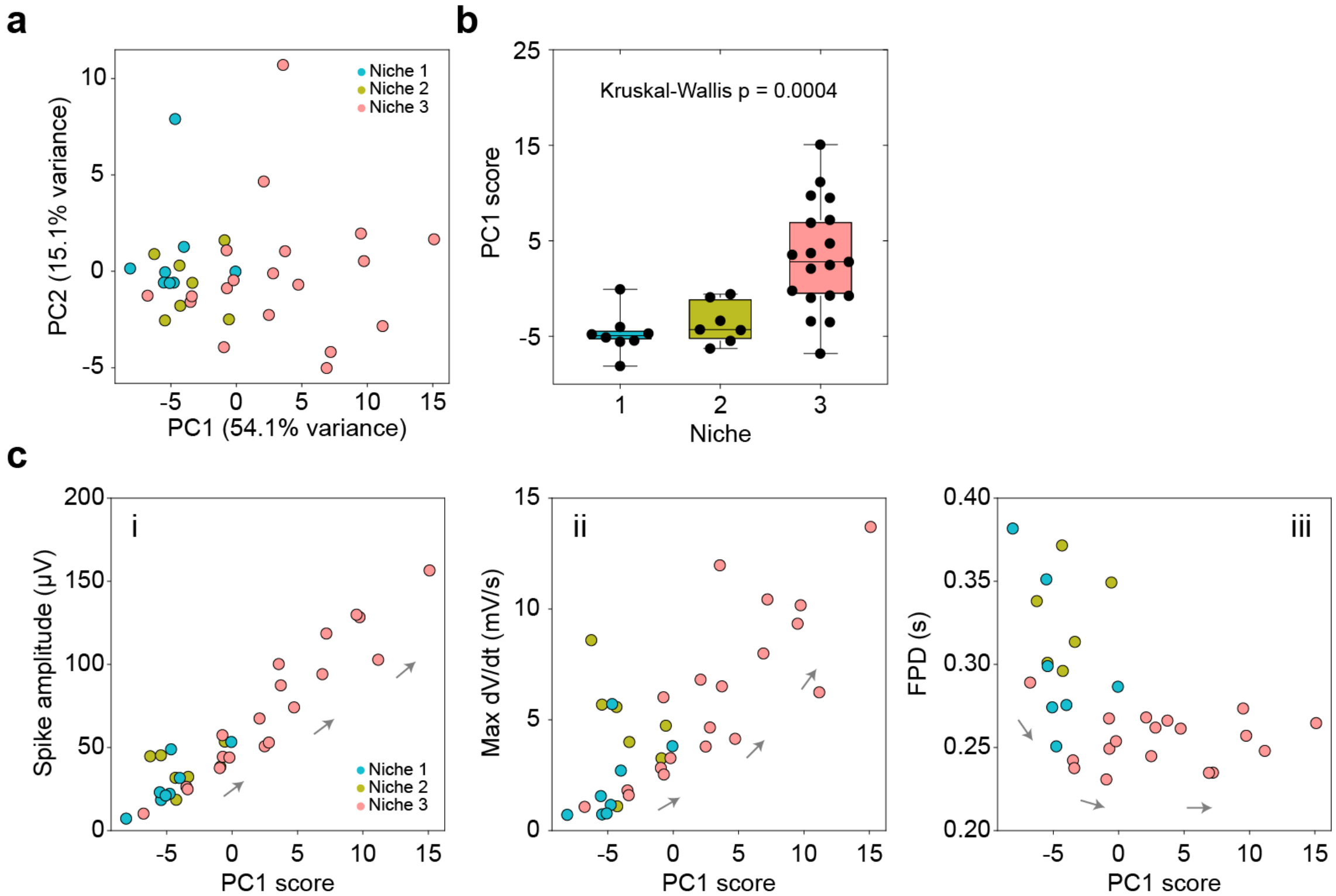

Response Fig. 3-3: Electrophysiological trajectory defined in PCA space. a, PCA embedding of electrophysiological features from electrically recorded CMs, color-coded by cell niche. **b**, Box-and-whisker plot showing the distribution of PC1 scores across the three niches. P-values were calculated from Kruskal-Wallis test. Sample sizes: Niche 1 (n=8), Niche 2 (n=7), Niche 3 (n=19). **c**, Scatter plots showing correlations between PC1 scores and waveform features: spike amplitude (i), maximum dV/dt (ii), and FPD (iii). Each dot represents features from a single electrode, color-coded by niche identity. Higher PC1 scores correspond to increased spike amplitude, greater maximum dV/dt, and shorter FPD of hiPSC-CMs, indicating a progression toward more electrically matured states.

Revision to the manuscript:

We have replaced **Fig. 3m, n** with **Response Fig. 3-1** and **Fig. 4e–g** with **Response Fig. 3-3**, to illustrate pseudotime and trajectory analyses based on PCA-derived embeddings rather than UMAP. Corresponding descriptions have been updated in the Methods section to clarify that pseudotime and clustering analyses were performed in PCA space, with UMAP used exclusively for visualization.

"Cell typing was performed using previously established methods[23,24]. After segmentation and data preprocessing, we computed PCA ("scanpy.tl.pca") and performed Leiden clustering ("scanpy.tl.leiden")[59] on the kNN graph constructed in PCA space ("scanpy.pp.neighbors"). Leiden clusters were annotated based on differential expression of genes associated with cardiomyocyte, endothelial, fibroblast, or pluripotent identities.

Niche analysis. For niche analysis, the neighborhood composition for each segmented cell derived from the in situ sequencing dataset was computed after cell typing. Based on previously published methods, a radius

of 45 microns from the centroid of each cell was used to define the local microenvironment to include ~2 layers of cells from the center[56]. Within this radius, the composition of neighboring cell types was quantified, resulting in a cell-by-cell-type composition matrix. Dimensionality reduction and clustering were performed using the Scanpy package. PCA was applied to the composition matrix, and a kNN graph was computed in PCA space. Leiden clustering was then performed on the resulting kNN graph to identify groups of cells with similar neighborhood compositions. UMAP was used for visualization of the low-dimensional embeddings.

Pseudotime and trajectory analysis. Pseudotime analysis was conducted using the Slingshot package[43] to infer the temporal progression of gene expression or electrophysiological states. For the transcriptomic pseudotime, gene expression profiles of all sequenced CMs were embedded in PCA space, followed by kNN graph construction. Leiden clustering results on kNN graph were used as input labels to guide lineage inference. PCA embeddings and cluster labels were exported to R and processed through Slingshot, which fitted a principal curve to estimate pseudotime trajectories. The resulting pseudotime values were normalized to a [0, 1] range for comparability. Electrically recorded cells were selected after pseudotime calculation and visualized using UMAP. For the electrophysiological dataset, given the limited sample size, PC1, which accounted for 54.1% of total variance, was utilized as a linear progression score to represent electrophysiological state trajectories."

Comment-2: The manuscript lacks sufficient detail regarding the methodology used to create UMAP from electrode waveform data (Fig.3d). While Fig.1b appears to show waveform feature points, no textual explanation accompanies this figure. Additionally, Fig.4a is described as clustering by molecularly defined niche, but the authors should explain what each column in the electrophysiology data represents. This information is crucial for understanding the analysis and interpreting the results.

Response: We thank the reviewer for this valuable comment and agree that additional methodological detail is needed to clarify how the UMAP embeddings and electrophysiological features were generated.

For **Fig. 3d**, the UMAP embedding was generated from the entire representative waveform recorded from each electrode using Python package Scanpy. Aligned waveforms were first averaged to obtain a representative waveform per electrode. We then applied PCA ("*scanpy.tl.pca*") to capture the major sources of variation across waveforms, followed by construction of kNN graph (*"scanpy.pp.neighbors"*) in PCA space. Finally, we computed a UMAP embedding (*"scanpy.tl.umap"*) to generate a low-dimensional representation that preserves local and global waveform relationships. This allowed us to visualize electrophysiological heterogeneity across the tissue by comparing the full waveform signal rather than derived scalar features alone.

For **Fig. 4a**, the electrophysiological data represent 62-dimensional feature vectors derived from representative waveform from each electrode. Each column in the electrophysiology matrix corresponds to one of these 62 downsampled waveform points. These features were produced using a hierarchical median-based downsampling method, adapted from our previous work[3], to retain both global and fine-scale waveform dynamics. Each representative waveform was denoised using PyWavelets and downsampled at two temporal resolutions: (i) 22 "zoomed-out" points sampled across the full waveform to capture global morphology, and (ii) 40 "zoomed-in" points extracted from a 0.1-second window centered on the maximum dV/dt to capture fine-scale depolarization and early repolarization dynamics. The combined 62-point feature vector from each electrode was then used for multimodal data integration and analyses.

Revision to the manuscript:

We have revised Methods section to provide a clearer description of the analysis.

"Electrophysiological signal processing and feature extraction

Further waveform analysis was performed using the Python package Scanpy[53]. Aligned waveforms from each electrode were averaged to generate representative waveforms. For visualization of electrophysiological heterogeneity (Fig. 3d), we performed dimensionality reduction of the representative waveform from each electrode. We first applied Principal Component Analysis (PCA, "scanpy.tl.pca") to capture the major sources of variation across waveforms. We then constructed a k-nearest neighbor (kNN) graph ("scanpy.pp.neighbors") based on the PCA space. Finally, we computed a UMAP embedding ("scanpy.tl.umap") to generate a low-dimensional representation that preserves local and global waveform relationships[54]. This approach enabled visualization of waveform-level differences across electrodes, revealing electrophysiological diversity within the tissue.

Waveform features, including amplitude, maximum dV/dt, and FPD, of the representative waveforms were analyzed using GraphPad Prism. Waveforms that did not exhibit significant FPD peaks were excluded from the FPD analysis.

Electrophysiological features were extracted using a hierarchical median-based downsampling algorithm adapted from previously described methods[24]. Each representative waveform was denoised using PyWavelets (wavelet='db6', level=3, factor=5)[55] and then downsampled at two temporal resolutions. First, the entire waveform was divided into 21 equal-length bins, and the median amplitude within each bin was computed to generate 22 "zoomed-out" representative points capturing the global waveform shape. Next, a 0.1 second high-resolution segment centered on the maximum dV/dt was divided into 39 bins, and the median amplitude in each bin was calculated to generate 40 "zoomed-in" points preserving fine-scale depolarization and early repolarization dynamics. The global and local representations (22 + 40 points) were concatenated to form a 62-dimensional feature vector for each electrode."

Comment-3: The joint analysis of electrophysiology, gene expression, and local cell composition (line 247-250) represents the most significant analytical component of this study. However, the manuscript does not adequately describe how these qualitatively different data types were integrated into a unified analysis framework. Please provide detailed methodology on this critical aspect of the work.

Response: We thank the reviewer for this insightful comment. For joint multimodal analysis, we first standardized each modality independently to ensure comparability across data types. Electrophysiology (62 waveform-derived features), gene expression (254 genes), and local cell composition (6 cell-type proportion features) matrices were each z-score standardized using *"scanpy.pp.scale"* so that differences in scale or units would not bias the integrated analysis. The three standardized matrices were then concatenated column-wise to generate a unified feature matrix (34 samples × 322 features).

We next applied PCA to the combined matrix to derive a shared low-dimensional representation. A kNN graph was constructed in the PCA space, and Leiden clustering was applied to this graph to identify joint multimodal clusters. UMAP was used only for visualization.

Revision to the manuscript:

We have expanded the Methods section to include a detailed description of the joint multimodal analysis framework.

"Joint multimodal analysis framework. To integrate electrophysiology, gene expression, and local cell composition into a unified analytical framework, each dataset was first z-score standardized using the "scanpy.pp.scale" function in Scanpy to normalize feature scales and units. The standardized matrices were then concatenated column-wise, generating a unified feature matrix comprising 322 features (62 electrophysiological features, 254 genes, and 6 compositional features). PCA was performed on this combined matrix to capture shared variance across modalities, followed by UMAP for visualization. Leiden

clustering was applied to the PCA-reduced space to identify cell-state groupings based on integrated multimodal features."

Comment-4: While the comparison of gene expression across cardiomyocyte states includes early marker genes, ion channels, structure genes, and gap junction genes, several key structural genes that best reflect maturation status are missing. Please include data for TNNT2, MYH7 (beta-MHC), MYL2, and TNNI3 in Extended Fig.7, as these are essential for a comprehensive assessment of cardiomyocyte maturation.

Response: We thank the reviewer for this helpful suggestion. As requested, we have now included the expression profiles of key structural maturation genes *TNNT2*, *MYH7 (β-MHC)*, *TNNI3*, and *MYL7* (**Response Fig. 3-4**).

While *MYL2* is a well-established ventricular maturation marker, it was not included in our gene panels. Instead, we included *MYL7* (atrial myosin light chain 2, MLC2a), which performs a similar role but is preferentially expressed in atrial-type cardiomyocytes.

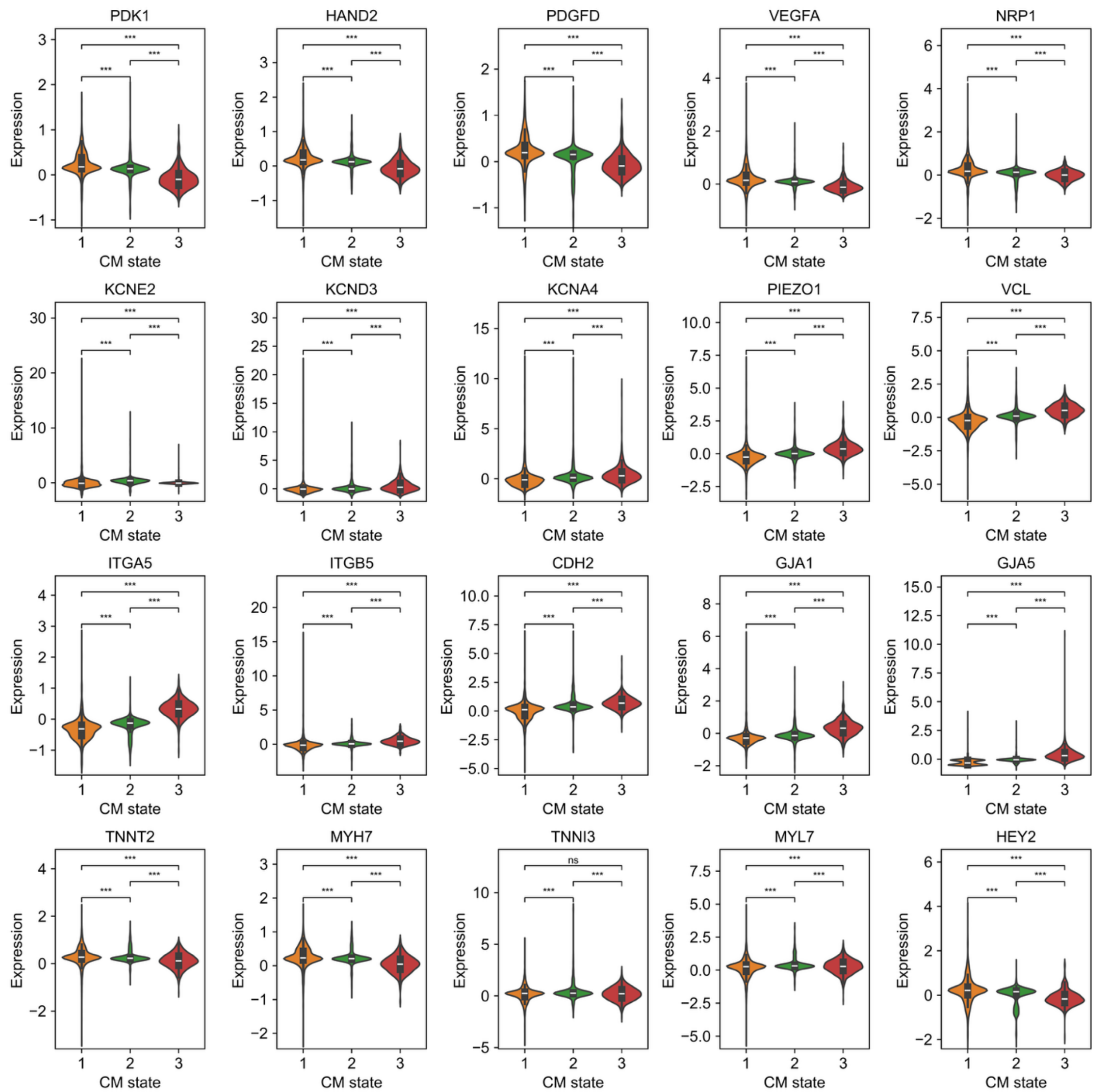

Response Fig. 3-4: Gene expression profiles of different hiPSC-CM states. Violin plots illustrating the expression levels of selected genes across different cardiomyocyte states. Values are normalized, log-transformed (log(x+1)) and scaled to unit variance and zero mean. All pairwise comparisons between cardiomyocyte states were performed using a two-tailed Mann-Whitney test (* $p<0.05$, ** $p<0.01$, *** $p<0.001$, ns: not significant).

Revision to the manuscript:

We have replaced **Supplementary Fig. 9** (formerly Extended Data Fig. 7) with **Response Fig. 3-4**.

Minor Comments

Comment-5: Typos in the text: "silane" should be "saline" (line 360, 361)

"previous published methods" should be "previously published methods" (line 485)

Response: We appreciate the reviewer for pointing out these details. The phrase *"previous published methods"* has been corrected to *"previously published methods"* in the revised manuscript. Regarding *"bind-silane,"* this term was used intentionally to refer to the adhesion promoter (3-glycidoxypropyltrimethoxysilane) employed during device-cell integration.

Comment-6: Issues with figures:

Symbol mismatch in Fig.1c: Gene "n" versus Gene "l"

Compressed/illegible text in legends for Fig.2a, Fig.2c, and Fig.3b, specifically for "TNNT2",

"NKX2.5" and "CD31" Typographical error in Fig.4a legend: "Electrophysiolgical" should be "Electrophysiological" (missing "o")

Response: We thank the reviewer for carefully identifying these figure and labeling issues. The typographical error in **Fig. 4a** (*"Electrophysiolgical" → "Electrophysiological"*) has been corrected. The symbol mismatch in **Fig. 1c** and the compressed or illegible text in the legends of **Fig. 2a**, **Fig. 2c**, and **Fig. 3b** (for *TNNT2*, *NKX2.5*, and *CD31*) have also been corrected as shown below.

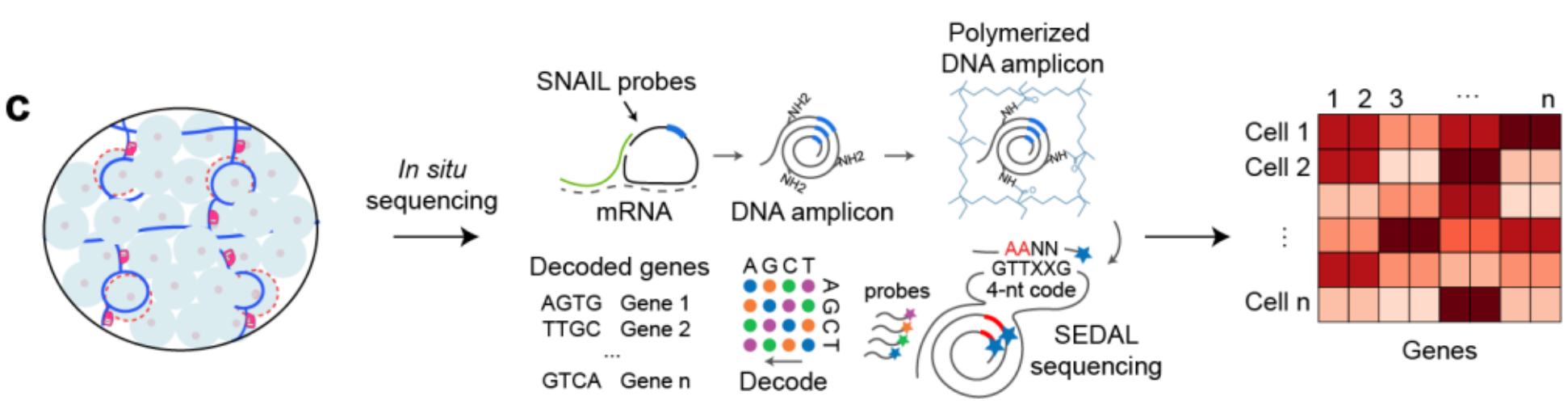

Revised Fig. 1c, *In situ* sequencing generates spatially resolved single-cell gene expression profiles.

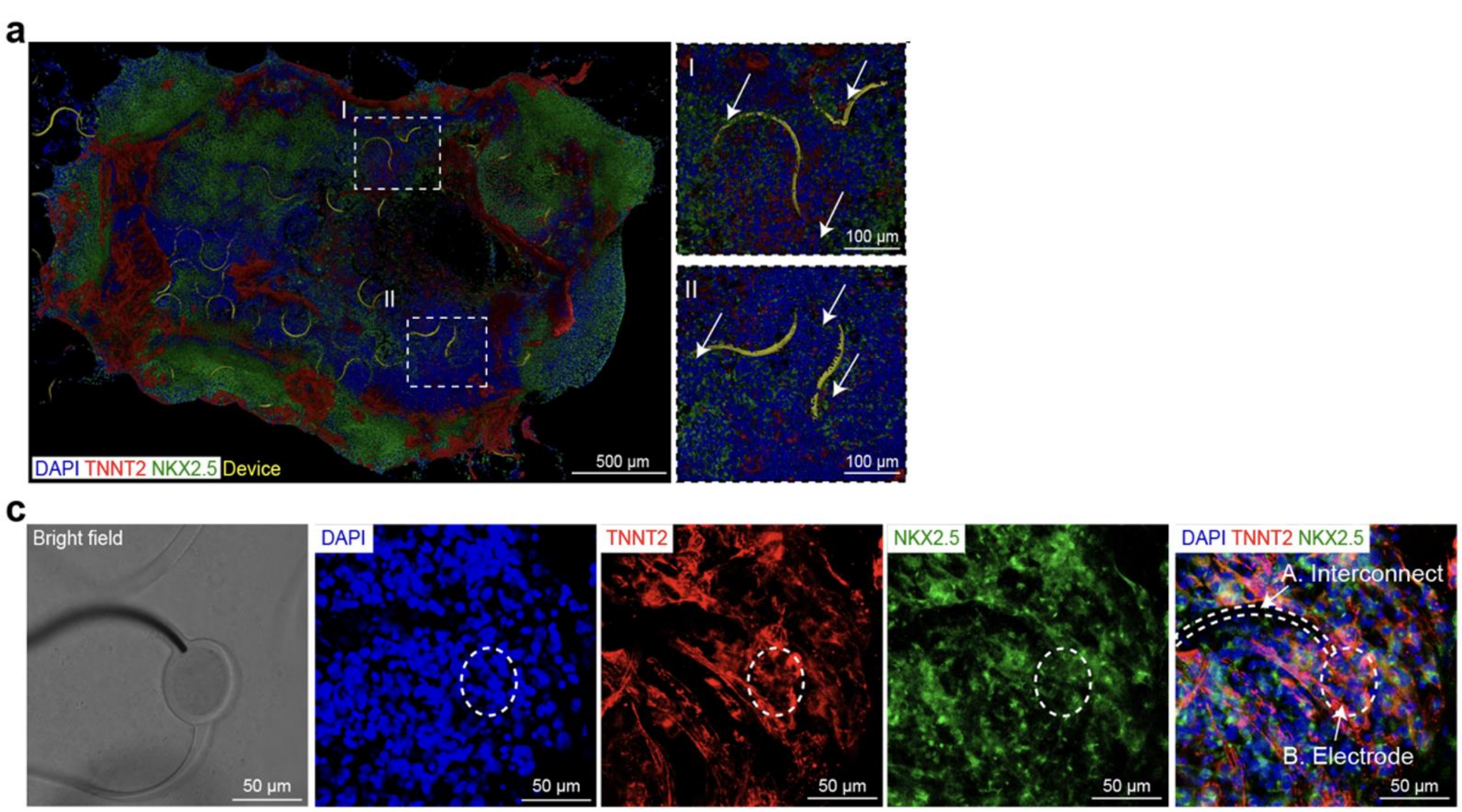

Revised Fig. 2a, Confocal fluorescence reconstruction. Right panels provide zoom-in views of the dashed-box regions in the left panel. White arrows highlight meshes embedded within the organoid. **2c,** BF and confocal fluorescence images of immunohistochemically stained CMs surrounding a representative graphene/PEDOT:PSS electrode. Regions A and B indicate areas over metal interconnect and graphene/PEDOT:PSS electrode, respectively.

b

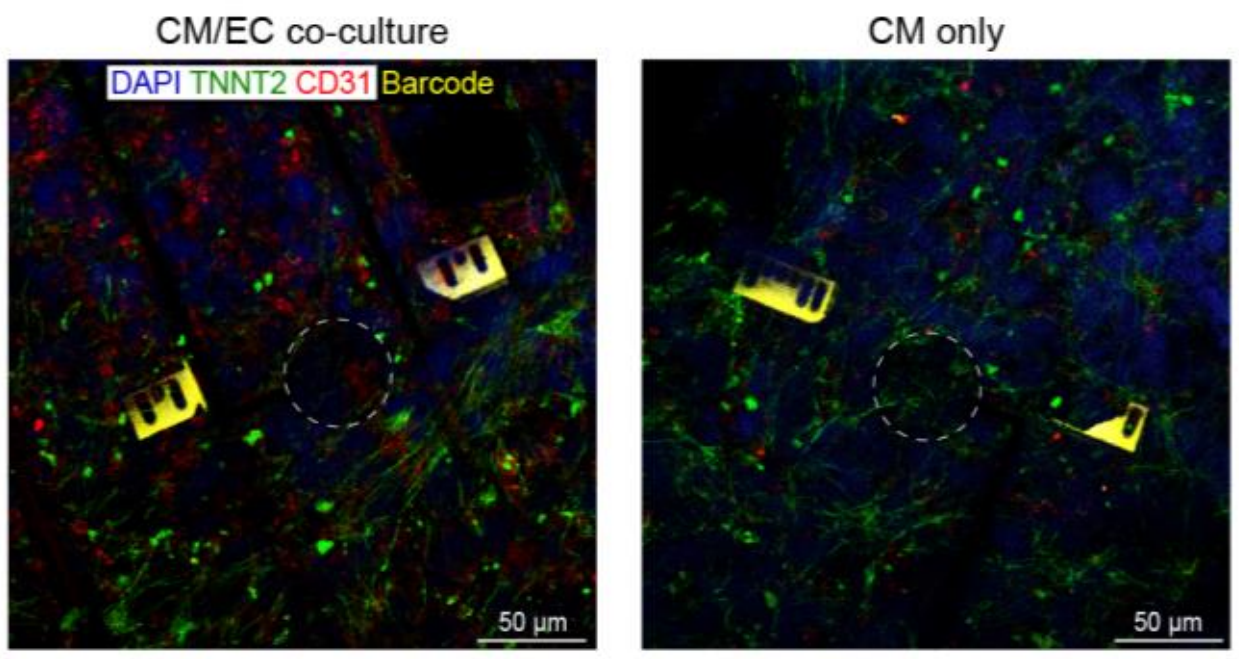

Revised Fig. 3b, Confocal fluorescence images showing immunohistochemically stained cells near electrodes in the CM/EC co-culture region and the CM only region. Images were acquired using identical microscopy settings and processed using consistent display parameters, including min/max intensity, brightness, and contrast.

a

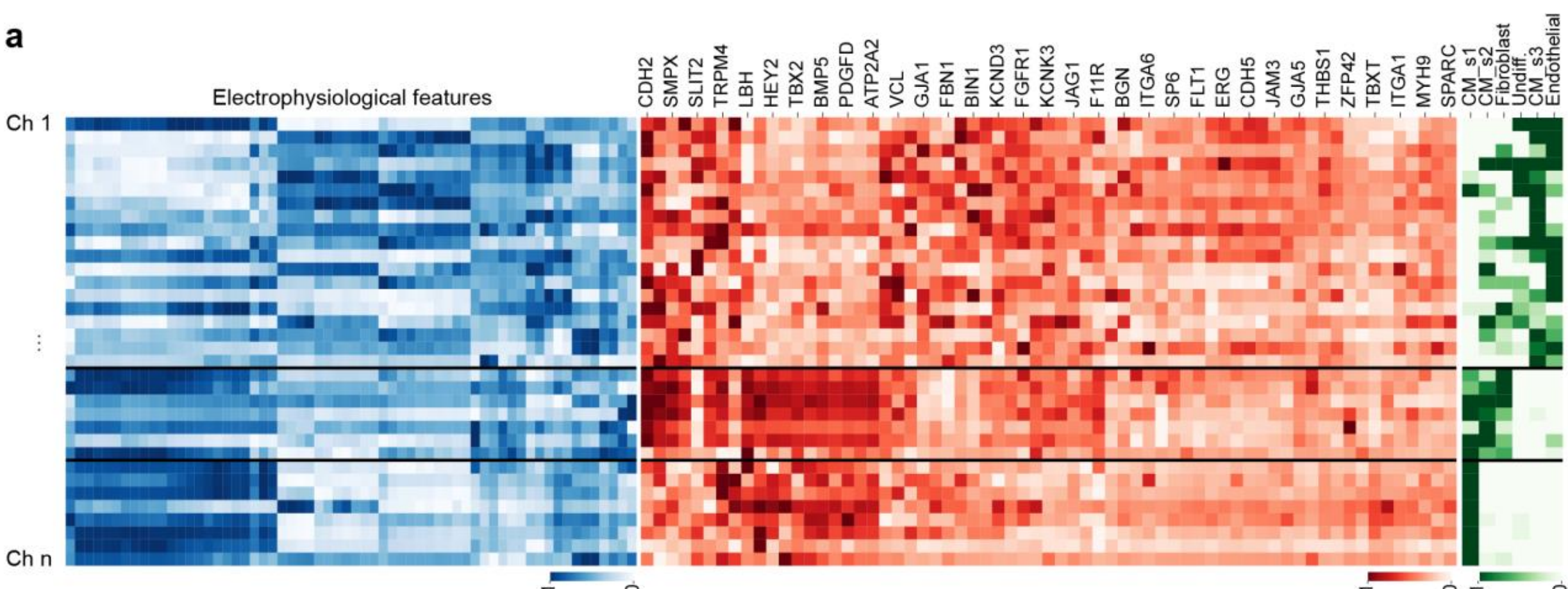

Revised Fig. 4a, Concatenated heatmaps displaying electrophysiological features, selected gene expressions, and local cell compositions of identified cells, grouped by molecularly defined cell niches.

<u>Response to Reviewer #4 Comments</u>

General Comment: This study addresses an important challenge: how can tissue architecture be productively linked to functional outputs? One instance is how spatially distinct transcriptional states give rise to the emergent behavior of electrogenic tissue. In previous work, the authors addressed this by combining tissue-like mesh arrays of barcoded electrodes with spatial transcriptomics to bridge cell electrical and gene expression states. The incompatibility of opaque electrodes with imaging-based transcriptomics, however, precluded analysis of higher-order features, such as cell niches or microenvironments. Here, they use transparent electrodes, made of graphene, that overcome transmittance and impedance issues. This technical innovation now permits studying how cell niches within spatially heterogeneous tissue impact both electrical activity and gene regulation. Application to iPSC-derived cardiac microtissue reveals the composition and arrangement of cell microenvironments that foster functionally mature cardiomyocyte behavior, and impressive feat. Overall, this is an impactful novel technology, with high potential for applications in the study of spatial tissue biology, pathology, and regenerative medicine. The authors nonetheless need to clarify important technical issues, and address biological insights, before it can be considered suitable for publication.

Response: We thank the reviewer for these positive comments and constructive feedback. In response, we clarified key technical aspects of the platform and expanded the biological analyses by refining cell-type characterization and clarifying the relationship between electrophysiological features and cardiomyocyte maturation, thereby strengthening both technical interpretability and biological insights. We provide detailed point-by-point responses below.

Major comments:

Comment-1: The authors allude to improved spatial resolution over their previous platform "in situ electro-sequencing", yet this is not quantified. How much better is it? What is the spatial precision metric that substantiates their assessment? Same with the efficiency of imaging data acquisition. How much more efficient is it?

Response: We thank the reviewer for this thoughtful comment. The primary improvement in spatial precision arises from the transparency of the graphene/PEDOT:PSS electrodes. In our previous *in situ* electro-sequencing platform, the opaque metal electrodes occluded fluorescence signals, creating 10-20 µm blind regions at each recording site. Consequently, the spatial precision of cell-electrode registration was limited by the electrode footprint rather than by the optical system.

In the present platform, the electrodes are optically transparent (>80% transmission across the visible spectrum, **Fig. 1.j**), allowing direct visualization of fluorescently labeled amplicons at the electrode sites without obstruction. Because the electrodes no longer block fluorescence, the spatial precision is determined mainly by the optical resolution of the confocal microscope (typically ~200 nm), rather than by device geometry. This represents approximately an order-of-magnitude improvement in effective spatial resolution relative to the previous opaque-electrode system.

Although refractive-index mismatches can in principle affect imaging, the graphene and PEDOT:PSS layers are significantly thinner than the optical wavelength. As a result, any focal shift or aberration is negligible compared with the microscope's depth of field.

Regarding the efficiency of imaging data acquisition, the prior opaque-electrode platform required manually detaching and flipping the tissue to image cells located on the electrodes. While the absolute time required for this manipulation varies with operator proficiency, the process imposes an unavoidable linear workflow bottleneck: each additional sample required a discrete flipping cycle, scaling the time cost linearly with sample size, with associated risks of misalignment, sample deformation, and handling-induced variability. Due to the imaging depth and molecular probe penetration, this results in the limited ability to image very thin samples, fail to characterize the thick tissue, especially the heterogeneous tissue sample as

described in this manuscript. In contrast, the transparent interface allows imaging directly through the device, eliminating this step entirely. This removes the per-sample handling overhead regardless of user skill, greatly improving throughput and reproducibility, particularly when processing multiple samples.

Together, these advances—removal of electrode-induced blind regions, diffraction-limited spatial precision, and elimination of the tissue-flipping workflow—quantitatively and qualitatively improve both the spatial resolution and the efficiency of imaging relative to the previous platform.

Revision to the manuscript:

We discussed additional detail of linear bottleneck of process/ reproducibility in the manuscript.

"The previous approach, which relied on opaque metal electrodes, required additional procedures such as detaching the tissue/electronics hybrid, flipping the sample, and transferring it to another plate for imaging electrically recorded cells (Supplementary Fig. 3d–f). These introduced substantial handling variability and often disrupted the native cell microenvironment. In addition, the sequential nature of this workflow created a linear bottleneck, where experimental time scaled directly with the number of samples. The orientation of electrodes further constrained image acquisition of recorded cells. By incorporating transparent graphene/PEDOT:PSS electrodes, we eliminated these additional steps and enabled both electrophysiological recordings and imaging on a single platform, while preserving the original spatial localization of the tissue (Supplementary Fig. 3g–i). The removal of per-sample handling, combined with unobstructed optical access to recorded cells regardless of electrode orientation, facilitates high-throughput multimodal analyses within 3D tissues."

Comment-2: The robustness of the technical innovation isn't properly quantified either. Is it possible to quantify what is possible now, that wasn't possible before, in terms of robustness of outcomes?

Response: We thank the reviewer for this important question. The robustness of the technical innovation and the resulting outcomes were affected by nanoelectronics device fabrication yield, sensor electrochemical stability, and longitudinal recording fidelity. Here, we provide quantification of the yield for each step.

First, we quantify the yield and reproducibility of the device fabrication process. The reproducibility of fabricated devices is shown in **Supplementary Fig. 2g**. Across five independently fabricated samples, each with a 64-electrode design, we observed a high active channel yield of 0.95 ± 0.046 following fabrication and release from the substrate for tissue integration. To assess reproducibility of electrode impedance, we measured the mean impedance of active electrodes within each device in 1X PBS. As shown in **Supplementary Fig. 2g**, the five devices exhibited consistent average impedance values (114.4 ± 18.4 kΩ, n=5 devices), confirming that the fabrication process reliably produces devices with repeatable electrical characteristics.

We further characterized the stability of the sensors in the biological environment by tracking the impedance of electrodes in 1X PBS over 60 days. Among the 7 testing samples, the impedance value of all channels (100%) remain stable throughout the testing period (**Supplementary Fig. 2h**). Additionally, Raman spectra of the graphene/PEDOT:PSS electrodes collected before and after the 60-day incubation (**Supplementary Fig. 2i**) showed unchanged characteristic peaks, indicating that the functional materials remain chemically stable and do not degrade or delaminate during long-term immersion.

With respect to recording robustness, we assessed signal stability in actual tissue integration. Over a 2-week period, 12 of 16 monitored channels (75%) maintained high-quality, sortable waveforms (**Fig. 2e–j**). The remaining four channels continued to record electrically active signals (indicating device integrity) but were excluded from analysis due to lower signal-to-noise ratios. This variability is expected at tissue-electrode

interfaces and is likely due to incomplete or inconsistent cell-electrode coupling rather than device malfunction.

The key distinction lies in the optical modality. Opaque metal electrodes block transmitted fluorescence imaging directly through the electrode region, preventing direct optical access to recorded cells without additional sample manipulation. In contrast, our platform enables direct imaging of the recorded cells through the transparent electrode sites. As described in our response to Comment-9, we performed a quantitative image resolution analysis (**Supplementary Fig. 4**), which demonstrated no detectable loss of imaging resolution within the electrode region compared to adjacent areas.

Collectively, these quantitative assessments demonstrate that our platform provides a robust and reliable tool for multimodal investigation.

Comment-3: . That transparent electrodes lower the barrier to optical imaging applications is self-evident. Yet the pair of E-barcodes positioned near each electrode occupy a similar total area than the electrode itself. Don't they obstruct the optics needed to image the neighborhood of the recorded cell, akin to opaque electrodes?

Response: We thank the reviewer for this observation. While the E-barcodes occupy an area comparable to the electrode, their impact on imaging is fundamentally different from that of opaque metal electrodes. Most importantly, the barcodes are placed laterally adjacent to the recording site rather than underneath it. This spatial offset ensures that the electrode area remains completely unobstructed, allowing high-resolution imaging of the recorded cell and its immediate neighbors.

Furthermore, the barcode material does not block light in the same manner as metal pads. Whereas metal electrodes block light across the entire spectrum, the barcode fluorescence is limited to specific excitation and emission wavelengths (e.g., Rhodamine) and therefore does not interfere with imaging in other channels.

Finally, we note that the current barcode dimensions were chosen to ensure robust fabrication and alignment for this study. With higher-resolution lithography or modified encoding strategies, the barcode dimensions can be substantially reduced if needed.

Revision to the manuscript:

To clarify the position and design of the barcodes, we have added the following description to the figure caption.

*"**Supplementary Fig. 1f**, Design layout of E-barcodes positioned laterally adjacent to electrodes for labeling. The barcode geometry and footprint can be adapted through alternative encoding strategies and lithographic parameters."*

Comment-4: How are single cell signals registered? It appears that many cells can interface with the same electrode, including of the same cell type.

Response: We thank the reviewer for this important question. We agree that multiple nearby cells can interface with the same electrode. However, extracellular recordings are dominated by the cell-electrode coupling, which is determined by interfacial impedance and strongly influenced by the seal resistance and junctional membrane resistance between the cell membrane and the electrode (**Response Fig. 4-1**)[5]. When the effective cell-electrode contact area is small, the junctional resistance remains high, and the contribution of that cell becomes negligible, resulting in a signal indistinguishable from background noise. Thus, although several cells may be in proximity to an electrode, typically only the closest or best-coupled cell contributes meaningfully to the recorded extracellular signal.

To register cells to electrodes, we computed the distance between the centroid of each electrode and surrounding segmented cells. Cells exhibiting the shortest distances to a given electrode were designated as recorded cells, representing the most likely electrophysiological source based on spatial proximity and expected biophysical coupling.

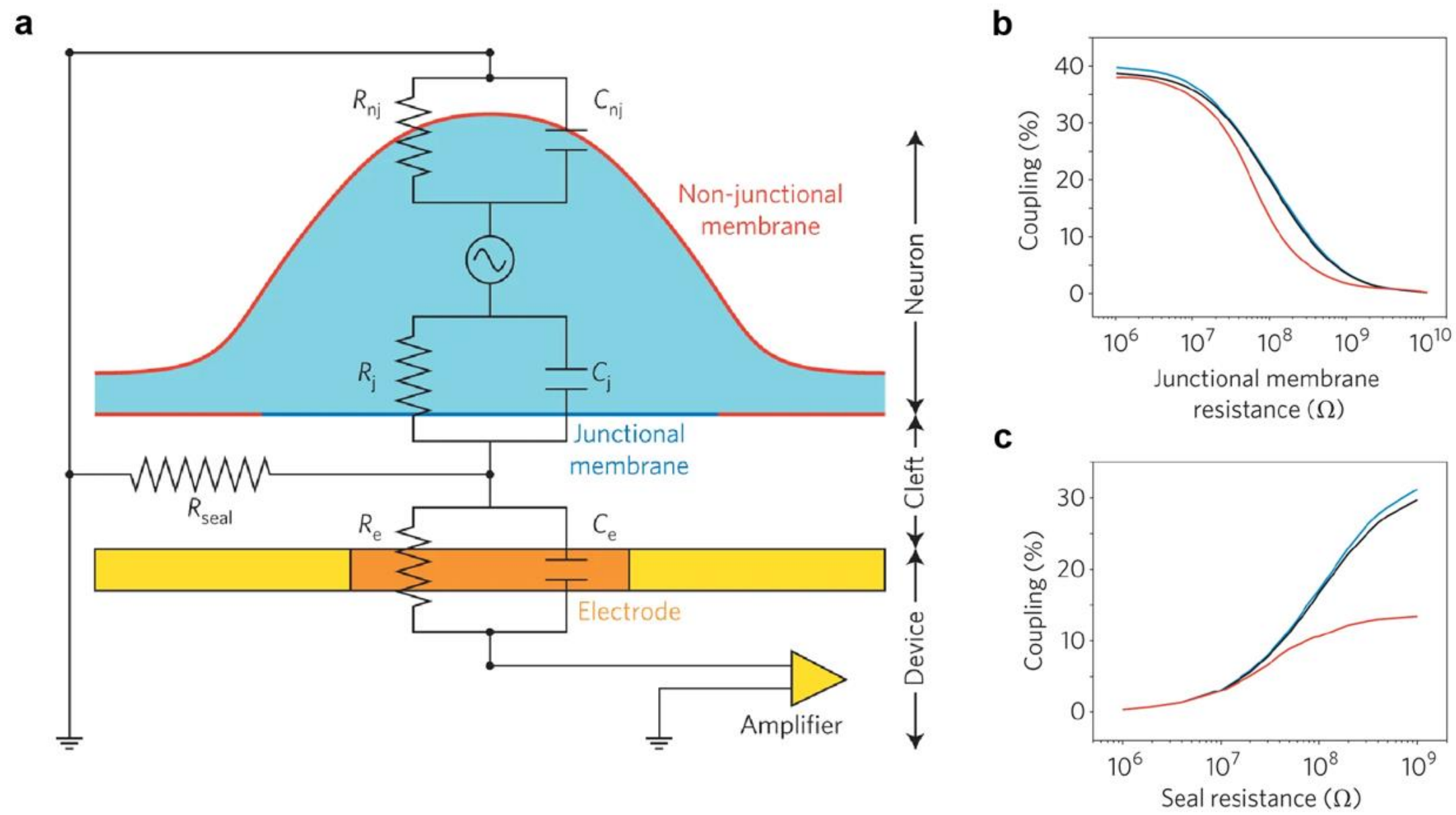

Response Fig. 4-1: Electrical circuit model of the electrode-cell interface. a, Schematic illustrating the passive equivalent circuit describing the electrical coupling between the electrode and a nearby cell. **b-c**, Coupling coefficient as a function of junctional membrane resistance (b) and seal resistance (c). Blue, black, and red curves represent low-frequency (1-50 Hz), medium-frequency (100 Hz) and high-frequency (500-1,000 Hz) signals, respectively. (Image adapted from[5])

Revision to the manuscript:

We have added clarification of the procedure used to register electrically recorded cells in Method section.

"Electrode segmentation and recorded cell identification. Device components, including the graphene/PEDOT:PSS electrode, SU-8 passivation layers, and interconnect cable, were visualized using reflection mode imaging on a Leica TCS SP8 microscope. The reflection channel data underwent maximum intensity projection (MIP) onto the x-y plane. Electrode segmentation was performed using the Segment Anything Model (SAM, version sam_vit_h_4b8939) by Meta[62], with manual annotation of foreground and background regions to generate an electrode mask. This mask was then applied to the 3D reflection images to isolate the electrodes, which were subsequently converted to 8-bit (0-255) grayscale images and reconstructed as point clouds. A 3D convex hull was generated from the reconstructed electrode point clouds to define the electrode recording volume. To identify recorded cells, the distances between the centroid of each segmented cell and the electrode volume were calculated. Cells located within the convex hull and exhibiting the shortest distances to the electrode centroid were designated as the recorded cells, representing the most likely electrophysiological sources based on spatial proximity and expected biophysical coupling."

Comment-5: Given that "unidentified" cells are a major determinant of what makes niche 3 different, they must be characterized. What are their markers**?** The markers that distinguish each cell type should be presented, and best-guess predictions of cell identity should be provided.

Response: We thank the reviewer for this insightful comment. We agree that characterizing the "unidentified" population is essential for a complete understanding of the cellular niches, particularly Niche 3, where they are most prevalent. To address this, we showed the differential gene expression profiles of all identified cell clusters using the specific gene panel designed for this study. We have generated a heatmap (**Response Fig. 4-2**) displaying the mean expression of key marker genes across all cell types to substantiate their identities.

The heatmap confirms the molecular identities of the major cell populations based on canonical markers included in our panel: Cardiomyocytes (CM_s1, s2, s3): All subtypes express robust levels of sarcomere genes (*MYL7*, *MYH6*, *MYH7*, *ACTN2*, *RYR2*), but with different expression patterns. Endothelial cells: Distinctly marked by *PECAM1* (CD31) and *CDH5* (VE-Cadherin). Fibroblasts: Characterized by high expression of extracellular matrix genes, specifically *COL1A1* and *COL3A1*.

For the population previously labeled "unidentified" lacks the structural markers of definitive CMs, ECs, or fibroblasts. However, our analysis reveals that they exhibit strong expressions of *SOX2* and *ZFP42* (REX1), which are established markers of pluripotency, as well as *EOMES*, a marker of early mesoderm differentiation. Based on this specific molecular signature, we identify these cells as residual undifferentiated iPSCs or early mesodermal progenitors that have not fully committed to a specific somatic lineage fate.

The exclusive presence in the co-culture region suggests that the ECs provide a supportive microenvironment that favors the survival of these residual progenitors. It is well-established that endothelial cells secrete specific factors that can maintain the homeostasis, survival, and self-renewal of resident stem and progenitor cells[6,7]. We hypothesize that while small numbers of residual progenitors were likely to present initially, they were only able to persist in the co-culture region due to these survival signals provided by the neighboring ECs, whereas they failed to survive in the CM-only region lacking this support.

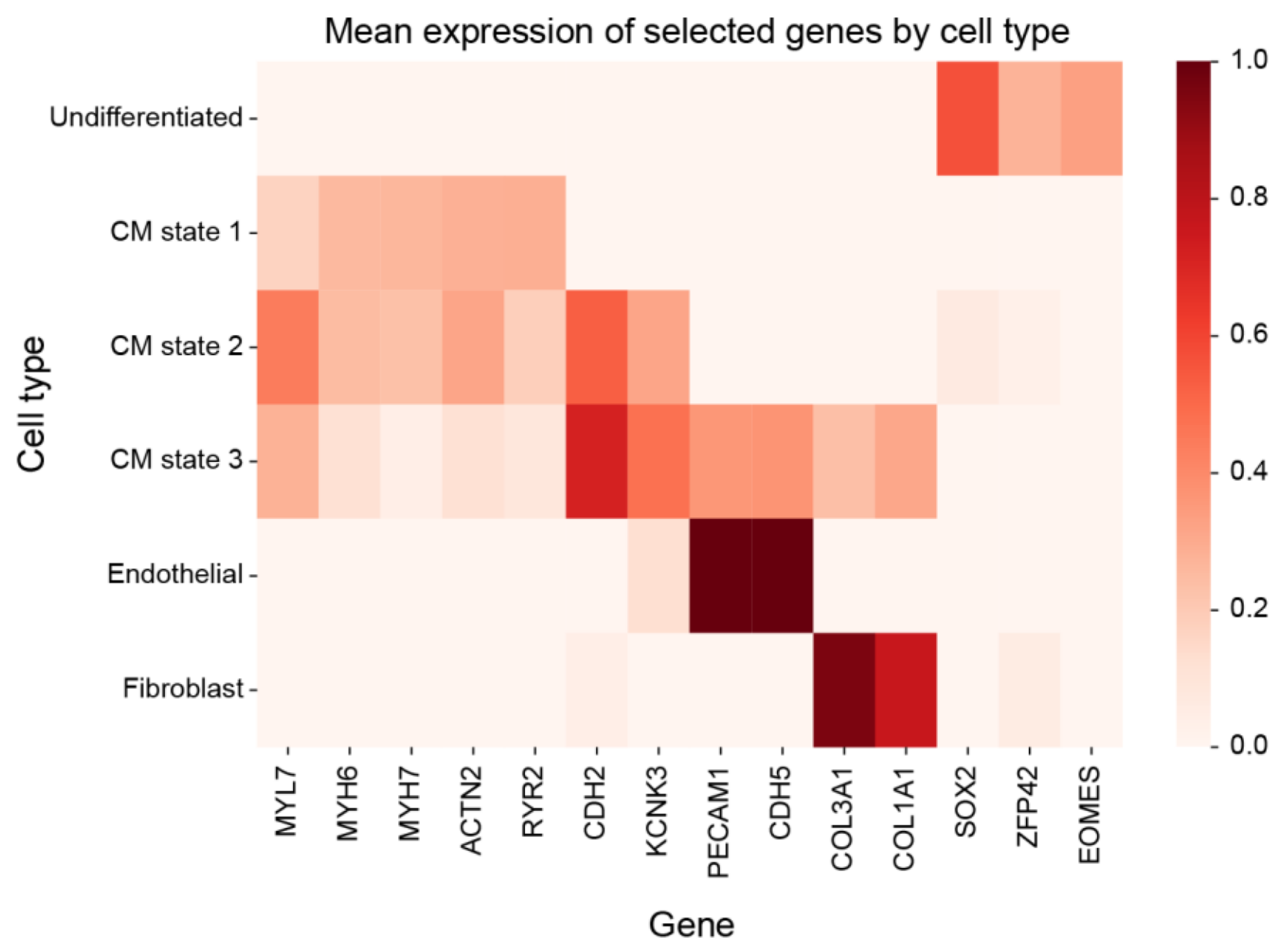

Response Fig. 4-2: Expression profiles of key marker genes for each cell type in CM/EC co-culture system. Color represents log1p-transformed and z-scored data.

Revision to the Manuscript:

We have incorporated the **Response Fig. 4-2** into the Supplementary Information as **Supplementary Fig. 8** to present markers for each cell type and state and discussed results in the Results and Methods section.

"In situ sequencing identified multiple CM states as well as ECs and fibroblasts, annotated using canonical lineage markers (e.g., MYL7, MYH6, ACTN2, RYR2 for CMs; PECAM1 (CD31) and CDH5 for ECs; COL1A1 and COL3A1 for fibroblasts), along with a small population of undifferentiated or progenitor-like cells (Fig. 3h, Supplementary Fig. 8)."

"CM clusters were marked by MYL7, MYH6, ACTN2, and RYR2, and were further resolved into three subpopulations. Endothelial cells were identified by PECAM1 (CD31) and CDH5, while fibroblasts were defined by COL1A1 and COL3A1. An additional "Unidentified" cluster appeared was annotated as residual undifferentiated iPSCs or early primitive progenitors based on high expression of SOX2, ZFP42 (Rex1), and EOMES, together with minimal expression of other lineage-specific markers."

Comment-6: The ratio of endothelial to fibroblast cells seems another distinctive niche 3 feature. Is it possible to regress this ratio against transcriptional /electrophysiological /joint pseudotime across all niches? What should be the most desirable ratio for future microtissue design?

Response: We thank the reviewer for this thoughtful suggestion. We agree that analyzing the non-myocyte composition could provide additional insight into how the microenvironment influences electrophysiological and molecular maturation. We performed regression analyses using individual endothelial and fibroblast densities rather than their ratio, as shown in **Response Fig. 4-3**.

The correlations between these cellular proportions and the electrophysiological, transcriptional, or joint trajectory metrics were weak ($R^2 < 0.3$), suggesting that within the range of cell compositions tested, the precise cellular ratio is not a dominant determinant of cardiomyocyte state. These results suggest that establishing the multicellular niche—ensuring the presence of both support cell types—is likely more critical than fine-tuning their specific ratio for more desirable microtissue design.

We agree that explicitly examining the cell composition ratio and extending this analysis to broader compositional or multicellular interactions will be an interesting direction for future work, particularly as we apply *in situ* graphene-seq to more complex and tunable tissue models.

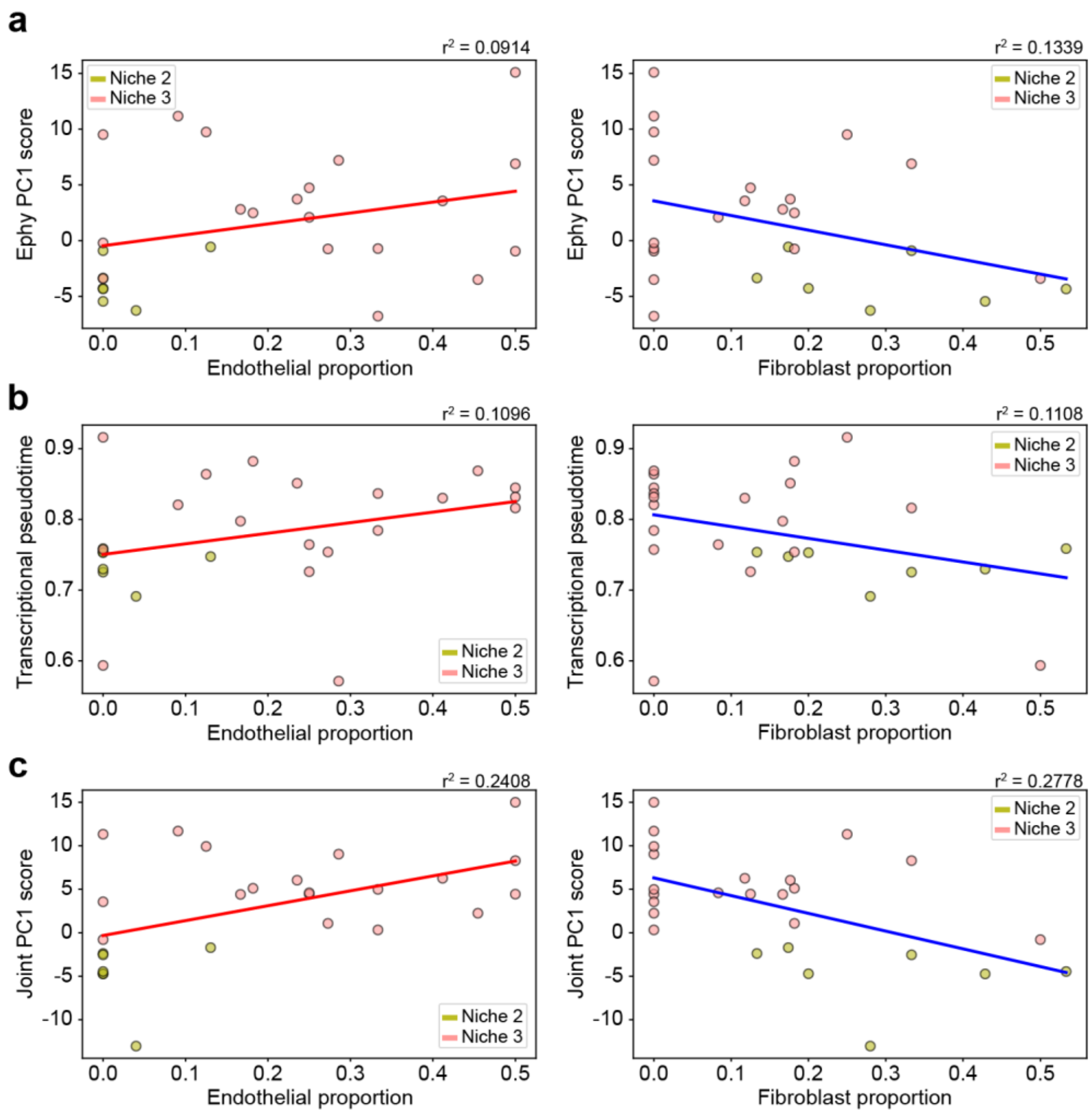

Response Fig. 4-3: Linear regression analyses between cell-type composition and multimodal trajectory metrics. a, Relationship between endothelial (left) and fibroblast (right) proportions and electrophysiological trajectory (PC1 score). **b**, Relationship between endothelial (left) and fibroblast (right) proportions and transcriptional pseudotime. **c**, Relationship between endothelial (left) and fibroblast (right) proportions and joint trajectory (PC1 score).

Revision to the manuscript:

We have added this point to the discussion as a direction for future work.

"Longitudinal multimodal datasets generated by in situ graphene-seq will also be well suited for artificial intelligence (AI)-driven analyses—which have already demonstrated strong capabilities in extracting biological and biomedical insights from high-dimensional data[48,49,50]. Beyond the proof-of-concept demonstration in this manuscript, such approaches may enable prediction of molecular states from electrical dynamics, early detection of niche-dependent maturation, or elucidation of how multicellular composition, including support cell densities, influences electrophysiological and molecular maturation, potentially providing quantitative guidance for microtissue design."

Comment-7: Is predictive inference possible – i.e. predicting molecular / functional state from cell niche?

Response: We thank the reviewer for this insightful suggestion. To explore whether predictive inference is possible, we trained random forest classifiers using either molecular or electrophysiological features to predict the spatial niche labels.

For the molecular dataset, we trained a model using all sequenced cardiomyocytes with an 80/20 train-test split. The model achieved 98.24% accuracy on the held-out test set (**Response Fig. 4-4a**). Examination of feature importances revealed a set of genes with strong discriminatory power across niches (**Response Fig. 4-4b**).

We next applied the same approach to the electrophysiological dataset. Given the limited sample size (n=34), we used Leave-One-Out Cross-Validation (LOOCV) to obtain estimate of generalization performance. The classifier correctly predicted the niche for 82.35% of cells (28 of 34), as summarized in the confusion matrix (**Response Fig. 4-4c**).

Together, these results suggest that both molecular and electrophysiological profiles encode information to infer a cell's spatial niche, supporting the potential for predictive inference between functional and molecular states.

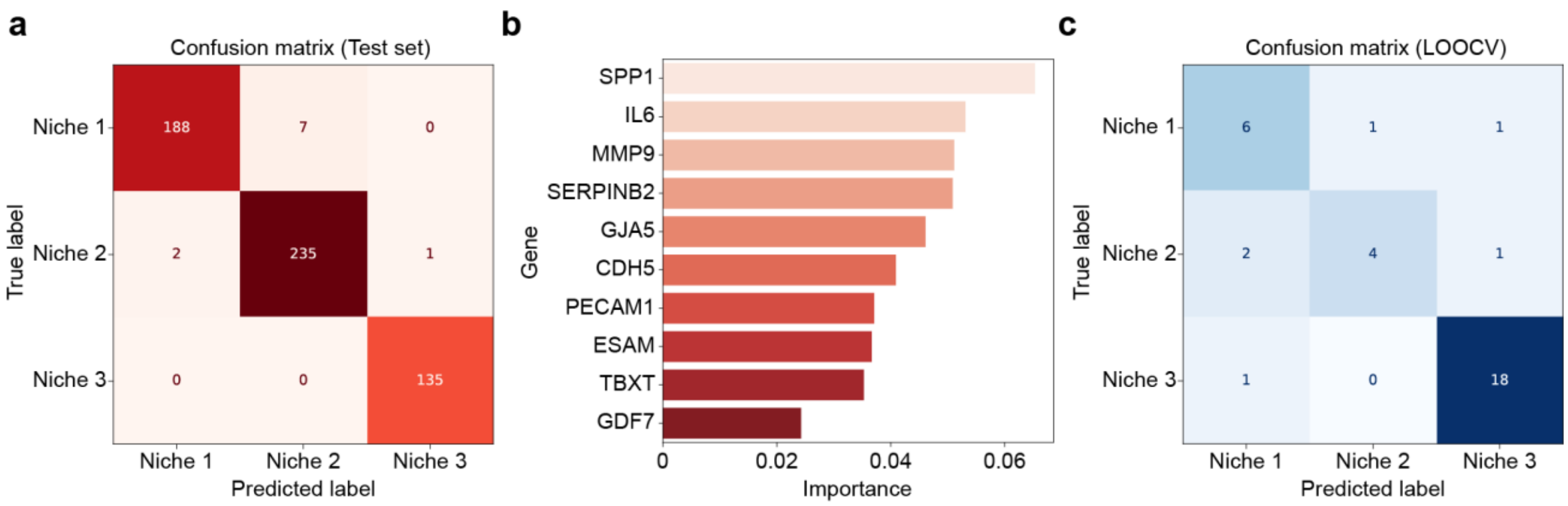

Response Fig. 4-4: Predictive inference of spatial niche from molecular and electrophysiological features. a, Confusion matrix of the random forest classifier trained on gene expression data to predict spatial niche identity of cardiomyocytes, evaluated on a held-out test set (80/20 split). **b**, Ranked feature importance of the top genes contributing to niche prediction. **c**, Confusion matrix of the random forest classifier trained on electrophysiological features, evaluated using leave-one-out cross-validation (LOOCV).

Revision to the manuscript:

We have incorporated **Response Fig. 4-4** into the Supplementary Information as **Supplementary Fig. 10** and discussed the results.

"Beyond descriptive analysis, this multimodal platform potentially enables cross-modal inference between molecular and electrophysiological domains. To explore this capability, we performed proof-of-concept predictive inference of spatial niche identity using either molecular or electrophysiological features (Methods). A random forest classifier trained on gene expression data achieved 98.24% accuracy on a held-out test set (Supplementary Fig. 10a,b). Using electrophysiological features alone, the classifier predicted niche identity with 82.35% accuracy (Supplementary Fig. 10c). Together, these results suggest that electrophysiological recordings encode information associated with molecularly defined spatial niches, highlighting the potential for cross-modal inference enabled by this platform."

Minor comments:

Comment-8: Light transmittance is measured to be less than 85% in the optimized graphene + PEDOT:PSS electrodes. Is this due to decreased light absorption, scattering, or diffraction, and if so can this improved by improving material purity or patterning?

Response: We thank the reviewer for this insightful comment. The observed decrease in transmittance after PEDOT:PSS deposition is primarily attributable to intrinsic absorption of PEDOT:PSS across the visible spectrum, particularly in the red region[8,9]. Light scattering associated with the morphology of electrochemically deposited PEDOT:PSS films may also contribute to optical losses.

Our optimized films achieved greater than 80% transmittance across the visible spectrum, which is compatible with standard bioimaging modalities. Nevertheless, we agree that further improvements are possible. Potential strategies include increasing material purity, adjusting the PEDOT:PSS formulation, tuning deposition parameters to better control film thickness and morphology. Alternative approaches, such as spin-coating PEDOT:PSS and patterning it only at electrode sites, may also improve overall transparency, although compatibility with downstream fabrication steps would need to be carefully evaluated.

Revision to the manuscript:

We have added clarification regarding the trade-off introduced by PEDOT:PSS deposition.

"This process introduces an inherent trade-off between optical transparency and electrochemical performance: increasing PEDOT:PSS deposition lowers impedance but decreases optical transmittance due to increased absorption associated with thicker PEDOT:PSS films[31,32]*."*

Comment-9: What is the difference in imaging resolution for cells behind vs. in front of the transparent electrodes? How do both compare with cells outside of the electrode perimeter?

Response: We thank the reviewer for this important comment. To quantitatively assess the impact of the transparent electrodes on imaging resolution, we performed a comparative analysis of edge sharpness (Sobel gradient magnitude) for DAPI-stained nuclei. We analyzed nuclei in three distinct regions: (i) cells behind the electrode, (ii) cells above (in front of) the electrode, and (iii) control cells located outside the electrode perimeter. For each condition, we calculated the edge sharpness ratio (ROI / outside) across five independent regions.

As shown in **Response Fig. 4-5**, we observed no detectable loss of imaging resolution. Cells cultured behind the electrode retained 99% of the edge sharpness observed in control regions (ratio: 0.99 ± 0.04), while cells cultured above the electrode showed a similarly high ratio of 0.96 ± 0.05. These results indicate that the transparent electrode does not measurably degrade imaging resolution.

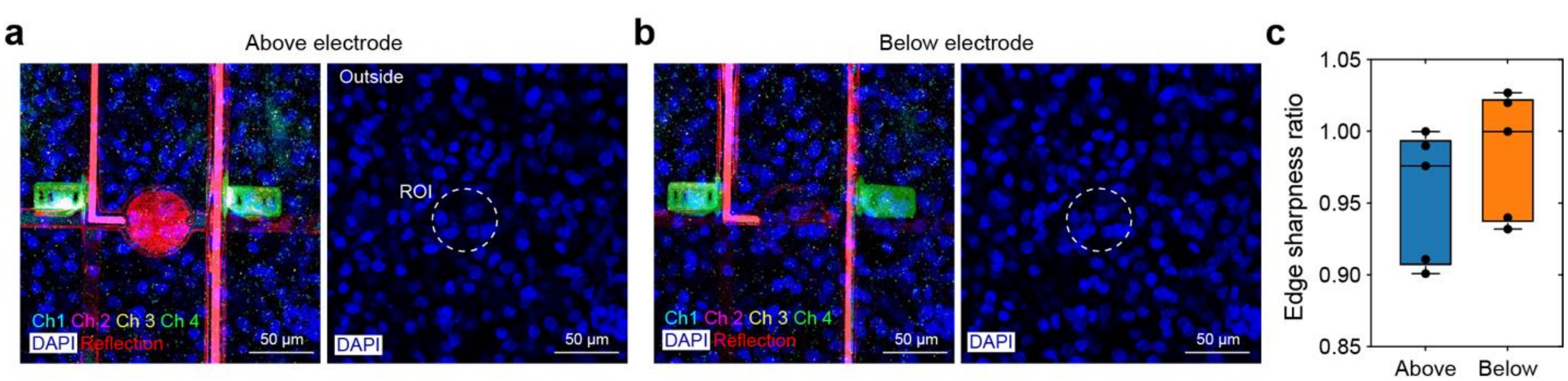

Response Fig. 4-5: Optical accessibility enabled by transparent electrodes. a–b, Representative confocal images of cells integrated with the transparent electrode array. Left panels show the composite channel view, including reflection mode (red) to verify electrode location relative to the Region of Interest

(ROI). Right panels show the corresponding DAPI channel used for image resolution analysis. **a**, Cells above (in front of) the electrode. **b**, Cells below (behind) the electrode. **c**, Quantification of imaging resolution. Edge sharpness (Sobel gradient magnitude) of DAPI-stained nuclei was measured within the electrode region (ROI) and normalized to nuclei in adjacent region outside the electrode perimeter. All images were acquired using identical confocal settings (n=5 independent electrodes).

Revision to the manuscript:

We have incorporated **Response Fig. 4-5** into the Supplementary Information as **Supplementary Fig. 4** and added the following statement to the main text.

"We integrated cardiac microtissues with stretchable and transparent mesh electronics to obtain extracellular recordings, which were processed to extract electrophysiological features (Methods). Immediately after recordings, the same tissue/electronics hybrid was fixed and processed for in situ complementary DNA (cDNA) library preparation and sequencing, enabling spatially resolved transcriptomic profiling in situ (Methods). Consistent with the optical transparency of the electrodes, no detectable loss of imaging resolution was observed for cells cultured above or below the electrodes compared with regions outside the electrode perimeter (Supplementary Fig. 4)."

Comment-10: The CM-only and CM/EC co-culture within the same device is creative – do CMs across the two different regions beat synchronously? This is not quantified either within or across regions.

Response: We thank the reviewer for this insightful comment. The cardiomyocytes (CMs) across the two regions beat in a coordinated manner with a consistent delay. As shown in the representative raw traces (**Response Fig. 4-6**), rhythmic depolarizations occur synchronously within each region, with a consistent delay between the CM/EC and CM-only regions.

To quantitatively compare beating parameters, we analyzed signals from channels corresponding to the first and last activation events in each region. All channels exhibited identical mean beating rates (34.6 bpm) and inter-beat intervals (1.732 ± 0.05 s). Within-region delays (defined as the time difference between the first and last peaks within region) were 0.194 ± 0.002s for CM/EC co-culture region and 0.387±0.003s for the CM-only region. The cross-region delay, measured from the last peak in the CM/EC region to the first peak in CM region, was 0.606 ± 0.006s. Beat-to-beat inter-beat intervals of both regions increased gradually but in parallel over time, indicating a shared rhythm and functional coupling. These results suggest that, although the two regions maintain distinct spatial organization, they remain electrically connected across the interface, consistent with the intended design of a spatially heterogeneous yet integrated tissue model.

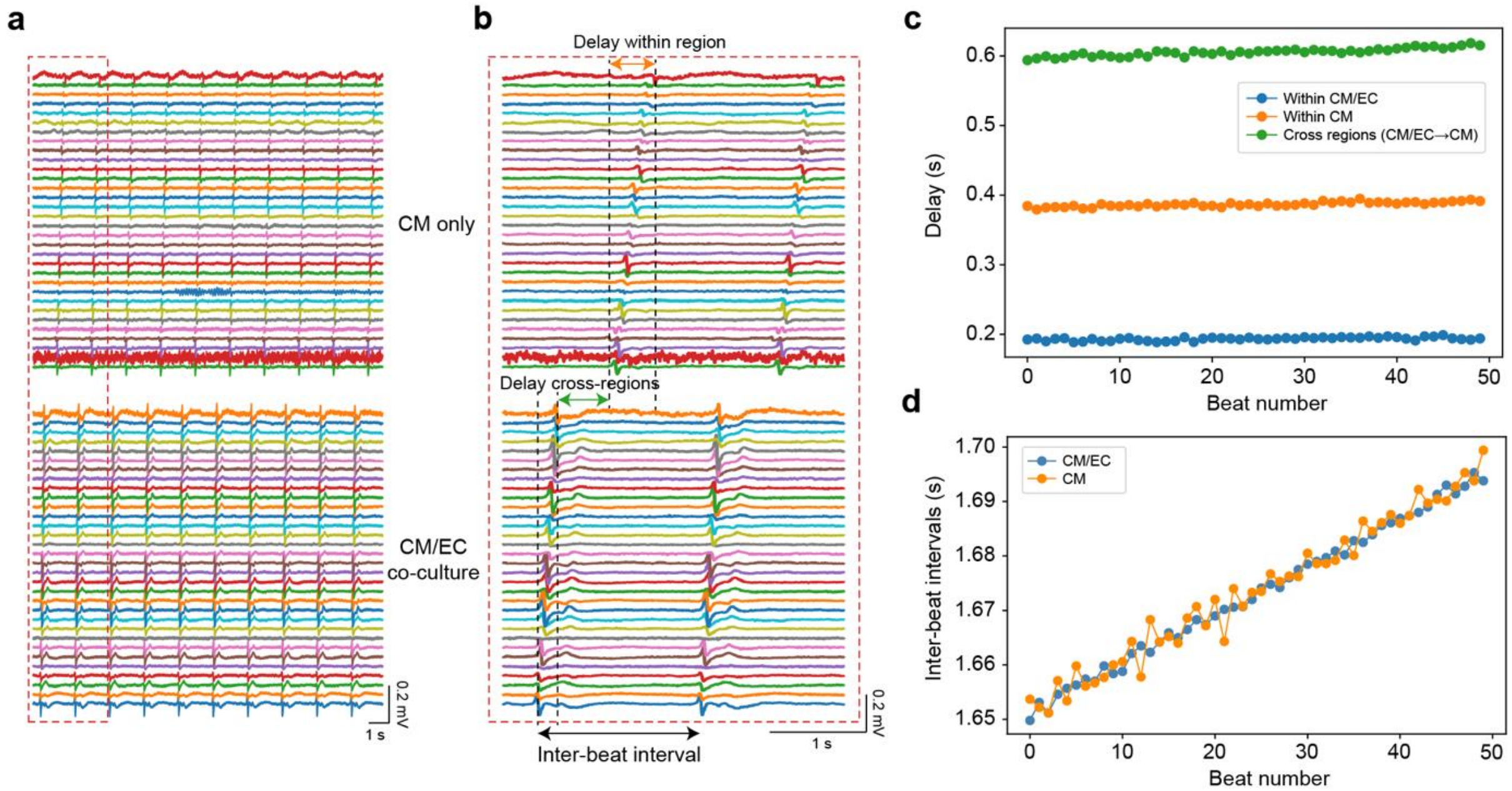

Response Fig. 4-6: Beating synchrony and conduction delay between CM/EC and CM regions. a, Representative voltage traces showing rhythmic depolarizations recorded from CM/EC (bottom) and CM (top) regions. **b**, Zoom-in view of two consecutive beats with annotation of measurement criteria. **c**, Beat-by-beat conduction delays within the CM/EC region (blue), within the CM region (orange), and across the two regions (green). The cross-region delay (CM/EC → CM) remained stable at ~0.6 s, while within-region delays were shorter (0.19 s and 0.39 s, respectively). **d**, Beat-to-beat inter-beat intervals of CM/EC and CM regions showing identical temporal trends with a gradual, parallel increase over time, indicating a shared rhythm and electrical coupling across the regions.

Revision to the manuscript:

We added the **Response Fig. 4-6** to the Supplementary Information as **Supplementary Fig. 7** and discussed the results in the main text.

"The two domains exhibited coordinated rhythmic activity with a consistent conduction delay across the interface (Supplementary Fig. 7), indicating partial electrical coupling between regions, which is consistent with the intended design of a spatially heterogeneous yet functionally integrated tissue model."

Comment-11: The authors highlight interesting genes upregulated across different niches, such as ion channels. What about their proteins, can those be validated? Seems compatible with their technology.

Response: We appreciate the reviewer's thoughtful comment. While combining *in situ* sequencing with immunostaining of selected proteins in the same tissue section has been demonstrated using STARmap PLUS[10], this approach was optimized for thin brain slices (~20 μm), where antibody and enzyme diffusion could be well controlled. In our study, the cardiac tissues were substantially thicker, which significantly limits the penetration of primary and secondary antibodies. In addition, optical scattering in thick 3D tissues further complicates high-resolution confocal imaging. In the current *in situ* graphene-seq workflow, tissues are embedded within hydrogel and subjected to enzymatic digestion. These steps would disrupt the native protein architecture and epitopes essential for immunostaining. Therefore, direct validation of protein expressions within the same intact organoids using the current protocol is not readily feasible.

Moreover, even when technically feasible, quantitative comparison between RNA abundance and protein levels is inherently complex. Protein expression is influenced by post-transcriptional regulation, translation efficiency, protein translocation and degradation dynamics[11,12]. Consequently, protein abundance does not necessarily scale linearly with transcript levels, particularly for ion channels and membrane proteins, whose localization and turnover are tightly regulated[13]. Establishing quantitative correspondence between RNA and protein levels in thick 3D tissues would require a distinct, carefully optimized, and benchmarked workflow, which remains an active area of investigation.

We agree that future advances enabling compatible protein detection within this workflow would provide valuable confirmation of key targets identified at the electrophysiological and transcriptomic levels.

Revision to the manuscript:

We added this point to the Discussion as a promising direction to further expand the utility of the platform.

"There are several promising directions to further expand the capabilities of in situ graphene-seq. Integration with real-time functional measurements, including mechanophysiological readouts from graphene-based biosensors[45], could further expand its dimensionality. Advances in spatial omics, including spatial transcriptomics with proteins imaging[46], transcriptome-translatome co-profiling, and sequencing of intact organoids or tissue blocks[47], are also promising to synergize with this platform."

Comment-12: Fig.1a **-** instead of using the word 'ch' use 'Channel'

Response: We thank the reviewer for this helpful suggestion. The abbreviation "ch" in Fig. 1a has been replaced with the full term "Channel" in the revised figure for clarity.

REDACTED

Revised Fig. 1a, Arrays of stretchable mesh nanoelectronics with transparent graphene/PEDOT:PSS electrodes are integrated with stem cell-derived microtissues for chronic electrophysiological recordings, followed by tissue clearing and in situ sequencing. Fluorescent E-barcodes enable the alignment of electrophysiological recordings with spatial gene expression mapping.

Comment-13: Fig.2j – is the decrease in FPD indicate maturation, or is there a decrease in viability of the cells in the mesh

Response: We thank the reviewer for this important question. The modest decrease in FPD observed between the first and second recording sessions reflects electrophysiological maturation rather than a loss of cell viability. Spike amplitude and maximum dV/dt remained stable across all sessions (**Fig. 2h–i**), and the number of active channels did not decline. A reduction in viability would typically result in diminished amplitude, slower upstrokes, and irregular or asynchronous beating—none of which were observed.

Moreover, shortening of action-potential and field-potential duration with maintained excitability is a well-recognized hallmark of cardiomyocyte maturation[14,15].

Comment-14: Fig.3b - Antibody labels are blurry, and CD31 expression in the co-culture sample seems skewed on one side – is this at the co-culture to CM-only interface? If so, this should be indicated.

Response: We thank the reviewer for this observation. We have adjusted the text-background contrast to improve the clarity of the antibody labels in **Fig. 3b**.

Regarding CD31 signal, we confirmed that the images were not acquired at the interface between the CM-only and CM/EC co-culture regions. The asymmetry in CD31 expression reflects the natural clustering of endothelial cells within the CM/EC co-culture, rather than a positional effect at a tissue boundary.

We also confirmed that all fluorescence images were acquired using identical microscopy parameters and were processed using the same display settings (min/max, brightness, and contrast) in Fiji. The distinct, heterogeneous CD31 signal confirms the successful integration of endothelial cells within the engineered tissue model.

Revision to the manuscript:

We have revised **Fig. 3b** to improve contrast and labeling and added the imaging acquisition and processing parameters to the figure caption.

b

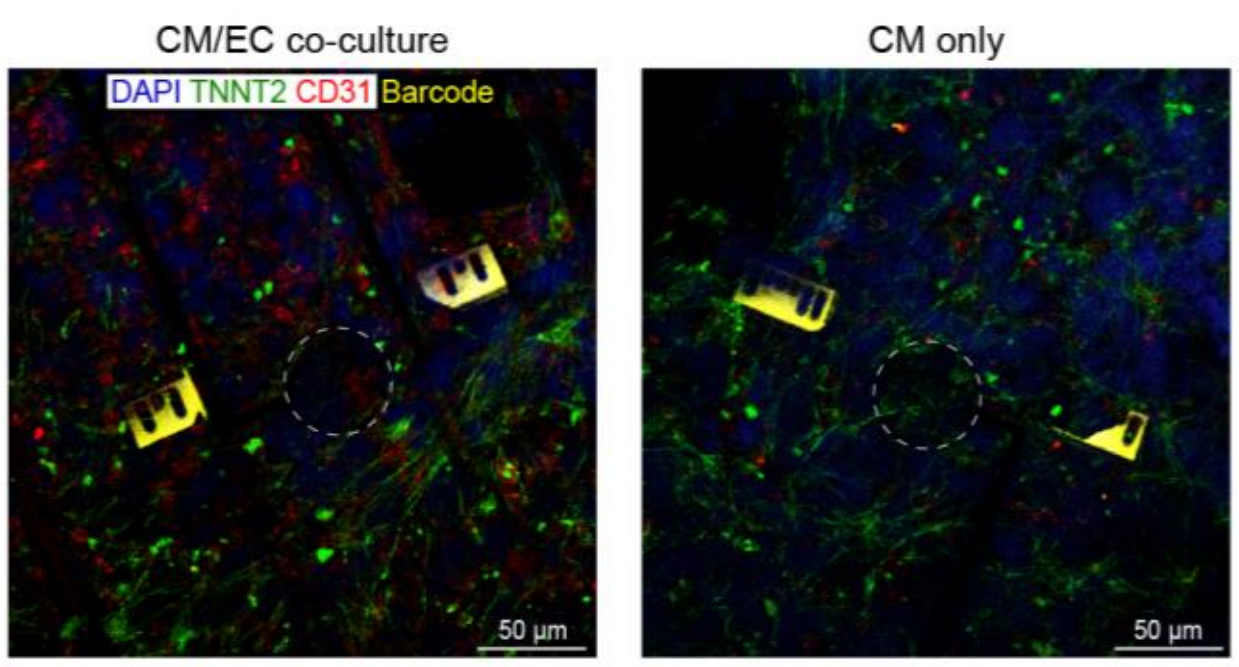

Revised Fig. 3b, *"Confocal fluorescence images showing immunohistochemically stained cells near electrodes in the CM/EC co-culture region and the CM only region. Images were acquired using identical microscopy settings and processed using consistent display parameters, including min/max intensity, brightness, and contrast."*

Comment-15: Fig.5f – Need better contrast

Response: We thank the reviewer for this comment. We have updated **Fig. 5f** to improve the image contrast and visibility.

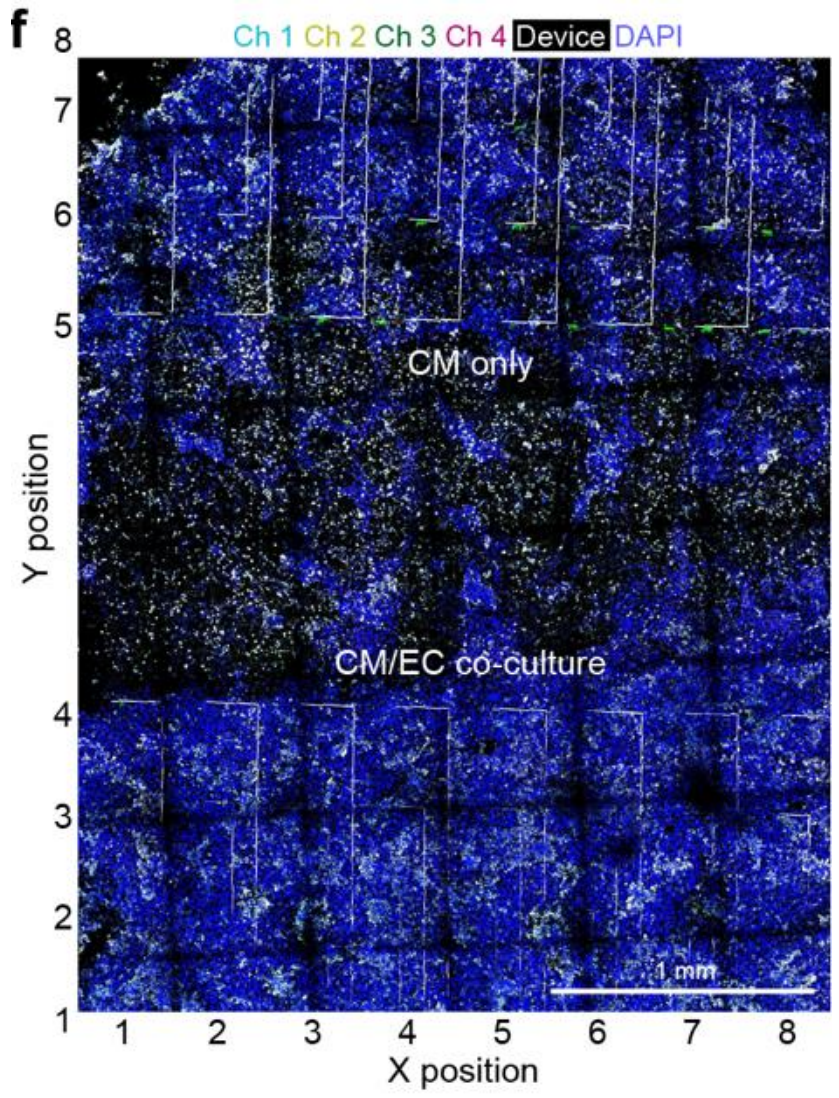

Revised Fig. 5f, Representative in situ sequencing image of a heterogeneous microtissue containing CM only and CM/EC regions, with relative X and Y positions of electrodes.

Comment-16: In line 195, the authors relate higher spike amplitudes, greater maximum dV/dt, and shorter FPD observed in CM/EC culture to greater functional maturation. What is the evidence that these parameters are associated with maturation in vivo?

Response: We thank the reviewer for this valuable comment. The extracellular field potential parameters reported here have been widely used as surrogates for the underlying transmembrane action potentials. In particular, spike amplitude and maximum dV/dt primarily correlate with the kinetics of the inward Na^+ current responsible for rapid depolarization phase, while the field potential duration (FPD) corresponds to the action potential duration (APD_{90}) and reflects repolarization dynamics[16,17].

In vivo developmental studies have shown that cardiomyocyte maturation is accompanied by increased expression of sodium channels and L-type calcium channels, resulting in faster upstroke velocities, larger action potential amplitudes, and progressively shorter action potential durations during development[18,19].

In addition, comparative analyses benchmarking hPSC-derived cardiomyocytes against fetal and adult human cardiomyocytes demonstrate that more mature phenotypes are characterized by these same electrophysiological features, including higher action potential amplitude, faster upstroke, and shorter action potential duration[15].

Although electrophysiological measurements do not capture all aspects of cardiomyocyte maturation, these parameters are known to change during *in vivo* cardiomyocyte maturation.

Revision to the manuscript:

We have cited these developmental benchmarks in the revised manuscript.

"Waveform feature analysis indicated enhanced electrical maturation of hiPSC-CMs in the CM/EC co-culture region, with higher spike amplitudes, greater maximum dV/dt, and shorter FPD[23,36,37], compared to the CM-only region (Fig. 3e–g)."

Response to Reviewer #5 Comment

I co-reviewed this manuscript with one of the reviewers who provided the listed reports. This is part of the Nature Communications initiative to facilitate training in peer review and to provide appropriate recognition for Early Career Researchers who co-review manuscripts.

Response: We thank the reviewer for their time and contribution to the evaluation of our manuscript.

References

1. Scala, F. et al. Phenotypic variation of transcriptomic cell types in mouse motor cortex. *Nature* **598**, 144–150 (2021).

2. Cadwell, C. R. et al. Electrophysiological, transcriptomic and morphologic profiling of single neurons using Patch-seq. *Nat. Biotechnol.* **34**, 199–203 (2016).

3. Li, Q. et al. Multimodal charting of molecular and functional cell states via in situ electro-sequencing. *Cell* **186**, 2002–2017 (2023).

4. Wang, X. et al. Three-dimensional intact-tissue sequencing of single-cell transcriptional states. *Science* **361**, eaat5691 (2018).

5. Spira, M. E.& Hai, A. Multi-electrode array technologies for neuroscience and cardiology. *Nat. Nanotechnol.* **8**, 83–94 (2013).

6. Herrero, D. et al. The Vascular Niche for Adult Cardiac Progenitor Cells. *Antioxidants* **11**, 5, 882 (2022).

7. Butler, J. M., Kobayashi, H.& Rafii, S. Instructive role of the vascular niche in promoting tumour growth and tissue repair by angiocrine factors. *Nat. Rev. Cancer* **10**, 138–146 (2010).

8. Mauger, S. A.& Moulé, A. J. Characterization of new transparent organic electrode materials. *Org. Electron.* **12**, 1948–1956 (2011).

9. Kong, M. et al. Advanced Optical Characterization of PEDOT:PSS by Combining Spectroscopic Ellipsometry and Raman Scattering. *ACS Omega* **7**, 39429–39436 (2022).

10. Zeng, H. et al. Integrative in situ mapping of single-cell transcriptional states and tissue histopathology in a mouse model of Alzheimer's disease. *Nat. Neurosci.* **26**, 430–446 (2023).

11. Vogel, C.& Marcotte, E. M. Insights into the regulation of protein abundance from proteomic and transcriptomic analyses. *Nat. Rev. Genet.* **13**, 227–232 (2012).

12. Schwanhäusser, B. et al. Global quantification of mammalian gene expression control. *Nature* **473**, 337–342 (2011).

13. Sun, X., Zaydman, M. A.& Cui, J. Regulation of voltage-activated K+ channel gating by transmembrane β subunits. *Front. Pharmacol.* **3**, 63 (2012).

14. Zhu, H. et al. Two dimensional electrophysiological characterization of human pluripotent stem cell-derived cardiomyocyte system. *Sci. Rep.* **7**, 43210 (2017).

15. Feric, N. T.& Radisic, M. Maturing human pluripotent stem cell-derived cardiomyocytes in human engineered cardiac tissues. *Adv. Drug Deliv. Rev.* **96**, 110–134 (2016).

16. Clements, M.& Thomas, N. High-Throughput Multi-Parameter Profiling of Electrophysiological Drug Effects in Human Embryonic Stem Cell Derived Cardiomyocytes Using Multi-Electrode Arrays. *Toxicol. Sci.* **140**, 445–461 (2014).

17. Tertoolen, L. G. J. et al. Interpretation of field potentials measured on a multi electrode array in pharmacological toxicity screening on primary and human pluripotent stem cell-derived cardiomyocytes. *Biochem. Biophys. Res. Commun.* **497**, 1135–1141 (2018).

18. Yang, X., Pabon, L.& Murry, C. E. Engineering Adolescence. *Circ. Res.* **114**, 511–523 (2014).

19. Veerman, C. C. et al. Immaturity of Human Stem-Cell-Derived Cardiomyocytes in Culture: Fatal Flaw or Soluble Problem? *Stem Cells Dev.* **24**, 1035–1052 (2015).

REVIEWERS' COMMENTS

Response: We sincerely thank the Editor and all reviewers for their careful evaluation of our manuscript and for their constructive feedback, which has helped strengthen this work. Below, we provide a point-by-point response to the remaining comments.

Reviewer #1:

The authors have properly responded to my concerns and I have no further questions.

Response: We thank the reviewer for their positive evaluation and for their constructive feedback throughout the review process, which has helped improve the quality of our work.

Reviewer #3 (Remarks to the Author):

The manuscript addresses my concerns. I have no further comments.

Response: We thank the reviewer for their positive evaluation and for their constructive feedback throughout the review process, which has helped improve the quality of our work.

Reviewer #4 (Remarks to the Author):

All my comments have been addressed, except side-by-side comparisons with the author's previous technology iteration. The revised manuscript is therefore acceptable for publication, but the authors are still suggested to make minor revisions as follows:

Response: We thank the reviewer for their positive evaluation and for their insightful comments, which have helped improve the quality of our work. Please find below our point-by-point responses to the remaining comments.

1. The authors argue that in their previous approach, which relied on opaque metal electrodes, the additional handling steps required "often disrupted the native cell microenvironment" such that "successful imaging becomes highly variable" – can examples be provided? i.e. of native cell microenvironment disruptions and unsuccessful imaging due to the extra steps, or quantification of imaging success variability.

Response: We thank the reviewer for this insightful comment. To provide examples of potential disruption to the native cell microenvironment, we performed a flip test in which samples were imaged before and after detaching and flipping the tissue (**Response Fig. 4-1a**).

Response Fig. 4-1b, c show representative outcomes from two different samples. In one case (**Response Fig. 4-1b**), the DAPI-stained nuclei distribution is well preserved after flipping, demonstrating that this approach can maintain the native microenvironment and enable successful imaging. In another case (**Response Fig. 4-1c**), however, noticeable changes in nuclei distribution are observed following the same procedure, indicating partial disruption of the tissue microstructure.

These results suggest that while the previous approach can yield high-quality imaging, the outcome can be sensitive to handling conditions, such as mechanical stress during detachment and fluid manipulation during flipping and transfer. This leads to variability in preserving the native microenvironment across samples.

Our approach is designed to minimize such handling-dependent variability by eliminating the need for these additional steps, thereby improving consistency while maintaining tissue integrity.

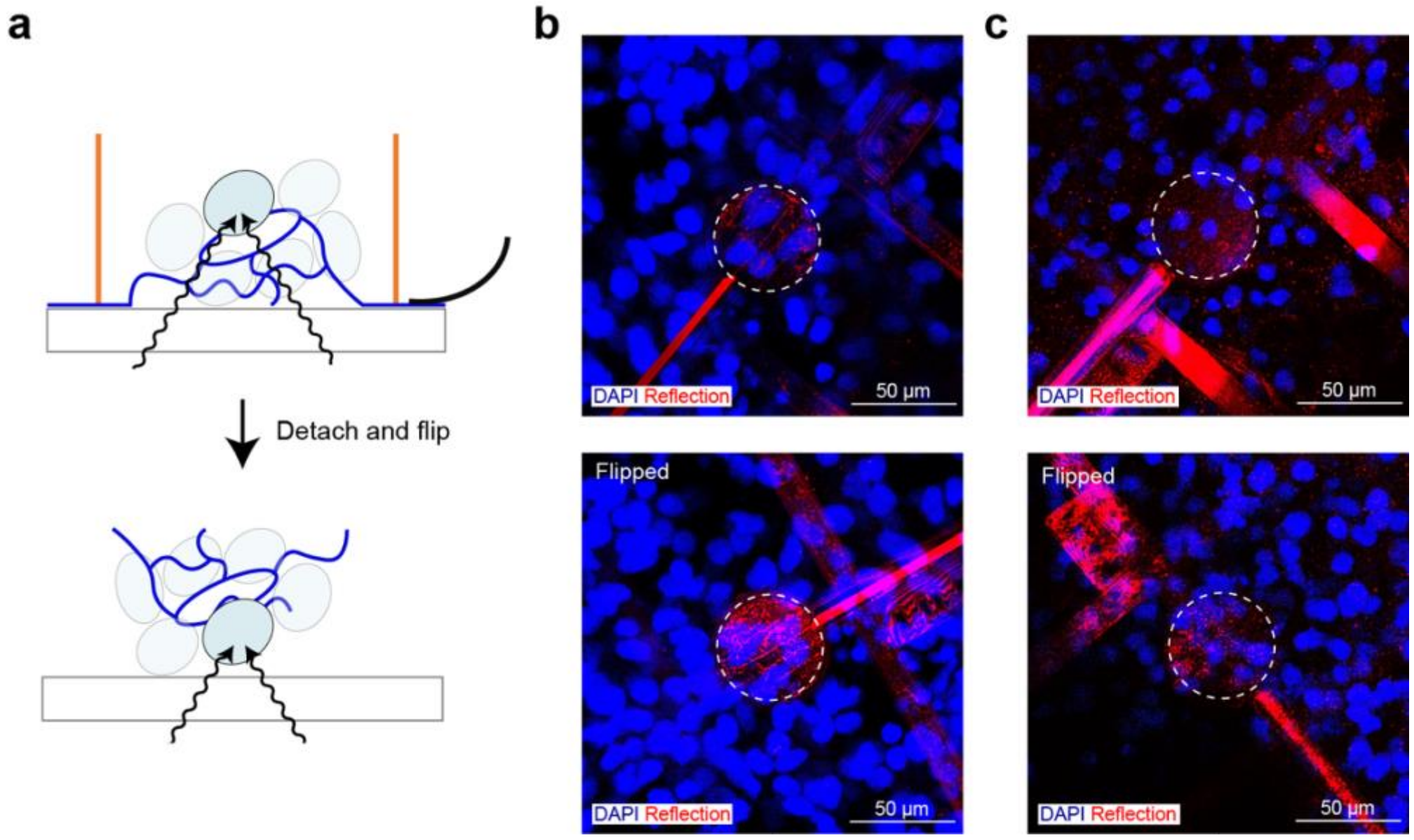

Response Fig. 4-1: Image comparison before and after detachment and flipping of the tissue/electronics hybrid. a, Schematic showing the flip test procedure. The same electrode region within the tissue/electronics hybrid was imaged, followed by detachment from the substrate and flipping of the sample for re-imaging. **b**, Representative confocal microscopy images from one sample before (top) and after (bottom) flipping, showing preservation of DAPI-stained nuclei distribution near the electrode region. **c**, Representative images from a second sample before (top) and after (bottom) flipping, showing altered nuclei distribution after the same procedure.

Revision to the manuscript:

We have clarified the potential causes of microenvironment disruption.

"These steps introduced substantial handling variability and could disrupt the native cell microenvironment due to mechanical stress during detachment, as well as hydrodynamic forces during flipping and transfer."

2. Is it possible to apply the new Supplementary Fig. 4C quantification to the previous (opaque electrodes) approach, to compare their imaging resolution side-by-side?

Response: We thank the reviewer for this important suggestion. We agree that a side-by-side comparison would provide a more direct assessment of imaging performance. However, unlike graphene/PEDOT:PSS electrodes, metal electrodes block the optical path, making the Sobel gradient magnitude-based resolution analysis (used in previous Supplementary Fig. 4c) not directly applicable under these conditions. When the optical signal is significantly attenuated, gradient-based metrics can be dominated by intensity discontinuities introduced by the electrode itself, rather than reflecting true image resolution.

To enable a more appropriate comparison, we instead quantified pixel intensity within the electrode regions and compared signals from cells located above and below the electrodes. Specifically, we analyzed DAPI-stained nuclei signal and calculated the mean and standard deviation of pixel intensities in these two regions across five electrodes.

As shown in **Response Fig. 4-2a-c**, graphene/PEDOT:PSS electrodes exhibit no substantial difference in pixel intensity between regions above and below the electrode (mean ± S.D. 16.27 ± 5.30 above vs. 18.09

± 6.80 below), indicating minimal impact on the imaging path. In contrast, metal electrodes show a noticeable reduction in signal intensity for cells above the electrode (13.43 ± 3.62 above vs. 44.29 ± 10.95 below), consistent with attenuation of the optical signal (**Response Fig. 4-2d-f**).

Together, these results provide a direct side-by-side comparison and support that graphene/PEDOT:PSS electrodes enable more consistent imaging across the electrode region, while metal electrodes introduce optical obstruction.

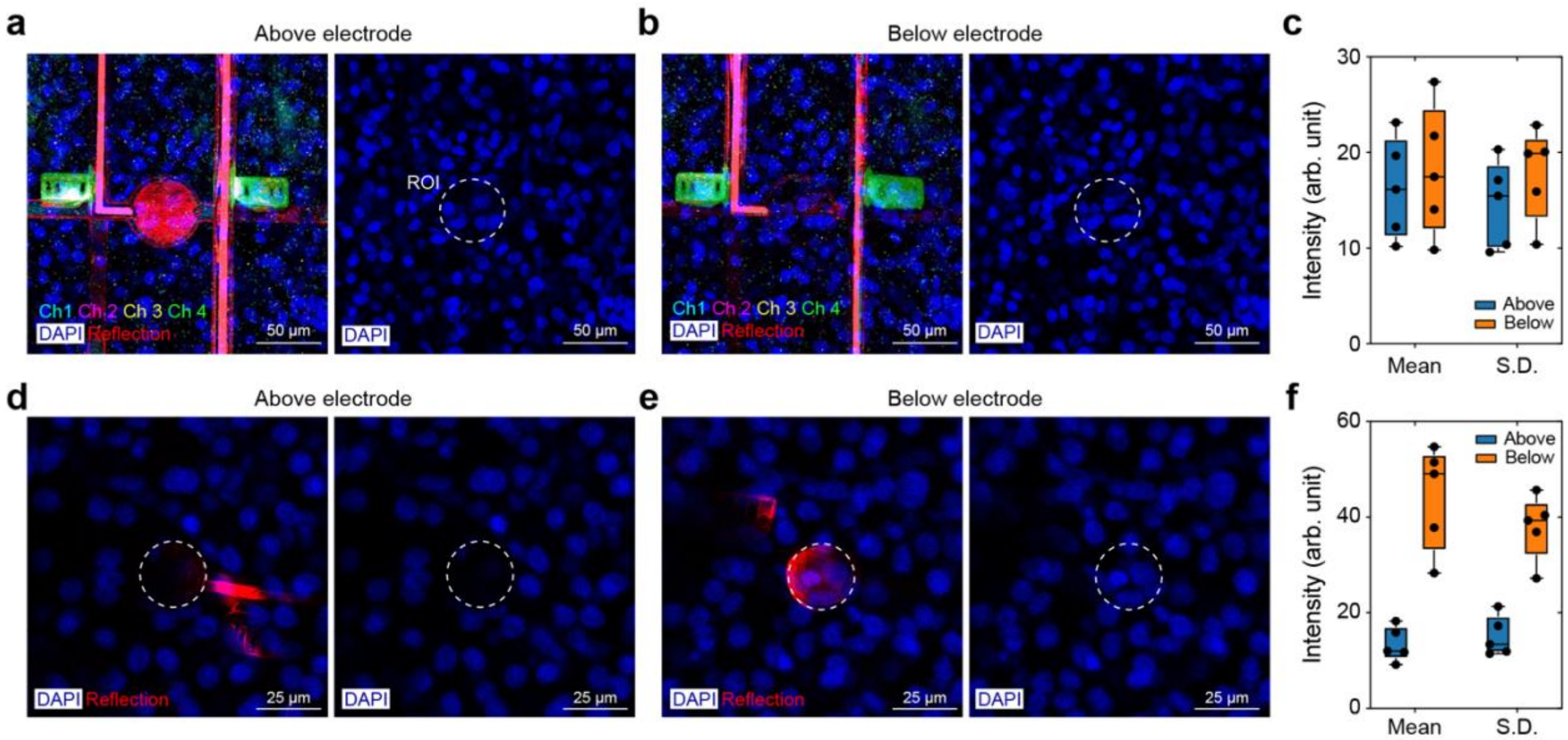

Response Fig. 4-2: Optical accessibility enabled by transparent electrodes. a–b, Representative confocal images of cells integrated with transparent electrode arrays. Left panels show composite views, including reflection mode (red) to verify electrode location relative to the region of interest (ROI). Right panels show the corresponding DAPI channel used for analysis. **a**, Cells above the electrode. **b**, Cells below the electrode. **c**, Quantification of DAPI pixel intensity within the same ROI for regions above and below the electrode (*n*=5 electrodes). **d–e**, Representative confocal images of cells integrated with metal electrode arrays, displayed as in (a–b). **f**, Quantification of DAPI pixel intensity within the same ROI for regions above and below the electrode (*n*=5 electrodes).

Revision to the manuscript:

We have incorporated **Response Fig. 4-2** into the Supplementary Information as **Supplementary Fig. 4** and added the following statement to the main text.

"Consistent with the optical transparency of the electrodes, imaging signals from cells above the electrodes were largely preserved, whereas metal electrodes exhibited noticeable signal attenuation (Supplementary Fig. 4)."

Reviewer #5 (Remarks to the Author):

I co-reviewed this manuscript with one of the reviewers who provided the listed reports. This is part of the Nature Communications initiative to facilitate training in peer review and to provide appropriate recognition for Early Career Researchers who co-review manuscripts.

Response: We thank the reviewer for their time and contribution to the evaluation of our manuscript.

Reviewer #6 (Remarks to the Author):

I co-reviewed this manuscript with one of the reviewers who provided the listed reports. This is part of the Nature Communications initiative to facilitate training in peer review and to provide appropriate recognition for Early Career Researchers who co-review manuscripts.

Response: We thank the reviewer for their time and contribution to the evaluation of our manuscript.

Reviewer #6 (Remarks on code availability):

The code link provided a README file with instructions. I would encourage the authors to include more notes to improve readability. The code can be reproduced based on the code posted on GitHub.

Response: We thank the reviewer for this helpful suggestion. We have updated the README file on GitHub to improve clarity and readability.
